# *In vitro* and *in vivo* drug screens of tumor cells identify novel therapies for high‐risk child cancer

**DOI:** 10.15252/emmm.202114608

**Published:** 2021-12-20

**Authors:** Loretta M S Lau, Chelsea Mayoh, Jinhan Xie, Paulette Barahona, Karen L MacKenzie, Marie Wong, Alvin Kamili, Maria Tsoli, Tim W Failes, Amit Kumar, Emily V A Mould, Andrew Gifford, Shu‐Oi Chow, Mark Pinese, Jamie I Fletcher, Greg M Arndt, Dong‐Anh Khuong‐Quang, Carol Wadham, Daniel Batey, Georgina Eden, Peter Trebilcock, Swapna Joshi, Stephanie Alfred, Anjana Gopalakrishnan, Aaminah Khan, Dylan Grebert Wade, Patrick A Strong, Elodie Manouvrier, Lisa T Morgan, Miriam Span, Jin Yi Lim, Roxanne Cadiz, Caitlin Ung, David M Thomas, Katherine M Tucker, Meera Warby, Geoffrey B McCowage, Luciano Dalla‐Pozza, Jennifer A Byrne, Federica Saletta, Andrew Fellowes, Stephen B Fox, Murray D Norris, Vanessa Tyrrell, Toby N Trahair, Richard B Lock, Mark J Cowley, Paul G Ekert, Michelle Haber, David S Ziegler, Glenn M Marshall

**Affiliations:** ^1^ Children’s Cancer Institute, Lowy Cancer Centre UNSW Sydney Kensington NSW Australia; ^2^ School of Women’s and Children’s Health UNSW Sydney Kensington NSW Australia; ^3^ Kids Cancer Centre Sydney Children’s Hospital Randwick NSW Australia; ^4^ ACRF Drug Discovery Centre for Childhood Cancer Children’s Cancer Institute Lowy Cancer Research Centre UNSW Sydney Sydney NSW Australia; ^5^ Children’s Cancer Centre Royal Children’s Hospital Parkville Vic. Australia; ^6^ Murdoch Children’s Research Institute Royal Children’s Hospital Parkville Vic. Australia; ^7^ Kinghorn Cancer Centre Garvan Institute of Medical Research Darlinghurst NSW Australia; ^8^ Faculty of Medicine St Vincent’s Clinical School UNSW Sydney Kensington NSW Australia; ^9^ Hereditary Cancer Centre Prince of Wales Hospital Randwick NSW Australia; ^10^ Prince of Wales Hospital Clinical School UNSW Sydney Randwick NSW Australia; ^11^ Cancer Centre for Children The Children’s Hospital at Westmead Westmead NSW Australia; ^12^ Children’s Cancer Research Unit Kids Research Westmead NSW Australia; ^13^ Faculty of Medicine and Health The University of Sydney NSW Australia; ^14^ Peter MacCallum Cancer Centre Melbourne Vic. Australia; ^15^ Department of Medical Oncology University of Melbourne Melbourne Vic. Australia; ^16^ University of New South Wales Centre for Childhood Cancer Research UNSW Sydney Kensington Vic. Australia; ^17^ Kinghorn Centre for Clinical Genomics Garvan Institute of Medical Research Darlinghurst NSW Australia

**Keywords:** drug screen, patient‐derived xenograft, pediatric cancer, precision medicine, Cancer, Chromatin, Transcription & Genomics, Pharmacology & Drug Discovery

## Abstract

Biomarkers which better match anticancer drugs with cancer driver genes hold the promise of improved clinical responses and cure rates. We developed a precision medicine platform of rapid high‐throughput drug screening (HTS) and patient‐derived xenografting (PDX) of primary tumor tissue, and evaluated its potential for treatment identification among 56 consecutively enrolled high‐risk pediatric cancer patients, compared with conventional molecular genomics and transcriptomics. Drug hits were seen in the majority of HTS and PDX screens, which identified therapeutic options for 10 patients for whom no targetable molecular lesions could be found. Screens also provided orthogonal proof of drug efficacy suggested by molecular analyses and negative results for some molecular findings. We identified treatment options across the whole testing platform for 70% of patients. Only molecular therapeutic recommendations were provided to treating oncologists and led to a change in therapy in 53% of patients, of whom 29% had clinical benefit. These data indicate that *in vitro* and *in vivo* drug screening of tumor cells could increase therapeutic options and improve clinical outcomes for high‐risk pediatric cancer patients.

## Graphical abstract

A precision diagnostic platform integrating genomics and transcriptomics with drug testing of patient's primary tumor cells in high throughput drug screening (HTS) and patient‐derived xenograft (PDX) was established to improve identification of therapies in high‐risk pediatric cancer patients.

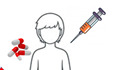

**The paper explained**

**Problem**

Next‐generation sequencing can identify molecular targets and permit personalized choice of drug treatment for children with cancer. However, the clinical uptake of therapeutic recommendations has been low, suggesting the need for orthogonal proofs using other techniques to support the molecular recommendation and thus clinical decision‐making. A diagnostic platform integrating genomics and transcriptomics with drug testing of patient’s primary tumor cells in high‐throughput drug screening (HTS) and patient‐derived xenografting (PDX) has the potential to improve identification of targeted therapies and clinical uptake.

**Results**

A precision medicine platform incorporating rapid HTS and PDX of primary tumor tissue was developed and evaluated in 56 high‐risk pediatric cancer patients with an expected survival of less than 30%. Across the whole testing platform, treatment options were identified for 70% of patients. Fresh tumor tissue allowed HTS and/or PDX for 52% of patients. Drug hits were present in the majority of HTS, which provided orthogonal proof of drug efficacy suggested by molecular analyses and negative results for some molecular findings. Effective treatments were also observed in over half of PDX models. Therapeutic options were found in 10 patients for whom no targetable molecular lesions could be identified by genomics and transcriptomics. Only molecular therapeutic recommendations were provided to treating oncologists and led to a change in therapy in 53% of patients, of whom 29% had a clinical benefit. There was a strong correlation between HTS and PDX results, and the clinical responses in patients.

**Impact**

This study represents the first pediatric precision oncology study which has integrated genomics, transcriptomics, *in vitro* and *in vivo* drug efficacy testing to derive therapeutic options for high‐risk pediatric cancer patients. This comprehensive approach is feasible and has the potential to expand therapeutic options, increase clinical uptake, and improve clinical outcomes for these patients.

## Introduction

The development of targeted anticancer therapies has suggested the goal of more personalized treatment approaches aimed at better matching the patient’s driver genes to therapeutics. Two recent pediatric cancer genomic landscape studies highlight the differences between pediatric and adult cancers, including a contrasting spectrum of genomic driver events (Grobner *et al*, 2018; Ma *et al*, 2018). Evaluation of next‐generation sequencing in pediatric cancers reports targetable molecular findings in 34–87% of cases (Mody *et al*, 2015; Chang *et al*, 2016; Harris *et al*, 2016; Oberg *et al*, 2016; Parsons *et al*, 2016; Worst *et al*, 2016; Harttrampf *et al*, 2017; Pincez *et al*, 2017; Khater *et al*, 2019). However, the clinical uptake of therapeutic recommendations was only 10–38% of patients (Mody *et al*, 2015; Harris *et al*, 2016; Worst *et al*, 2016). There are many reasons for this relatively low clinical uptake, including drug access and insufficient evidence supporting the recommendation. One strategy to improve confidence in personalized therapeutic recommendations is the inclusion of functional analyses of primary patient tumor cells exposed to potential therapeutics (Pemovska *et al*, 2013; Friedman *et al*, 2015; Letai, 2017; Pauli *et al*, 2017). We hypothesized that a diagnostic platform integrating tumor genomics and transcriptomics with *in vitro* and *in vivo* drug sensitivity testing of the patient’s primary tumor cells would improve identification of targeted therapies and clinical uptake.

Here, we report the first pediatric cancer study evaluating therapeutic recommendations derived from high‐throughput drug screening (HTS) and patient‐derived xenografting (PDX) of primary tumor tissues from high‐risk pediatric cancer patients. The addition of *in vitro* and *in vivo* drug testing to a genome‐only analysis significantly increased the proportion of patients with treatment options.

## Results

### Patients and tumor samples

A total of 56 children with high‐risk cancer and an estimated cure rate < 30% were consecutively enrolled in the TARGET pilot study of the Australian ZERO Precision Childhood Cancer Program between June 2015 and October 2017 at the two pediatric hospitals (Sydney Children’s Hospital and Children’s Hospital at Westmead) in Sydney, Australia. The molecular results of 47 of these patients have been reported in conjunction with the follow‐up national trial (Wong *et al*, 2020). There was an equal distribution of patients at diagnosis and relapse/refractory, and included 48% central nervous system (CNS) tumors, 38% non‐CNS solid tumors, and 14% hematologic malignancies (HMs) (Appendix Table S1). The median survival of the cohort was 13.1 months, with 80% of patients surviving beyond 6 months from enrollment.

**Figure 1:**
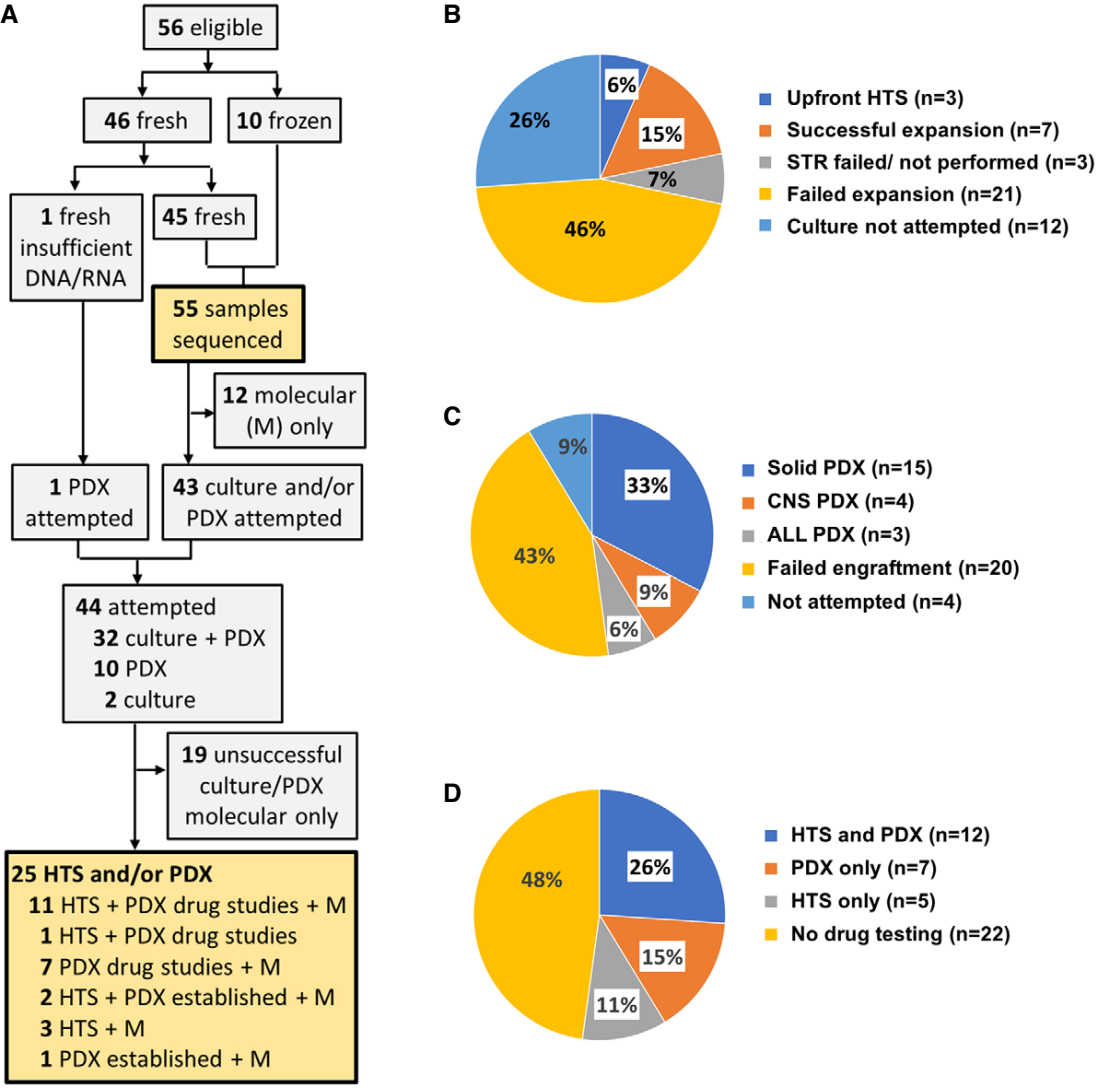
Development of preclinical models from 46 fresh samples Flow diagram of sample allocation for molecular profiling (M), primary culture, high‐throughput drug screening (HTS), and patient‐derived xenograft (PDX) drug studies.Outcome of *in vitro* expansion in 46 fresh samples.Outcome of PDX establishment in 46 fresh samples.Outcome of *in vitro* and *in vivo* drug testing attempts in 46 fresh samples.

We specified a preference for the submission of fresh tissue for HTS and PDX. Of the 56 samples, 46 were received fresh and triaged by the amount of available tissue. In the case of small solid samples (< 30 mg), the priorities after molecular (genomics and transcriptomics) analysis were primary culture for CNS tumors and PDX for non‐CNS tumors. Primary culture and/or PDX were attempted in 44 of the 46 fresh samples (Fig 1A). Hence, our study demonstrated that it was feasible to collect fresh tumor samples for a pediatric precision oncology platform.

### *In* *vitro* and *in vivo* drug screening of tumor‐derived cells is feasible in the majority of patients

Of the 46 fresh samples, adequate cell numbers allowing upfront HTS were available in only three. We therefore proceeded to *in vitro* expansion of primary tumor cells in 31 fresh samples, and developed a platform for rapid authentication by short tandem repeat (STR) profiling (i.e., confirming a culture was from the correct patient) and confirmation of the presence of tumor cells using histopathology, flow cytometry, or single‐nucleotide polymorphism (SNP) array. Of the 31 samples subjected to *in vitro* expansion, seven were successfully expanded to proceed to HTS (Fig 1B). The culture failed to proliferate in 12. Importantly, we found an absence of tumor cells in proliferating culture in nine and failed authentication in one. Culture material was not available for authentication in two. This finding illustrates the importance of culture authentication and tumor cell content validation.

We attempted PDX murine models in 42 of the 46 fresh patient samples, with successful engraftment of 22 samples (Fig 1A and C; Appendix Table S2). Non‐CNS solid tumors were subcutaneous flank models, whereas leukemia and CNS tumors were orthotopic models. Engraftment rates and time to engraftment showed variability according to tumor type. PDX engraftment was most successful in non‐CNS solid tumors/lymphoma (15/18 attempts) followed by leukemia (3/4 attempts) and CNS tumors (4/20 attempts). Successfully engrafted tumors were harvested after a median of 3.2 months (range 1.0–10.9 months; CNS 2.7 months, solid tumors 4.2 months, leukemia 1.1 months). CNS orthotopic PDX proved the most challenging to establish. Importantly, *ex vivo* expansion of cells from engrafted PDXs allowed later HTS to be performed in seven patients for whom the initial primary sample could not be cultured. Drug testing was not feasible in one patient where the PDX was very slow growing. In summary, we were able to conduct HTS and/or PDX testing (7 PDX only, 5 HTS only, and 12 PDX and HTS) in 24 of the 46 patients who provided a fresh sample (Fig 1D; Appendix Table S3).

### *In* *vitro* drug screening identifies therapies additional to those found on molecular analyses

We hypothesized that integration of HTS would enhance the identification of therapeutic options beyond those identified by molecular testing. We designed a 111‐compound screening library (63 targeted agents; 48 chemotherapeutic drugs) (Table EV1) to examine this hypothesis. These agents were chosen because they were either US Federal Drug Administration (FDA) approved (*n* = 92) or in late clinical development (*n* = 19) and were considered potentially useful in treating pediatric cancer patients based on prior clinical and preclinical evidence. Ninety of these 111 agents had existing pediatric dose and safety data.

**Figure 2:**
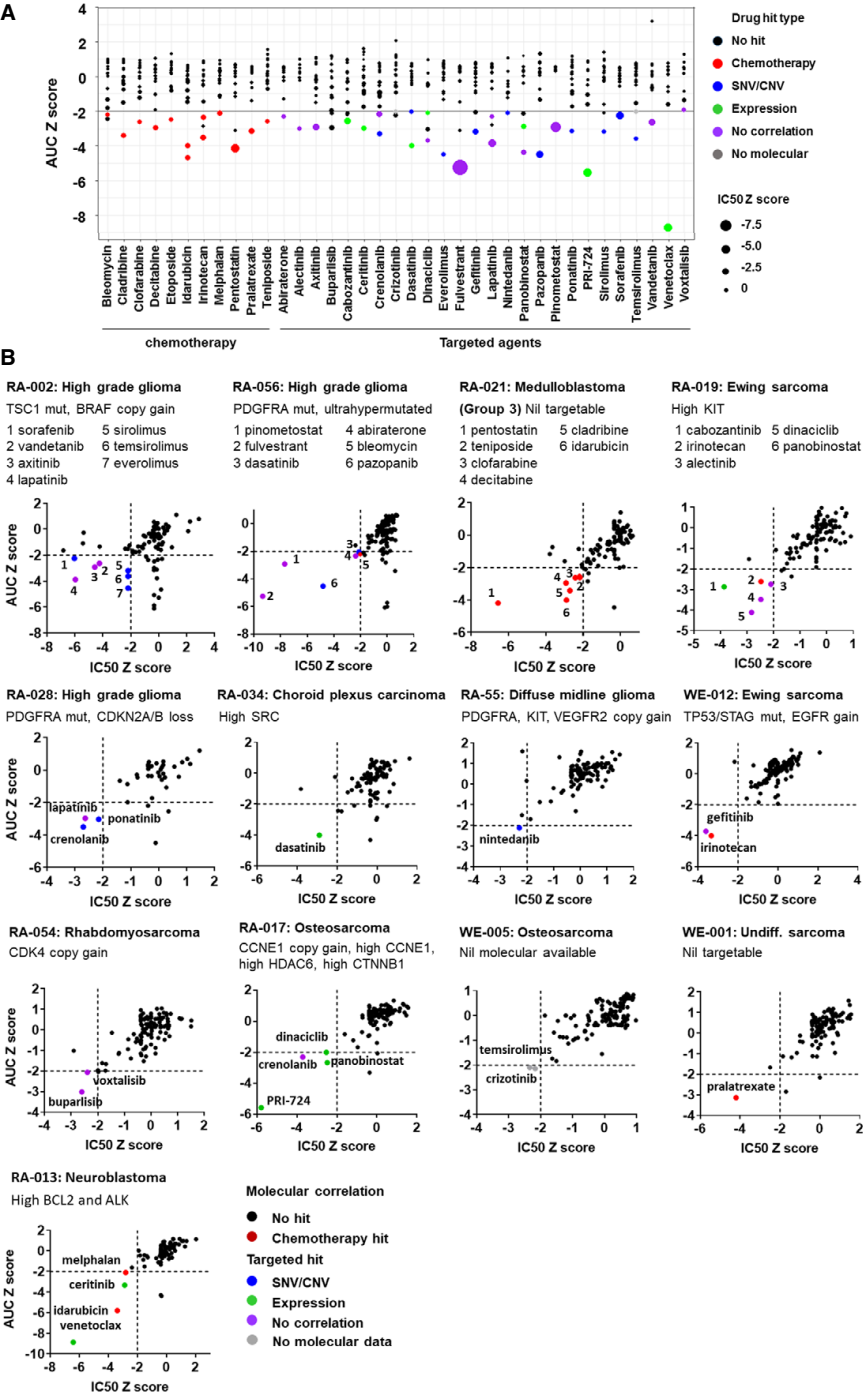
Overview of drug hits identified by high‐throughput drug screening in 13 patient‐derived samples Z score for area under the dose–response curve (AUC) and IC_50_ of 37 different drugs (shown along the horizontal axis) identified as hits in 13 of 17 samples screened. A drug hit is defined as z score of less than −2 for both AUC and IC_50_. Each dot in a column represents a sample screened for that drug. The size of the dot corresponds to the IC_50_ z score for that sample (the larger the dot, the smaller the IC_50_). Dots below the black horizontal line represent sample with AUC z score of less than −2. Dots are color coded for drug hit types. All color dots below the black line represent a hit for the corresponding drug.Plots of AUC z score against IC_50_ z score for each of the drugs screened in the 13 samples with drug hits. Source data are available online for this figure.

HTS was performed on 17 patient‐specific, STR‐authenticated cultures, of which 14 were non‐adherent cultures. Tumor cell sources included primary cells without expansion (*n* = 3), expanded primary cultures (*n* = 7), and tumor cells derived from a PDX (*n* = 7) (Appendix Table S4). Tumor cell content was confirmed in 12 of 17 samples. We used rigid criteria for identifying a drug hit, defined as a *z* score ≤ −2 for both area under the dose–response curve (AUC) and IC_50_. The HTS for each patient was completed at a median of 4.3 months (7 days–21.4 months) from sampling (Appendix Table S4). Drug hits were identified in 13 of 17 patients. The median number of drug hits per patient was 2 (range: 0–7). In total, 45 drug hits (32 targeted and 13 chemotherapy) involving 37 compounds were identified (Fig 2A; Appendix Table S5). Seventy‐one percent of hits had an IC_50_ lower than the published maximum or steady‐state plasma concentration, suggesting the IC_50_ was clinically achievable.

Of the 32 targeted drug hits, 10 correlated with molecular targets known to confer drug sensitivity to that agent found in the same patient sample (Fig 2A and B; Table 1; Appendix Table S5). This included seven hits which correlated with DNA mutations (e.g., an mTORC1 (mammalian target of rapamycin complex 1) inhibitor hit correlated with the TSC1 Asp769Ter mutation in patient RA‐002 (Tsoli *et al*, 2018) and three correlating with the copy number variation (CNV) (e.g., sensitivity to nintedanib, a tyrosine kinase inhibitor (TKI) with anti‐PDGFRA (anti‐platelet‐derived growth factor receptor alpha) and anti‐VEGFR2 (anti‐vascular endothelial growth factor receptor 2) activity, correlated with high copy number gains of PDGFRA (55 copies) and VEGFR2 (28 copies) in patient RA‐055) (Fig 2B).

**Table 1:** Correlation between high‐throughput drug screening (HTS) drug hits and prior molecular analysis.

Patient ID	Diagnosis	Drug hit	Drug target	Molecular target	HTS correlated with molecular
RA‐002	HGG	Everolimus	mTOR	TSC1 mutation with LOH	Yes
		Sirolimus	mTOR		Yes
		Temsirolimus	mTOR		Yes
		Sorafenib	Multi TKI	BRAF 6 copies and high RNA	Yes
RA‐028	HGG	Crenolanib	Multi TKI	PDGFRA mutation	Yes
		Ponatinib	Multi TKI		Yes
RA‐056	HGG	Dasatinib	Multi TKI	PDGFRA mutation	Yes
		Pazopanib	Multi TKI		Yes
RA‐034	CPC	Dasatinib	Multi TKI	High SRC RNA	Yes
RA‐055	DMG	Nintedanib	Multi TKI	PDGFRA and VEGFR2 amp	Yes
RA‐019	EWS	Cabozantinib	Multi TKI	High KIT RNA	Yes
WE‐012	EWS	Gefitinib	EGFR	EGFR 6 copies and high RNA	Yes
RA‐017	OST	Dinaciclib	CDK1/2/5/9	High CCNE1 RNA	Yes
		PRI‐724	CTNNB1	High CTNNB1 RNA	Yes
		Panobinostat	HDAC	High HDAC6 RNA	Yes
RA‐013	NBL	Ceritinib	ALK, IGF1R	High ALK and IGF1R RNA	Yes
		Venetoclax	BCL2	High BCL2 RNA	Yes
WE‐005	OST	Crizotinib	ALK, MET, ROS1	NA	
		Temsirolimus	mTOR		
RA‐002	HGG	Axitinib	multi TKI	HTS drug responses without prior molecular hallmarks for sensitivity to that drug	
		Lapatinib	ERBB2, EGFR		
		Vandetanib	multi TKI		
RA‐028	HGG	Lapatinib	ERBB2, EGFR		
RA‐056	HGG	Abiraterone	CYP17A1		
		Fulvestrant	ESR1		
		Pinometostat	DOT1L		
RA‐019	EWS	Alectinib	ALK		
		Dinaciclib	CDK1/2/5/9		
		Panobinostat	HDAC		
RA‐054	RMS	Buparlisib	PI3K		
		Voxtalisib	PI3K, mTOR		
RA‐017	OST	Crenolanib	multi TKI		

Of the 17 HTS performed, 32 molecular drug hits were identified in 11 samples.

amp, amplification; CPC, choroid plexus carcinoma; DMG, diffuse midline glioma H3 K27M mutant; EWS, Ewing’s sarcoma; HGG, high grade glioma; LOH, loss of heterozygosity; TKI, tyrosine kinase inhibitor; NA, molecular data not available; NBL, neuroblastoma; OST, osteosarcoma; RMS, rhabdomyosarcoma.

Intriguingly, we found seven examples from four patients where specific gene expression aberrations detected by bulk tumor RNA‐sequencing, independent of mutations, structural variants, or CNV, appeared to correlate with drug hits. One patient sample (RA‐013) demonstrated elevated B‐cell lymphoma 2 (BCL‐2) expression and was sensitive in HTS to the BCL‐2 inhibitor, venetoclax. Additionally, in the absence of anaplastic lymphoma kinase (ALK) fusion or mutation, this same sample with elevated ALK expression demonstrated sensitivity to the ALK inhibitor, ceritinib, with an IC_50_ well below the published maximum plasma concentration. Of interest, the sample also had increased expression of insulin‐like growth factor 1 receptor (IGF1R), a known off‐target response to ceritinib (Kuenzi *et al*, 2017).

These data from HTS provided orthogonal confirmation of a targetable molecular abnormality (mutation, copy number, expression) in 17 of the 32 targeted hits (Table 1). Importantly, the remaining 15 hits represented drug responses without prior molecular hallmarks of sensitivity to that drug. In contrast, HTS did not predict sensitivity of five samples to drugs suggested by molecular analyses. This suggests HTS might also be utilized to avoid ineffective therapy.

### Combination and single agent sensitivities are revealed on *in vivo* drug testing

We next evaluated the feasibility of incorporating PDX drug efficacy testing to increase therapeutic options. As PDX drug testing could not feasibly include the full 111‐compound library, drugs used in PDX testing were prioritized by: (i) supporting molecular and/or HTS findings; (ii) prior published preclinical or clinical evidence of drug efficacy for the specific tumor type; or (iii) potential patient eligibility for an open clinical trial at the treating institution. Combination treatments were also included whenever possible, in particular combinations shown to be feasible and effective in prior clinical trials. Whenever mouse pharmacokinetics data were available, animals were dosed to achieve the most efficacious target plasma drug concentrations reported in humans.

PDX drug efficacy testing was conducted in 19 PDX models (16 non‐CNS and 3 CNS) and combination treatments were included in 16 models (Appendix Tables S6 and S7). A total of 75 treatments were tested in these 19 PDXs and 22 of these treatments were based on prior molecular findings (Table 2). The number of treatment arms for each patient’s PDX ranged from 1 to 10 (median: 4). The duration of experiments (inoculation to predefined endpoint) ranged from 2.0 to 12.1 months (median 4.1 months). The median duration was 4.1 months (range 4.0–4.5) for the three CNS patients, 2.5 months (range 2.0–10.7) for the three leukemia patients, and 4.1 months (range 2.4–12.1) for the 13 solid tumor patients. Thus, the PDX results would have been clinically available at a median of 7.9 months (range 2.0–19.1) from the time of sampling, including establishing the PDX, secondary *in vivo* expansion and drug testing, well below the median survival duration for the cohort.

**Table 2:** Correlation between drug sensitivity predicted by molecular testing and patient‐derived xenograft (PDX) responses.

Patient ID	Diagnosis	Molecular target	PDX treatment	PDX response^a^	PDX correlated with molecular
RA‐001	EWS	TP53, STAG2 mut	IRN + TMZ + talazoparib	MCR	Yes
			Talazoparib	PD	No
WE‐012	EWS	TP53, STAG2 mut	IRN + TMZ + talazoparib	CR	Yes
			Talazoparib	PD	No
RA‐039	NBL	ALK amplification	Ceritinib	CR	Yes
			Cyclo/Topo/ceritinib	MCR	Yes
RA‐049	ALCL	NPM1 ‐ ALK	Ceritinib	CR	Yes
			Alectinib	MCR	Yes
			Brentuximab + ceritinib	MCR	Yes
RA‐002	HGG	TSC1 mut (LOH)	Temsirolimus	R	Yes
RA‐045	T‐ALL	CDKN2A/B loss	Palbociclib	SD	No
RA‐054	RMS	CDK4 amplification High FGFR4 RNA	Palbociclib	PD	No
			Palbociclib + temsirolimus	PD	No
			Ponatinib	PD	No
RA‐029	RMS	High FGFR4 RNA	Ponatinib	PD	No
RA‐017	OST	CCNE1 amplification	Dinaciclib	PD	No
			Dinaciclib + cisplatin	PD	No
RA‐013	NBL	High BCL RNA	Venetoclax	PD	No
RA‐027	NBL	NF1 mutation with LOH	Trametinib	PD	No
			Trametinib + isotretinoin	PD	No
RA‐028	HGG	PDGFRA mutation CDKN2A/B loss	Palbociclib	PD	No
			Temsirolimus + palbociclib	PD	No

ALCL, anaplastic large cell lymphoma; CR, complete response; Cyclo/Topo, cyclophosphamide/topotecan; EWS, Ewing’s sarcoma; HGG, high grade glioma; IRN, irinotecan; LOH, loss of heterozygosity; MCR, maintained complete response; NBL, neuroblastoma; OST, osteosarcoma; PD, progressive disease; R, response; RMS, rhabdomyosarcoma; SD, stable disease; T‐ALL, T‐cell acute lymphoblastic leukemia; TMZ, temozolomide.

^a^ Objective response in a non‐CNS (central nervous system) tumor is classified by Point‐to‐Point Tunneling Protocol (PPTP) criteria (Houghton *et al*, 2007). In the CNS tumor, the response is defined as significantly prolonged event‐free survival (EFS) where median EFS of the treatment group is at least twice longer than that of control and with significant difference in EFS between treated and control (*P* ≤ 0.05).

**Figure 3:**
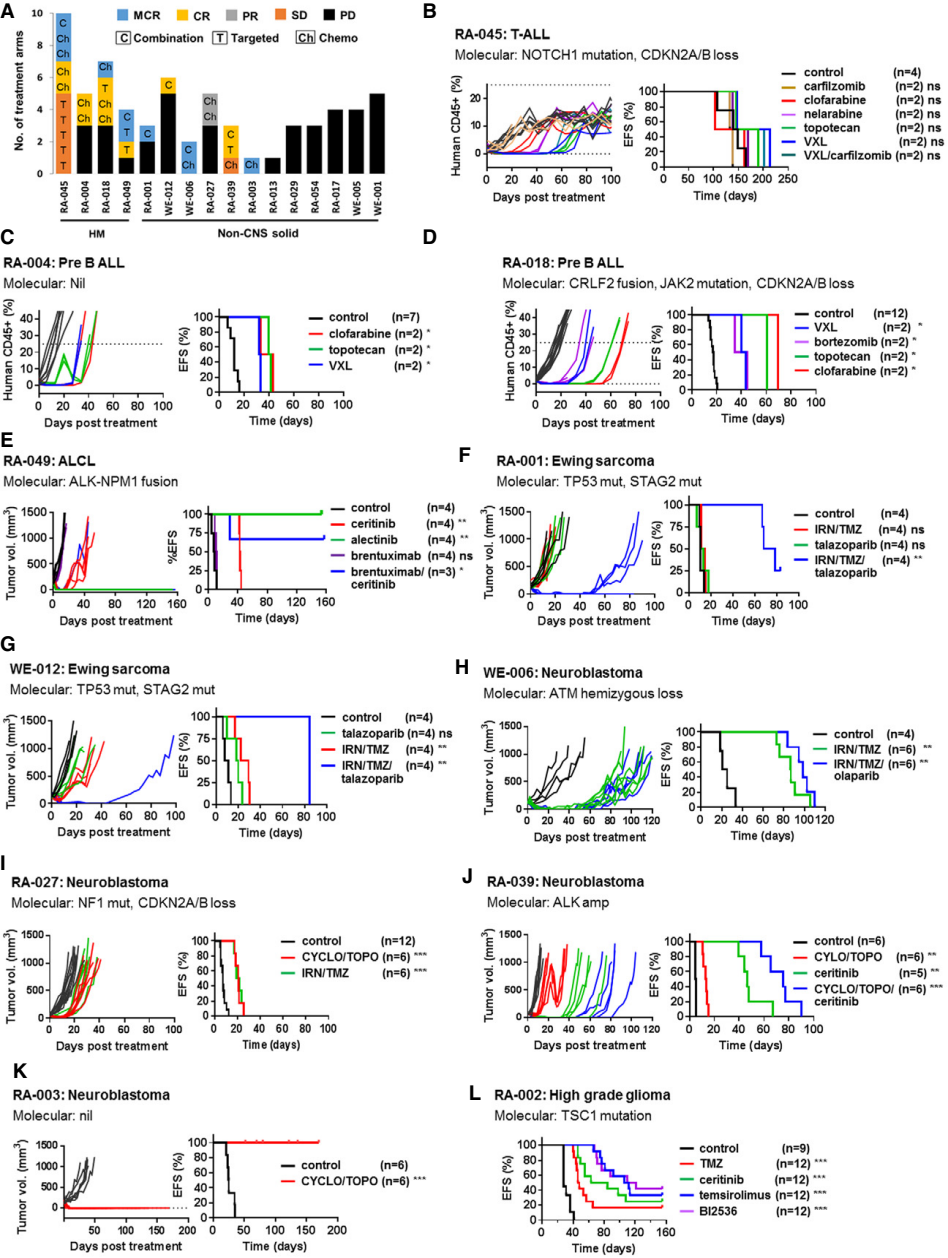
*In vivo* drug efficacy studies in patient‐derived xenografts ATreatment response in 16 hematologic malignancy (HM) and non‐CNS (central nervous system) solid patient‐derived xenograft (PDX) models. Objective responses including maintained complete response (MCR), complete response (CR), and partial response (PR) were observed in 10 of 16 models. Drugs are indicated as chemotherapy (Ch), targeted agent (T), or combination treatment (C).B–DEvent‐free survival (EFS) and percentage of human CD45^+^ leukocytes in peripheral blood in three acute lymphoblastic leukemia (ALL) orthotopic models. An event is defined as human CD45 cells above 25% in the peripheral and is represented by the dotted line.E–KEFS and tumor volume in seven non‐CNS subcutaneous PDX models which demonstrated objective response in one or more treatments.LEFS in a CNS orthotopic model in which drug sensitivity was observed. EFS is time of inoculation of tumor cells to event (defined by neurologic symptoms or weight loss). Data information: Survival curves were estimated for each treatment group using the Kaplan–Meier method and compared with the untreated control group in each PDX model statistically using log rank test. *P* value for log rank test for comparison of EFS: ns, not significant; *P < 0.05; **P < 0.01; ***P < 0.001. The exact *P* values are provided in Appendix Table S6. ALCL, anaplastic large cell lymphoma; Cyclo, cyclophosphamide; IRN, irinotecan; PD, progressive disease; SD, stable disease; Topo, topotecan; TMZ, temozolomide; VXL, vincristine/dexamethasone/L‐asparaginase. Source data are available online for this figure.

Objective responses using the Pediatric Preclinical Testing Consortium (PTCC) criteria (Houghton *et al*, 2007) were demonstrated in 10 of 16 non‐CNS solid tumor and leukemia models (Fig 3A). This included responses to chemotherapy in six, targeted monotherapy in four, and combination therapy in six patients (Fig 3A–K; Appendix Table S6). Drugs resulting in significantly prolonged survival (median event‐free survival (EFS) of treatment group at least twice longer than control and *P* < 0.001) were seen for three targeted agents in one of the three CNS models (RA‐002) (Fig 3L). No activity was observed for gemcitabine monotherapy in the group 3/MYC‐amplified medulloblastoma model (RA‐021) (Appendix Table S6). This is consistent with other studies reporting the lack of efficacy of gemcitabine as a single agent but synergies with other drugs such as pemetrexed (folate pathway inhibitor) and prexasertib (checkpoint kinase 1/2 (CHK1/2) inhibitor) (Morfouace *et al*, 2014; Endersby *et al*, 2021). Of the 22 treatments suggested by prior molecular testing, 8 led to an objective response (Table 2). As negative controls, targeted agents were included for seven PDXs where no direct molecular aberrations for these agents were identified, and no objective responses were seen. The additional benefit of combination treatment could be assessed in 12 models. An improved objective response was observed for drug combinations compared to monotherapy for four patients [RA‐001 (Fig 3F), RA‐012 (Fig 3G), RA‐039 (Fig 3J), and RA‐049 (Fig 3E)]. Furthermore, PDX could have facilitated prioritization of different treatment options in eight patients for whom PDX identified both effective and non‐effective treatments. Examples include an anaplastic large cell lymphoma PDX (RA‐049) (Fig 3E) demonstrating no response to brentuximab but complete response (CR) to ceritinib and alectinib, and a T‐cell acute lymphoblastic leukemia (T‐ALL) PDX (RA‐045) (Fig 3B) with no response to venetoclax but complete response to nelarabine and carfilzomib/chemotherapy.

Together, PDX modeling for 19 patients confirmed drug sensitivities seen in prior HTS or molecular analyses in five patients, identified new treatment options which were not informed by HTS or molecular analyses in seven patients, and provided useful negative results for treatment prioritization in 17 patients for whom alternative treatments or other effective options identified by PDX could be considered.

### Overall clinical impact of a four‐part diagnostic platform

**Figure 4:**
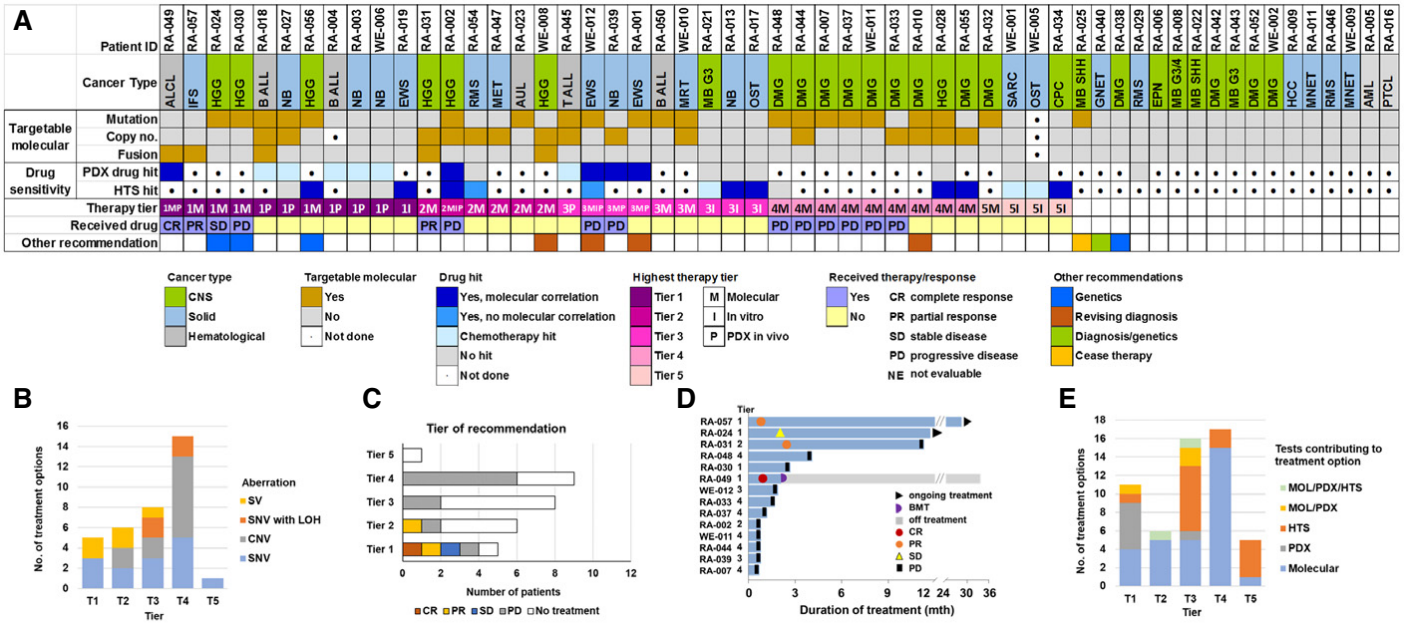
Individualized therapeutic options in 56 pediatric high‐risk cancers AOverview of each patient’s precision oncology platform results. The highest tier of therapy options for each patient is shown. A total of 55 recommendations were made in 39 patients.BTier of therapy and related molecular alterations. Structural variant (SV), single‐nucleotide variant (SNV) with loss of heterozygosity (LOH) in a tumor suppressor gene, copy number variant (CNV).C, DTreatment response by therapy tier. Fourteen of 29 patients with molecular‐based therapeutic options received the treatment.ETests contributing to the identification of treatment by tier.

We then evaluated whether our four‐part testing platform with molecular (DNA and RNA), HTS, and PDX would increase treatment options for the overall group of 56 high‐risk pediatric cancer patients, compared to molecular alone. Of the 56 patients, 32 had only molecular analyses, 23 had molecular, HTS and/or PDX drug testing conducted, and one patient had only HTS and PDX testing with no molecular analysis performed (Appendix Table S3). We used five tiers of therapy evidence as described in the Individualized Cancer Therapy (iCAT) study (Harris *et al*, 2016) for molecular, HTS or PDX drug sensitivity. The overall rate of identification of treatment options was high, with 55 treatment options identified for 70% of patients across the testing platform (Fig 4A; Table EV2).

**Figure EV1:**
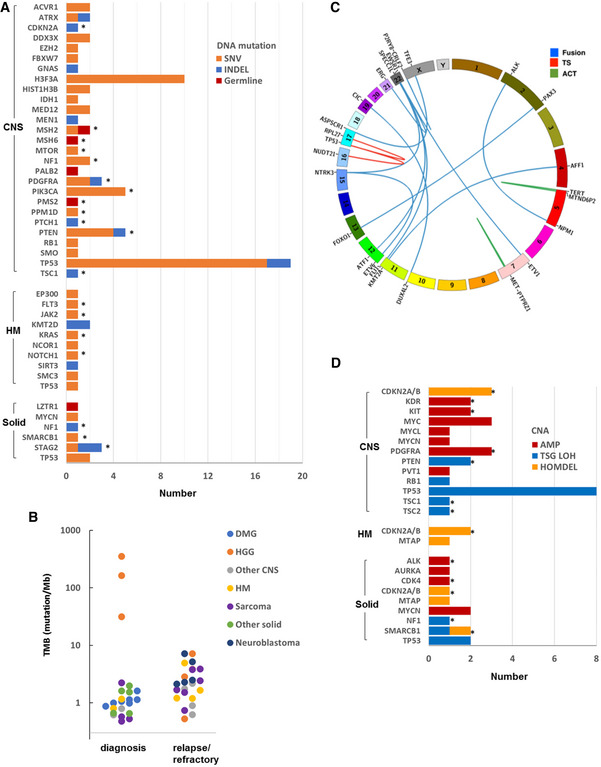
Molecular aberrations in 55 pediatric high‐risk cancers Genes with somatic and germline DNA mutations (single‐nucleotide variant (SNV) and indel) considered to be pathogenic or likely pathogenic by whole genome sequencing (WGS) and/or panel sequencing. Thirty of 55 samples were found to have 1 or more pathogenic or likely pathogenic mutations. The cohort consists of 27 central nervous system (CNS) tumors, 8 hematologic malignancies (HMs), and 20 non‐CNS solid tumors. Targetable aberrations are indicated by asterisks.Tumor mutation burden (TMB) derived from WGS in 23 samples obtained at diagnosis and 24 samples at refractory/relapse.Structural variants (SVs) detected by WGS and/or RNA‐seq in 55 samples. Seventeen reportable SVs included 13 fusions, 2 oncogenic activating (ACT) SVs, and 2 tumor suppressor (TS) loss‐of‐function SV.Reportable copy number variations (CNVs) included amplifications (≥ 6 copies), loss of heterozygosity (LOH) associated with a loss‐of‐function mutation in a tumor suppressor gene (TSG LOH) and homozygous deletion (HOMDEL) of TSG. Twenty‐four samples were found to have 1 or more reportable CNV. Targetable aberrations are indicated by asterisks.

Molecular profiling was performed in 55 patients. The results of 47 patients have been described in conjunction with the follow‐up national trial (Wong *et al*, 2020). The key molecular aberrations of this cohort are provided in Fig EV1A–D; Tables EV2 and EV3). DNA and RNA profiling provided therapeutic options for 29 of 55 patients (Fig 4A; Table EV2). This included targetable fusions (*n* = 4) and CNV (*n* = 7) in nine patients among whom no targetable DNA mutations were present (Fig 4A and B). Five patients also had reportable germline mutation. Fourteen of 55 patients received the personalized treatment, with a clinical benefit rate in 4 (1 complete response (CR), 2 partial responses (PRs), 1 stable disease (SD)) (Fig 4C and D; Table 3). When we correlated the clinical response with the prediction of response by either molecular, HTS or PDX (Table 3), we found 4/14 molecular, 4/5 HTS, and 4/8 PDX predictions correctly forecast a response in the patient receiving that specific drug. This included the prediction of response or non‐response and strongly supports the clinical relevance of HTS and PDX testing.

**Table 3:** Prediction of patient treatment response by molecular, high‐throughput drug screening (HTS) or patient‐derived xenograft (PDX).

Patient ID	Diagnosis	Treatment received	Molecular target	Tier	Clinical outcome	Molecular predicted patient drug response	HTS predicted patient drug response ^a^	PDX predicted patient drug response ^a^
Molecular‐guided treatment								
RA‐049	ALCL	Ceritinib	NPM1 ‐ ALK	1M	CR	Yes	−	Yes
RA‐057	IFS	Larotrectinib	SPECC1L ‐ NTRK3	1M	PR	Yes	−	−
RA‐031	HGG	Larotrectinib	ETV6 ‐ NTRK3	2M	PR	Yes	−	−
RA‐024	HGG	Nivolumab	Hypermutation	1M	SD	Yes	−	−
RA‐048	DMG	Sirolimus	PIK3CA mut	4M	PD	No	Yes	−
RA‐002	HGG	Sirolimus	TSC1 mut/LOH	2M	PD	No	No	No
RA‐039	NBL	Ceritinib	ALK amplification	3M	PD	No	−	No
WE‐012	EWS	Olaparib + irinotecan	STAG2 and TP53 mut	3M	PD	No	−	−
RA‐030	HGG	Nivolumab	Hypermutation	1M	PD	No	−	−
RA‐007	DMG	Sirolimus	mTOR mutation	4M	PD	No	−	−
RA‐033	DMG	Dasatinib	PDGFRA amp	4M	PD	No	−	−
RA‐037	DMG	Sirolimus	PIK3CA mut	4M	PD	No	−	−
RA‐044	DMG	Sirolimus + dasatinib	PTEN mut/LOH PDGFRA/KIT amp	4M	PD	No	−	−
WE‐011	DMG	Sirolimus	PIK3CA mut	4M	PD	No	−	−
Other treatment								
RA‐002	HGG	TMZ	NA	NA	PD	NA	Yes	Yes
RA‐027	NBL	Cyclo/Topo IRN/TMZ	NA	NA	PD PD	NA	Yes Yes	No No
RA‐039	NBL	Cyclo/Topo	NA	NA	Mixed	NA	–	Yes
RA‐045	T‐ALL	Carfilzomib + VXL	NA	NA	CR	NA	–	Yes

ALCL, anaplastic large cell lymphoma; amp, amplification; CR, complete response; Cyclo/Topo, cyclophosphamide/topotecan; DMG, diffuse midline glioma H3 K27M mutant; EWS, Ewing’s sarcoma; HGG, high grade glioma; IFS, infantile fibrosarcoma; IRN, irinotecan; LOH, loss of heterozygosity; MCR, maintained complete response; mut, mutation; NA, not applicable; NBL, neuroblastoma; PD, progressive disease; PR, partial response; SD, stable disease; T‐ALL, T‐cell acute lymphoblastic lymphoma; TMZ, temozolomide; VXL, vincristine/ dexamethasone/L‐asparaginase.

^a^ “–” denotes high‐throughput drug screening (HTS) or PDX not performed.

Twenty‐four patients had HTS or PDX testing performed. HTS and PDX testing identified 71 treatment options in 22 of the 24 patients. A total of 20 of these 71 treatment options were solely derived from HTS or PDX findings, while 30 treatment options were solely identified by molecular analyses. Furthermore, 11 of these 24 patients had no targetable molecular lesions, whereas HTS and PDX testing uncovered new treatment options for 10 of these 11 patients. Compellingly, of the 17 Tier 1 (higher level evidence) treatment recommendations, 6 were derived from HTS or PDX findings alone (Fig 4E). HTS and PDX could also provide informative negative results. Despite a molecular result suggesting a treatment, accompanying HTS and PDX on the same patient would have correctly not supported the use of the drugs in 10 of the 24 patients (Appendix Table S8).

Together these data showed that each of the four components of the diagnostic platform could provide either the only evidence for a treatment option, or orthogonal proof of a targetable driver gene or pathway suggested by another component of the platform. Overall, 39 of the 56 patients would have received at least one therapy option and most options were derived from one testing platform. Forty‐two of the 56 patients would have received new clinical information (32 therapeutic only; 4 therapeutic & diagnosis; 3 therapeutic & germline; 1 diagnosis & germline; 1 germline; 1 cease (Complete Elimination of Autistic Specrum Expression) therapy) from the four‐component diagnostic platform which could have changed their clinical management (Fig 4A).

## Discussion

We hypothesized that diagnostic strategies, in addition to genomics, aimed at better matching drug to target for an individual patient would increase therapeutic options, and potentially provide orthogonal proof that a particular target–drug interaction had higher likelihood of being clinically effective. Surprisingly, we found that HTS and PDX, each contributed to a higher proportion of patients for whom a therapeutic option could be generated, when compared with genomics‐alone studies (Harris *et al*, 2016; Parsons *et al*, 2016; Pincez *et al*, 2017). Of the HTS‐ or PDX‐related treatment options, 20% provided orthogonal confirmation that a particular drug suggested by molecular analyses should be trialed, whereas 80% were not suggested by prior genomic analyses. Our study indicates that genomics, transcriptomics, HTS and PDX testing in a combined platform will provide high‐risk pediatric cancer patients with a greater chance of therapeutic options.

While HTS utilizing patient‐derived tumor cells is widely published, very few have been conducted within the context of a prospective clinical study. Three seminal studies on adult cancer have described the integration of *in vitro* screening in a precision oncology trial (Pemovska *et al*, 2013; Pauli *et al*, 2017; Snijder *et al*, 2017). Our study represents the first pediatric study integrating *in vitro* and *in vivo* drug screening into a clinical study. Important features of our work include the rapid authentication and validation of cultures, implementation of a custom‐designed HTS drug library, and our adoption of a z‐score outlier approach similar to one adult carcinoma study (Pauli *et al*, 2017).

In our study, strong HTS or PDX drug sensitivity results for particular signaling pathways were not always supported by orthogonal genomic aberrations, indicating the presence of other cancer driver processes. The phenomenon of cancer vulnerabilities and synthetic lethality (Nijman & Friend, 2013; Ashworth & Lord, 2018) has led to the creation of cancer dependency maps using genome‐scale RNA interference (RNAi) screens on cancer cell lines, which have shown that the majority of vulnerabilities were gene expression‐based (Tsherniak *et al*, 2017). Together, this feature of our study suggests that aberrant expression of dependency genes, driven by processes other than structural genomic events or mutations, such as those of the epigenome and proteome, can confer drug sensitivity or resistance.

Another exciting observation from our study was the relatively high PDX engraftment rate, and the capacity for these PDX‐derived samples to proceed to HTS within a clinically meaningful time frame. It was anticipated that many tumor types would fail *in vivo* engraftment and therefore not contribute to a patient’s therapeutic options. Instead, we observed that PDX models made a significant contribution to therapeutic options, and the success of this study. Our results in solid tumors compare favorably to a study from St. Jude Children’s Research Hospital with an orthotopic PDX establishment rate of 45% from 148 pediatric solid tumors (Stewart *et al*, 2017). However, our CNS orthotopic PDX engraftment rate of 20% is lower than that described in the literature, with some studies reporting a success from 30 to 56% (Brabetz *et al*, 2018; Smith *et al*, 2020; He *et al*, 2021). This is likely related to inoculating *in vitro* expanded tumor cells in our study versus direct implantation of tumor cells in other studies, and such a difference in success has been described in diffuse midline glioma (Tsoli *et al*, 2019). We adopted the former approach to allow use of *in vitro* expanded primary cells from small brain tumor biopsies for both HTS and PDX. Nonetheless, an overall increase in the number of pediatric oncology PDX models will expand the platform on which future drug discoveries can be tested.

This study represents the first pediatric precision oncology study which has integrated genomics, transcriptomics, *in vitro* and *in vivo* drug efficacy testing to derive therapeutic options for high‐risk pediatric cancer patients. We have demonstrated that this comprehensive approach is feasible, has the potential to expand therapeutic options for children with high‐risk cancer and improve clinical outcomes. However, significant challenges remain for translating preclinical drug testing results into the clinic, such as the correlations between clinical responses, animal and human pharmacokinetics, and *in vitro* and *in vivo* sensitivity signals.

## Materials and Methods

### Patients and samples

The study was approved by the Sydney Children’s Hospitals Network Human Research Ethics Committee (LNR/14/SCH/497). Informed consent was obtained from all participants or their guardian. Patients aged < 21 years with suspected/confirmed diagnosis of high‐risk malignancy (expected probability of survival < 30%) could be consented and registered on the study. Patients > 21 years with high‐risk pediatric type cancers could be registered with study chair approval. Registration encouraged fresh tissue to be submitted for processing prior to confirmation of a high‐risk cancer diagnosis. A patient was eligible for enrollment with a high‐risk cancer diagnosis, submitted tissue that contained adequate malignant cells and germline sample available. Adoption of the identified treatment option was at the discretion of the treating oncologist. All experiments conformed to the principles set out in the World Medical Association (WMA) Declaration of Helsinki and the Department of Health and Human Services Belmont Report.

### Patient‐derived tumor cell culture

Non‐CNS primary tumors were dissociated using a human tumor dissociation kit (Miltenyi Biotec) with the gentleMACS Octo Dissociator (Miltenyi Biotec). Neuroblastoma cells were cultured in Iscove’s Modified Dulbecco’s Medium (IMDM) (Life Technologies) supplemented with 20% fetal bovine serum (FBS) and 1x ITS (insulin–transferrin–selenium) (Life Technologies). Other extracranial tumor cells were cultured in alpha‐minimal essential medium (alpha‐MEM) supplemented with 10% FBS, 1x ITS (Life Technologies), and Rock Inhibitor (Y‐27632 HCl Selleck Chemicals). Primary CNS tumors were dissociated, as previously described (Lin & Monje, 2017). Dissociated CNS cells were grown in stem cell media consisting of Dulbecco’s Modified Eagle Medium/Nutrient Mixture F‐12 (DMEM/F12) and Neurobasal–A media (1:1, Life Technologies) supplemented with glutamax, pyruvate, non‐essential amino acids, HEPES (4‐(2‐hydroxyethyl)‐1‐piperazineethanesulfonic acid) buffer, antibiotic/antimycotic (Invitrogen), heparin (Stemcell Technologies), and growth factors (basic fibroblast growth factor (bFGF) and epidermal growth factor (EGF), Stemcell Technologies). Diffuse midline gliomas also received platelet‐derived growth factor AA (PDGF‐AA) and platelet‐derived growth factor BB (PDGF‐BB) (Stemcell Technologies). A solution of 1x antibiotic–antimycotic (Thermo Fisher) was added to all patient‐derived cell cultures. Long‐term primary CNS cultures were tested for mycoplasma using the MycoAlert kit (Lonza) and were mycoplasma free. Primary cultures for non‐CNS tumors were short‐term cultures and were not tested for mycoplasma.

Non‐CNS solid tumor PDX cells were also subjected to *in vitro* expansion for high‐throughput drug screen. PDX cells were plated in Corning^®^ Costar^®^ Ultra‐Low attachment 96‐well plates at 4,000 cells/well to establish 3D spheroid cultures. CellTiter‐Glo^®^ 3D Cell Viability Assay was conducted at day 3 and day 7 to evaluate proliferation. *In vitro* expansion of primary leukemia cells was not performed.

### Authentication of patient cells undergoing HTS

Confirmation that cells used in HTS were from the correct patient was obtained by short tandem repeat (STR)/microsatellite DNA profiling at the Garvan Institute of Medical Research (Australia) using the PowerPlex^®^ 18D system with 18 markers. The microsatellite profiles were compared between the original primary sample and the cells subjected to HTS. As microsatellite profiles could vary while tumor cells underwent *in vitro* expansion or *in vivo* engraftment, cells were considered matching the primary sample with > 80% identity. Cultures failing STR authentication or without STR authentication performed were not used for HTS analysis.

### Validation of tumor cell content in cells undergoing HTS

Histopathology, flow cytometry, or SNP array was used to establish whether cells subjected to HTS were representative of the original tumor type. For histopathology assessment, tumor cell block was prepared using the plasma–thrombin method before being fixed in formalin (Xie *et al*, 2021). Flow cytometry was used to detect tumor marker expression. Samples were acquired by the BD FACSCanto™ II system (BD Biosciences, New Jersey, USA) and data analyzed by FlowJo software (BD Biosciences, New Jersey, USA). The gating strategy to define positive population was designed to only include 0.1% cells in the isotype negative control samples. The following antibodies were used for flow cytometry: PE mouse anti‐human CD99 (Cat no. 130‐104‐315) (Miltenyi Biotec, Bergisch Gladbach, Germany) (Ewing’s sarcoma (EWS)), R‐phycoerythrin (PE) mouse anti‐human GD2 (Cat no. 562100) (neuroblastoma and osteosarcoma), fluorescein isothiocyanate (FITC) mouse anti‐human CD56 (Cat no. 562794) (rhabdomyosarcoma), and anti‐allophycocyanin (APC) mouse anti‐human CD45 (Cat no. 555485) (lymphoma and leukemia). The latter three antibodies were purchased from Becton Dickinson (BD) Biosciences (New Jersey, USA). All antibodies were used at a concentration of 1:20 dilution.

An Illumina Infinium Global Screening Array‐24 v2.0, which contains a total of 665,608 markers, was used to assess tumor cell proportions using the CNV profile. Fluorescent signals were imported into the BioDiscovery NxClinical^TM^ software and normalized fluorescent signal intensities were compared with the signal intensities of a set of reference genotypes created from over 300 samples generated on the same platform. The log2‐ratios between sample and reference signals were calculated for each SNP. LogR and B Allele Frequency (BAF) datasets were imported into ASCAT2 (Van Loo *et al*, 2010), an R program for tumor cell % estimation. CNV profile was compared to the CNV profile of the original tumor from whole genome sequencing (WGS) data. Cultures found to contain < 80% of tumor cells were not used for HTS.

### High‐throughput drug screening

The *in vitro* drug screening library was composed of 111 compounds (Table EV1) which were sourced from commercial vendors such as MedChem Express and Selleck and prepared as 100% dimethyl sulfoxide (DMSO) solutions. Long‐term storage of the library is housed at Compounds Australia, Griffith University (Australia). HTS was performed at the ACRF Drug Discovery Centre at Children’s Cancer Institute (Australia). Source of tumor cells included dissociated cells from patient samples, dissociated cells from PDX, or cells expanded through short‐term *in vitro* culture. Cells were seeded in 384‐well assay plates as single‐cell suspensions using a Multidrop Combi dispenser (Thermo Scientific), at a density of 1,000 cells/well for CNS tumors and 2,000 cells/well for non‐CNS tumors. Rock Inhibitor (Y‐27632 HCl Selleckchem) was also added, except for CNS tumors and neuroblastoma. Ultra‐low attachment plates (PrimeSurface^®^ 384U White Plate, Sumitomo Bakelite Co., Ltd, Japan) were used for the non‐CNS non‐neuroblastoma samples. Plates were incubated at 37°C, 5% CO_2_, and 20% oxygen (CNS and neuroblastoma) or 5% oxygen (other tumors) in a humidified environment. Following incubation for 72 h, cells were treated with test compounds using a Hamilton STAR liquid handling robot equipped with a pintool dispensing device. Cells were treated in duplicate in five different concentrations (10‐fold dilutions; 0.5–5,000 nM). After 72 h drug exposure, cell viability was measured using the CellTiter‐Glo luminescent assay (Promega) according to the manufacturer’s instructions with a PerkinElmer EnSpire multimode plate reader.

All data analysis was performed using the ActivityBase (IDBS) software suite. The raw test data were normalized to negative control (DMSO only) and positive control wells (10 μM thonzonium bromide for 100% kill) for the calculation of percent survival for each data point. Dose–response curves were fitted using a four‐parameter logistic function with the top asymptote of the curve being fixed to 100% survival and the bottom asymptote allowed to vary between 0% and 75% survival. IC_50_ concentrations and area under the dose–response curve (AUC) were calculated using the curve fitting parameters, with IC_50_ being defined as the drug concentration resulting in 50% cell survival.

### *In* *vitro* drug sensitivity analysis

An in‐house database of *in vitro* IC_50_ and AUC values was developed to identify significant candidate drug hits. A total of 49 samples are in the database which consisted of 9 neuroblastoma cell lines, 5 CNS cell lines, 15 solid tumors, 7 neuroblastoma, 11 CNS tumors, and 2 hematologic malignancies (HMs). All 111‐compound sensitivity results for each patient were compared against the other results in the database using z‐score methodology, where the mean and variance of each compound in the database was compared against the corresponding AUC and IC_50_ value of the patients. A drug was considered a hit if the z score of the AUC and IC_50_ was ≤ −2 (i.e., the patient’s AUC and IC_50_ value was 2 standard deviations lower than the cohort mean). A drug hit was then further classified into either Level 1 or 2 to predict clinical translation, by taking into consideration the plasma drug concentration achievable in humans. A Level 1 hit met z‐score criteria for both AUC and IC_50_, and the IC_50_ < Cmax (peak plasma concentration) or IC_50_ < Css (steady‐state plasma concentration). A Level 2 hit met z‐score criteria for AUC and IC_50_ only.

### Patient‐derived xenografting and *in vivo* drug efficacy

All PDX studies complied with ethical regulations and were approved by the University of New South Wales Animal Ethics Committee. PDXs were established, as previously described (Liem *et al*, 2004; Morton & Houghton, 2007; Tsoli *et al*, 2019; Kamili *et al*, 2020). Six‐ to 8‐week‐old, female, non‐obese diabetic/severe combined immunodeficiency/interleukin 2 (NOD/SCID/IL‐2) receptor gamma^−/−^ (NOD. Cg‐*Prkdc^scid^ Il2rg^tm1Wjl^*/SzJAusb; NSG) mice were purchased from Australian BioResources (Moss Vale, NSW, Australia) for non‐CNS models and from Animal Resources Centre (Canning Vale, WA, Australia) for CNS models. Upon arrival, the animals were housed in translucent polycarbonate autoclavable cages (22 cm W × 15 cm H × 30 cm L, Tecniplast, Italy) with air filters in positive pressure ventiracks. Bedding, enviro dry, and igloos were provided for environmental enrichment. Irradiated rat and mouse breeder cubes and water were provided *ad libitum*. Non‐CNS solid PDXs were expanded in secondary cohorts of mice by subcutaneous inoculation of PDX cells in NSG mice. Two to twelve animals were included for each treatment, depending on the growth rate of individual PDX and the number of cells available following secondary expansion. Allocation of treatment was not randomized and investigators were not blinded to treatment allocation. Treatment was commenced when a tumor reached 100 mm^3^ in size, as measured by vernier calipers using the formula: volume = L × W × H/2. An event was defined as a quadrupling of tumor volume from the start of treatment. *In vivo* drug activity in non‐CNS solid and leukemia was evaluated by objective response categorized as MCR (maintained complete response), CR (complete response), PR (partial response), SD (stable disease), PD1 (progressive disease 1), and PD2 (progressive disease 2), as previously described (Houghton *et al*, 2007). In CNS orthotopic models, the PDX was expanded in a secondary cohort by injecting stereotactically PDX cells in the corresponding anatomical location in NOD/SCID mice. Ten animals were included for each treatment. Drug treatments were commenced approximately 30 days after intracranial injection. An event was defined as weight loss ≥ 20% or severe neurological decline when the animal was euthanized according to definitions and procedures set by the UNSW Animal Ethics Committee. The presence of tumor was confirmed by histology. Event‐free survival (EFS) was defined as time of intracranial injection to event. EFS T/C value was defined by the ratio of the median time to event within the treatment group (T) and the median time to event of the respective control group (C). Drugs were considered to be active if there was a significant difference between drug‐treated and control mice in EFS (*P* ≤ 0.05) and EFS T/C greater than 2.

### Molecular profiling and computational analysis

#### Whole genome sequencing and whole transcriptome data analysis

WGS was conducted at the Garvan Institute of Medical Research (Australia) and whole transcriptome sequencing at the Murdoch Children’s Research Institute (Australia), details of which have been previously described (Wong *et al*, 2020).

#### Capture panel data analysis

Targeted panel sequencing was conducted at the Peter MacCallum Cancer Centre (Australia) using the comprehensive cancer panel CCP v.1 (625 genes) or CCP v.2 (386 genes) on DNA isolated from tumor and a matched germline sample. Genes are listed in Table EV4. KAPA Hyper (Roche, Pleasanton, CA) libraries were prepared from 100 to 200 ng of sonically sheared genomic DNA according to the manufacturer’s instructions. Library members representing targets of interest were enriched using SureSelect^XT^ (Agilent, Santa Clara, CA) hybridization according to the manufacturer’s instructions. Pooled enriched libraries were sequenced at 500× mean coverage for tumor and 200× for germline on a NextSeq 500 sequencer (Illumina, San Diego, CA) using paired 75bp reads. Alignment and variant calling were performed using Seqliner pipeline (v0.7; seqliner.org). Reads were aligned to GRCh37/hg19 using BWA‐MEM (v0.7.10). Picard (v1.119) was employed to sort and index the alignment BAM files, and to mark duplicate reads. Genome Analysis Toolkit (GATK; v3.2) performed local realignment around indels and was used to recalibrate base quality scores. Quality control (QC) was visualized with FastQC (https://www.bioinformatics.babraham.ac.uk/projects/fastqc/) and MultiQC (Ewels *et al*, 2016). Somatic variants were called using VarDict (v1.4.6) and MuTect2 (v3.5) (Cibulskis *et al*, 2013). Germline variants called on GATK HaplotypeCaller (v3.2) (https://software.broadinstitute.org/gatk/) were subtracted. Variant annotation, classification, and reporting was performed using PathOS v1.5.3 (Doig *et al*, 2017). Tumor content copy number aberrations and ploidy estimates were detected using FACETs (v0.5.6) and CNspector (Markham *et al*, 2019). Structural variants were detected using GRIDSS (v0.11.6) (Cameron *et al*, 2017).

### Statistical analyses

No statistical methods were used to predetermine sample size. Statistical analyses for z‐score in HTS were performed using R (v3.5.3) (https://www.R‐project.org/). Statistical analyses for *in vivo* studies were performed using PRISM (version 8, GraphPad, Inc.) and differences were considered significant when *P* was < 0.05.

## Author contributions

GMM, MH, DSZ, TNT, MDN, and LMSL conceived and designed the study. VT managed and coordinated the operation of the study. LMSL collected and reviewed all clinical data. GBM and LD‐P contributed to patient samples and clinical annotation. PGE oversaw the variant curation team. MJC oversaw the bioinformatics and genomics analysis. MJC and MW developed genomic analysis, curation and integration pipelines. CM performed the bioinformatics analysis of RNA‐seq and HTS. AM performed the bioinformatics analysis of panel sequencing and SNP array. PB, AKu, EVAM, DK, CW, MJC, MP, and PGE curated the sequencing data. PB and CM collated the molecular data. DMT provided expert input into precision medicine. AF and SBF provided expert input into the conduct of molecular curation, panel sequencing and analysis. KMT, MP, and MW provided expert input into the interpretation of germline variants. KMT, MW, and MP provided expert input into the interpretation of germline variants. RBL and KLM oversaw the preclinical work. GMM, DSZ, TNT, and LMSL designed the HTS library. RBL provided expert input into leukemia preclinical models. JIF and TNT provided expert input into neuroblastoma preclinical models. DSZ provided expert input into CNS preclinical models. JX, AKa, and MT oversaw the development and optimization of primary tumor cell culture and PDX establishment. PT and SJ established CNS primary culture/PDX. GE, MS, and DB established extracranial primary culture/PDX. PT, SJ, LTM, DB, GE, SA, AG, AKh, MS, JYL, and RC conducted and analyzed *in vivo* drug efficacy experiments. GE, MS, CU, SJ, DB, PT, JYL, RC, DGW, PAS, and EM processed patient samples, cell cultures, and PDX. TWF, S‐OC, and GMA optimized, conducted, and analyzed the HTS. JX, AKa, and MT designed, conducted, and analyzed the PDX drug treatment experiments. AG processed patient samples and conducted histopathology evaluation of primary cell cultures and xenografts. JAB and FS contributed to the immunohistochemical analyses. GMM, DSZ, TNT, D‐AK‐Q, and LMSL provided clinical expertise for therapeutic recommendations. LMSL integrated and analyzed all data. LMSL, PGE, DSZ, and GMM wrote the manuscript. All authors reviewed and approved the manuscript.

## Conflict of interest

The authors declare that they have no conflict of interest.

## Supporting information



Appendix

Expanded View Figures PDF

Table EV1

Table EV2

Table EV3

Table EV4

Source Data Figure 2AB

Source Data Figure 3AL

## Data Availability

Sequencing data (EGAD00001008358) are deposited at the European Genome‐phenome Archive (https://ega‐archive.org/studies/EGAS00001004572) (Wong *et al*, 2020). Corresponding patient and sample ID numbers for this dataset can be found in Table EV3.
