# Supplementary material for: In vitro and in vivo drug screens of tumor cells identify novel therapies for high‐risk child cancer
Source: EMBO Mol Med. 2021 Dec 20;14(4):e14608. doi: 10.15252/emmm.202114608 (PMC8988207; doi:10.15252/emmm.202114608)
Supplement: Supplementary file 1 — Appendix [file EMMM-14-e14608-s004.pdf]

APPENDIX

In vitro and in vivo drug screens of tumor cells identify novel therapies for high-risk child cancer

Loretta M S Lau[1,2,3], Chelsea Mayoh[1,2], Jinhan Xie[1], Paulette Barahona[1], Karen L. MacKenzie[1], Marie Wong[1,2], Alvin Kamili[1,2], Maria Tsoli[1], Tim W. Failes[1,4], Amit Kumar[1,5], Emily V A Mould[1], Andrew Gifford[1,2], Shu-Oi Chow[1,4], Mark Pinese[1,2], Jamie I Fletcher[1,2], Greg M. Arndt[1,4], Dong-Anh Khuong-Quang[5,6], Carol Wadham[1], Georgina Eden[1], Peter Trebilcock[1], Swapna Joshi[1], Stephanie Alfred[1], Anjana Gopalakrishnan[1], Aaminah Khan[1], Dylan Grebert Wade[1], Patrick A. Strong[1], Elodie Manouvrier[1], Lisa T Morgan[1] Roxanne Cadiz[1], Caitlin Ung[1], David M Thomas[7,8], Katherine M Tucker[9,10], Meera Warby[9], Geoffrey B McCowage[11], Luciano Dalla-Pozza[11], Jennifer A Byrne[12,13], Federica Saletta[1], Andrew Fellowes[14], Stephen B Fox[14,15], Murray D Norris[1,2,16], Vanessa Tyrrell[1], Toby N Trahair[1,2,3], Richard B Lock[1,2], Mark J Cowley[1,2,17], Paul G Ekert[1,2,13], Michelle Haber[1,2], David S Ziegler[*,†,1,2,3], Glenn M Marshall[*,†,1,2,3]

Table of content:

Appendix Table S1. Patient demographics and sample details.

Appendix Table S2. Patient-derived xenograft establishment.

Appendix Table S3. Tests conducted in the 56 samples.

Appendix Table S4. HTS samples.

Appendix Table S5. High throughput drug screen in 17 samples.

Appendix Table S6. PDX drug treatment response in 19 models.

Appendix Table S7. Dosing and schedule for PDX drug efficacy studies.

Appendix Table S8. Molecular aberrations which did not correlate with drug responses in HTS and PDX.

Appendix Table S1. Patient demographics and sample details

	No. of patients			Age (y)		Disease status [a]			Tissue	
**Diagnosis**	**Total**	**Male**	**Female**	**Median**	**Range**	**Diagnosis**	**Relapse/ refractory**	**Post treatment**	**Fresh**	**Frozen**
All patients	56	28	28	7.0	0.6-25	24	28	4	46	10
CNS tumors	27	13	14	7.8	0.6-25	14	11	2	22	5
DMG	13	6	7	7.0	4-14	9	2	2	9	4
HGG	7	2	5	10.6	3-25	3	4	0	7	0
Medulloblastoma	5	4	1	9.6	1-19	2	3	0	5	0
Other CNS tumor	2	1	1	8.4	0.6-16	0	2	0	1	1
Solid tumors	21	10	11	5.9	1-22	8	11	2	17	4
Sarcoma [c]	7	3	4	13.3	3-19	1	5	1	7	0
Other solid	9	4	5	5.5	1-22	7	1	1	5	4
Neuroblastoma	5	3	2	5.4	1-7	0	5	0	5	0
Hematological	8	5	3	6.8	1-15	2	6	0	7	1
Leukemia	6	3	3	6.0	1-15	1	5	0	6	0
Lymphoma	2	2	0	13.6	12-15	1	1	0	1	1

[a] Disease status when sample was obtained. Post treatment: patient with initial high-risk disease who provided sample following upfront radiotherapy or chemotherapy

[b] Includes Ewing sarcoma, rhabdomyosarcoma, osteosarcoma

Abbreviation: DMG, diffuse midline glioma H3 K27M mutant; HGG, high grade glioma

Appendix Table S2. Patient-derived xenograft establishment

	Fresh sample	PDX model	PDX attempted	PDX successful		Engraftment time (mth)	
	N		N	N	%	Median	Range
All	46		42	22	52%	3.2	1.0 – 10.9
CNS	22	orthotopic	20	4	20%	2.7	1.7 – 4.1 *
Other solid	12	subcutaneous	12	9	75%	4.1	1.6 – 10.9
NB	5	subcutaneous	5	5	100%	4.5	1.6 – 7.1
Leukemia	6	orthotopic	4	3	75%	1.1	1.0 – 3.7
Lymphoma	1	subcutaneous	1	1	100%	3.7	NA

* includes time for ex vivo expansion of primary CNS tumour cells prior to inoculation when required

Appendix Table S3. Tests conducted in the 56 samples.

Patient ID	Category	Diagnosis	Sequenced	WGS	RNAseq	HTS	PDX drug testing
WE-005	Solid	Osteosarcoma	No	No	No	Yes	Yes
RA-002	CNS	High grade glioma	Yes	Yes	Yes	Yes	Yes
RA-028	CNS	High grade glioma	Yes	Yes	Yes	Yes	Yes
RA-021	CNS	Medulloblastoma	Yes	Yes	Yes	Yes	Yes
RA-054	Solid	Alveolar rhabdomyosarcoma	Yes	Yes	Yes	Yes	Yes
WE-012	Solid	Ewing's sarcoma	Yes	Yes	Yes	Yes	Yes
RA-003	Solid	Neuroblastoma	Yes	Yes	Yes	Yes	Yes
RA-013	Solid	Neuroblastoma	Yes	Yes	Yes	Yes	Yes
RA-027	Solid	Neuroblastoma	Yes	Yes	Yes	Yes	Yes
WE-006	Solid	Neuroblastoma	Yes	Yes	Yes	Yes	Yes
RA-017	Solid	Osteosarcoma	Yes	Yes	Yes	Yes	Yes
WE-001	Solid	Undifferentiated sarcoma	Yes	Yes	Yes	Yes	Yes
RA-034	CNS	Choroid plexus carcinoma	Yes	Yes	Yes	Yes	No
RA-055	CNS	Diffuse midline glioma	Yes	Panel	No	Yes	No
RA-048	CNS	Diffuse midline glioma	Yes	Yes	Yes	Yes	No
RA-056	CNS	High grade glioma	Yes	Yes	Yes	Yes	No
RA-019	Solid	Ewing's sarcoma	Yes	Yes	Yes	Yes	No
RA-004	HM	Pre-B acute lymphoblastic leukemia	Yes	Yes	Yes	No	Yes
RA-018	HM	Pre-B acute lymphoblastic leukemia	Yes	Yes	Yes	No	Yes
RA-045	HM	T acute lymphoblastic leukemia	Yes	Yes	Yes	No	Yes
RA-029	Solid	Alveolar rhabdomyosarcoma	Yes	Yes	Yes	No	Yes
RA-049	HM	Anaplastic large cell lymphoma	Yes	Yes	Yes	No	Yes
RA-001	Solid	Ewing's sarcoma	Yes	Yes	Yes	No	Yes
RA-039	Solid	Neuroblastoma	Yes	Yes	Yes	No	Yes
RA-007	CNS	Diffuse midline glioma	Yes	Panel	No	No	No
RA-032	CNS	Diffuse midline glioma	Yes	Yes	Yes	No	No
RA-033	CNS	Diffuse midline glioma	Yes	Yes	Yes	No	No
WE-002	CNS	Diffuse midline glioma	Yes	Panel	Yes	No	No
RA-010	CNS	Diffuse midline glioma	Yes	Panel	Yes	No	No
RA-037	CNS	Diffuse midline glioma	Yes	Panel	Yes	No	No
RA-038	CNS	Diffuse midline glioma	Yes	Yes	Yes	No	No
RA-042	CNS	Diffuse midline glioma	Yes	Panel	No	No	No
RA-044	CNS	Diffuse midline glioma	Yes	Yes	Yes	No	No
RA-052	CNS	Diffuse midline glioma	Yes	Yes	Yes	No	No
WE-011	CNS	Diffuse midline glioma	Yes	Yes	Yes	No	No
RA-006	CNS	Ependymoma	Yes	Yes	Yes	No	No
WE-008	CNS	High grade glioma	Yes	Yes	Yes	No	No
RA-024	CNS	High grade glioma	Yes	Yes	Yes	No	No
RA-030	CNS	High grade glioma	Yes	Yes	Yes	No	No
RA-031	CNS	High grade glioma	Yes	Yes	Yes	No	No
RA-043	CNS	Medulloblastoma	Yes	Yes	Yes	No	No
RA-008	CNS	Medulloblastoma	Yes	Yes	Yes	No	No
RA-022	CNS	Medulloblastoma	Yes	Yes	Yes	No	No
RA-025	CNS	Medulloblastoma	Yes	Yes	Yes	No	No
RA-023	HM	Acute undifferentiated leukemia	Yes	Yes	Yes	No	No
RA-005	HM	Acute myeloid leukemia	Yes	Panel	No	No	No
RA-016	HM	Peripheral T-cell lymphoma	Yes	Yes	Yes	No	No
RA-050	HM	Pre-B ALL	Yes	Panel	No	No	No
RA-046	Solid	Alveolar soft part sarcoma	Yes	Yes	Yes	No	No
RA-040	Solid	Gastrointestinal neuroectodermal tumor	Yes	Yes	Yes	No	No
RA-009	Solid	Hepatocellular carcinoma	Yes	Yes	Yes	No	No
RA-057	Solid	Infantile fibrosarcoma	Yes	Yes	Yes	No	No
WE-010	Solid	Malignant rhabdoid tumour	Yes	Yes	Yes	No	No
RA-011	Solid	Malignant melanotic neuroectodermal tumor of infancy	Yes	Yes	Yes	No	No
WE-009	Solid	Malignant melanotic neuroectodermal tumor of infancy	Yes	Yes	Yes	No	No
RA-047	Solid	Myoepithelial tumour	Yes	Yes	Yes	No	No

Appendix Table S4. HTS samples.

	ID	Category	Diagnosis	STR	Tumour cell content validation	Validation method	Validation result	HTS cell source	Passage	Culture	Time from receiving sample to HTS completion (mth)
1	RA-003	Solid	Neuroblastoma	match	Y	Flow	>95%	Primary	P0	spheroid	0.2
2	RA-013	Solid	Neuroblastoma	match	Y	Flow	>95%	PDX	PDX, X1	spheroid	11.1
3	RA-027	Solid	Neuroblastoma	match	Y	Flow	>95%	PDX	PDX, X1	spheroid	2.6
4	WE-006	Solid	Neuroblastoma	match	Y	Flow	>95%	Primary	P0	spheroid	0.3
5	RA-019	Solid	Ewing sarcoma	match	Y	Histo	Tumour cells	PDX	PDX, X3	spheroid	21.4
6	WE-012	Solid	Ewing sarcoma	match	Y	Flow	>95%	Culture	P7	spheroid	4.3
7	RA-017	Solid	Osteosarcoma	match	Y	Histo	Tumour cells	PDX	PDX, X3	spheroid	13.4
8	WE-005	Solid	Osteosarcoma	match	Y	Histo	Tumour cells	PDX	PDX, X3	spheroid	13.2
9	RA-054	Solid	Rhabdomyosarcoma	match	Y	Flow	>95%	PDX	PDX, X1	spheroid	4.4
10	WE-001	Solid	Undifferentiated sarcoma	match	Y	Histo	Tumour cells	PDX	PDX, X2	spheroid	16.8
11	RA-034	CNS	Choroid plexus carcinoma	match	Not done	-	-	Culture	P7	monolayer	2.4
12	RA-048	CNS	DMG H3 K27M	match	Not done	-	-	Culture	P5	monolayer	5.7
13	RA-055	CNS	DMG H3 K27M	match	Y	SNP	100%	Culture	P2	neurosphere	2.1
14	RA-002	CNS	High grade glioma	match	Not done	-	-	Culture	P3	neurosphere	1.9
15	RA-028	CNS	High grade glioma	match	Y	SNP	100%	Primary	P0	neurosphere	1.1
16	RA-056	CNS	High grade glioma	match	Not done	-	-	Culture	P3	monolayer	9.4
17	RA-021	CNS	Medulloblastoma Gp3	match	Not done	-	-	Culture	P1	neurosphere	1.1

STR, short tandem repeat; Flow, flow cytometry; Histo, histology; DMG, diffuse midline glioma.

Appendix Table S5. High throughput drug screen in 17 samples

Patient ID	Diagnosis	Targetable SNV and CNV	RNA expression	Level 1 drug hits					Level 2 drug hits				
				All	Targeted agents		Chemotherapy		All	Targeted agents		Chemotherapy	
				N	N	Detail	N	Detail	N	N	Detail	N	Detail
RA-002	High grade glioma	TSC1 mutation (LOH)<br>BRAF copy gain	High BRAF	6	6	sirolimus*, everolimus*, temsirolimus*, sorafenib†, vandetanib, lapatinib	0		1	1	axitinib	0	
RA-028	High grade glioma	PDGFRA mutation<br>CDKN2A/B biallelic loss	High PDGFRA	2	2	ponatinib*, lapatinib	0		1	1	crenolanib*	0	
RA-056	High grade glioma	ultra-hypermutation<br>PDGFRA mutation	nil	2	2	Pazopanib*, dasatinib*	0		4	3	pinometostat, fulvestrant, abiraterone	1	bleomycin
RA-021	Medulloblastoma Group 3	nil	nil	5	0		5	clofarabine, pentostatin, etoposide, teniposide, idarubicin	2	0		2	cladribine, decitabine
RA-034	Choroid plexus carcinoma	nil	High SRC	1	1	dasatinib**	0		0	0		0	
RA-055	DMG	PDGFRA, KIT, VEGFR2 amplification		0	0		0		1	1	nintedanib*	0	
RA-019	Ewing sarcoma	nil	High KIT	3	3	dinaciclib, panobinostat, cabozantinib**	0		2	1	alectinib	1	irinotecan
WE-012	Ewing sarcoma	STAG2 mutation<br>TP53 mutation (LOH)<br>EGFR copy gain	High EGFR	2	1	gefitinib †	1	irinotecan	0	0		0	
RA-054	Rhabdomyosarcoma	CDK4 amplification		2	2	buparlisib, voxtalisib	0		0	0		0	
RA-017	Osteosarcoma	nil	High CCNE1<br>High HDAC6<br>High CTNNB1	2	2	dinaciclib**<br>panobinostat**	0		2	2	crenolanib, PRI-724**	0	
WE-005	Osteosarcoma	no molecular data		2	2	temsirolimus, crizotinib	0		0	0		0	
WE-001	Undifferentiated sarcoma	nil	nil	1	0		1	pralatrexate	0	0		0	
RA-013	Neuroblastoma	nil	High BCL2<br>High ALK	4	2	venetoclax**, ceritinib**	2	melphalan, idarubicin	0	0		0	
RA-003	Neuroblastoma	nil	nil	0	0		0		0	0		0	
RA-027	Neuroblastoma	NF1 mutation (LOH)<br>CDKN2A/B biallelic loss	nil	0	0		0		0	0		0	
WE-006	Neuroblastoma	Nil	nil	0	0		0		0	0		0	
RA-048	DMG	PIK3CA mutation	nil	0	0		0		0	0		0	

* SNV correlation; † CNV correlation; ** RNA expression correlation. Abbreviation: DMG, diffuse midline glioma H3K27M mutant
Note: Leve 1: Z score AUC ≤ -2 and IC50 ≤ -2 and IC50 < Cmax or Css; Level 2: Z score AUC ≤ -2 and IC50 ≤ -2

Appendix Table S6. PDX drug treatment response in 19 models

ID	Diagnosis	Targetable aberrations	Chemotherapy	OR		Targeted agents	OR		Combination	OR	
WE-012	Ewing sarcoma	STAG2 mutation<br>TP53 mutation (LOH)	IRN + TMZ<br>Gemcitabine	PD2<br>PD2	0.0067<br>0.0067	Talazoparib*<br>Gefitinib †	PD2<br>PD1	0.051<br>0.13	IRN + TMZ + talazoparib*<br>Gemcitabine + gefitinib	CR<br>PD2	0.0067<br>0.0067
RA-001	Ewing sarcoma	STAG2 mutation<br>TP53 mutation (LOH)	IRN + TMZ	PD1	0.085	Talazoparib*	PD1	0.46	IRN + TMZ + talazoparib*	MCR	0.0067
RA-029	Alveolar<br>rhabdomyosarcoma	High FGFR4 expression	Nil			Ponatinib*<br>Palbociclib	PD1<br>PD1	0.11<br>0.68	Palbociclib + temsirolimus	PD1	0.063
RA-054	Alveolar<br>rhabdomyosarcoma	CDK4 amplification<br>high FGFR4 expression	Nil			Ponatinib*<br>Palbociclib*	PD1<br>PD1	0.0079<br>0.38	Palbociclib + temsirolimus*	PD1	0.0025
RA-017	Osteosarcoma	High CCNE1 expression	Cisplatin<br>IRN + TMZ	PD1<br>PD2	0.78<br>0.00067	Dinaciclib* †	PD1	0.78	Dinaciclib + cisplatin*	PD1	0.90
WE-005	Osteosarcoma	no molecular data	IRN + TMZ	PD1	0.085	Afursertib<br>Temsirolimus †	PD1<br>PD1	0.24<br>0.077	IRN + TMZ + temsirolimus	PD2	0.0067
WE-001	Undifferentiated<br>sarcoma	nil	Pralatrexate †<br>Carboplatin<br>Pralatrexate + carboplatin<br>IRN + TMZ	PD1<br>PD1<br>PD1<br>PD2	0.97<br>0.44<br>0.99<br>0.0067	Dinaciclib	PD1	0.24	nil		
RA-003	Neuroblastoma	nil	CYCLO + TOPO	MCR	0.0005	nil			nil		
RA-027	Neuroblastoma	NF1 mutation (LOH)<br>CDKN2A/B loss	IRN + TMZ<br>CYCLO + TOPO	PR<br>PR	0.0001<br>0.0001	Trametinib*<br>Isotretinoin	PD1<br>PD1	0.25<br>0.92	Trametinib + Isotretinoin*	PD1	0.0035
RA-013	Neuroblastoma	High BCL2 expression	Nil			Venetoclax* †	PD1	0.025	Nil		
RA-039	Neuroblastoma	ALK amplification	CYCLO + TOPO	SD	0.0006	Ceritinib*	CR	0.0015	CYCLO + TOPO + ceritinib*	MCR	0.0015
WE-006	Neuroblastoma	nil	IRN + TMZ	MCR	0.0011	Nil			IRN + TMZ + olaparib	MCR	0.0027
RA-049	Anaplastic large cell<br>lymphoma	ALK fusion	Nil			Ceritinib*<br>Alectinib*<br>Brentuximab	CR<br>MCR<br>PD1	0.0067<br>0.0067<br>0.085	Ceritinib + brentuximab*	MCR	0.018
RA-018	Pre-B ALL	CRLF2 fusion<br>JAK2 mutation<br>CDKN2A/B loss	VXL<br>Topotecan<br>Clofarabine	CR<br>CR<br>MCR	0.018<br>0.018<br>0.018	Bortezomib<br>Carfilzomib<br>Temsirolimus<br>Dasatinib	CR<br>PD1<br>PD1<br>PD1	0.018<br>0.018<br>0.018<br>0.18	Nil		
RA-004	Pre-B ALL	nil	VXL<br>Topotecan<br>Clofarabine	CR<br>PD2<br>CR	0.030<br>0.030<br>0.030	Temsirolimus<br>Venetoclax	PD2<br>PD2	0.009<br>0.030	Nil		
RA-045	T-ALL	NOTCH1 mutation<br>CDKN2A/B loss	VXL<br>Topotecan<br>Clofarabine<br>Nelarabine	MCR<br>MCR<br>CR<br>CR	0.38<br>0.38<br>0.63<br>0.65	Carfilzomib<br>Bortezomib<br>Temsirolimus<br>Venetoclax<br>Palbociclib*	SD<br>SD<br>SD<br>SD<br>SD	0.33<br>0.38<br>0.99<br>0.64<br>0.19	VXL + carfilzomib	MCR	0.38
				**T/C**	**P value**		**T/C**	**P value**		**T/C**	**P value**
RA-002	High grade glioma	TSC1 mutation (LOH)	TMZ	1.7	<0.0001	Temsirolimus* †<br>Ceritinib<br>BI236	3.9<br>2.6<br>4.1	<0.0001<br><0.0001<br><0.0001	Nil		
RA-028	High grade glioma	PDGFRA mutation<br>CDKN2A/B loss	Nil			Temsirolimus<br>Palbociclib*	0.8<br>0.9	0.20<br>0.81	Palbociclib + temsirolimus*	0.9	0.19
RA-021	Medulloblastoma<br>Group 3	nil	Vinorelbine<br>Gemcitabine	1.1<br>1.1	0.005<br>0.080	Nil			Nil		

Note: vehicle controls were included in all models; * agents targeting known molecular aberration; † HTS hit; OR objective response

Appendix Table S7. Dosing and schedule for PDX drug efficacy studies

Drugs	Schedule
RA-001	
Irinotecan	5 mg/kg, PO once daily x 5d, repeat on D21
Temozolomide	9.9 mg/kg, PO once daily x 5d, repeat on D21
Talazoparib	0.05 mg/kg, PO twice daily x 5d, repeat on D21
RA-002	
Temsirolimus	20mg/kg/day, IP 5d/week for 8 weeks
Ceritinib	25mg/kg/day, PO, 5d/week for 5 weeks
BI236	25mg/kg/day, IP 2d/week for 2 weeks
RA-003	
Cyclophosphamide	20 mg/kg, IP D1 to D5, repeat on D21
Topotecan	0.5 mg/kg, IP D1 to D5, repeat on D21
RA-004	
Vincristine	0.15 mg/kg, IP once a week for 4 weeks
Dexamethasone	5 mg/kg, IP 5d/week for 4 weeks
L-asparaginase	1000 IU/kg, IP 5d/week for 4 weeks
Topotecan	0.6mg/kg, IP 5d/week for 2 wks, repeat on D21
Clofarabine	30mg/kg, IP 5d/week for 4 weeks
Carfilzomib	1mg/kg, IV D1 and D2, repeat weekly for 4 wks
Temsirolimus	20mg/kg, IP 5d/week for 2 weeks
Vismodegib	100mg/kg PO 5d/week for 2 weeks
Venetoclax	100mg/kg PO once daily for 21 days
RA-013	
Venetoclax	100 mg/kg, oral gavage 21 days
RA-017	
Cisplatin	0.875 mg/kg, IP once every 21d for 6 weeks
Irinotecan	5 mg/kg, PO once daily x 5d, repeat on D21
Temozolomide	50 mg/kg, PO once daily x 5d, repeat on D21
Dinaciclib	5 mg/kg, IP twice/week for 2 wks, repeat on D21
RA-018	
Vincristine	0.15 mg/kg, IP once a week for 4 weeks
Dexamethasone	5 mg/kg, IP 5d/week for 4 weeks
L-asparaginase	1000 IU/kg, IP 5d/week for 4 weeks
Bortezomib	1mg/kg IP twice weekly x 6 weeks
Topotecan	0.6mg/kg, IP 5d/week for 2 wks, repeat on D21
Clofarabine	30mg/kg, IP 5d/week for 4 weeks
Carfilzomib	1mg/kg, IV D1 and D2, repeat weekly for 4 wks
Temsirolimus	20mg/kg, IP 5d/week for 2 weeks
Dasatinib	15mg/kg PO 5d/week for 4 weeks
RA-021	
Gemcitabine	60 mg/kg, IV once a week for 5 weeks
Vinorelbine	10 mg/kg, IP once a week for 3 weeks
RA-027	
Cyclophosphamide	10 mg/kg, IP D1 to D5
Topotecan	0.5 mg/kg, IP D1 to D5
Irinotecan	2 mg/kg, IP x 5d
Temozolomide	5 mg/kg, IP x 5d
Trametinib	2 mg/kg, oral gavage 5 d/week for 4 weeks
Isotretinoin	40 mg/kg, oral gavage 5 d/week for 4 weeks
RA-028	
Temsirolimus	20mg/kg/day, IP 5d/week for 6 weeks
Palbociclib	5mg/kg/day, PO 5d/week for 4 weeks
RA-029 and RA-054	
Ponatinib	30 mg/kg, PO once daily x 21d
Palbociclib	5.4 mg/kg, PO once daily x 21d
Temsirolimus	20 mg/kg, IP once daily 5d/week for 2 weeks
RA-039	
Cyclophosphamide	20 mg/kg, IP D1 to D5, repeat on D21
Topotecan	0.5 mg/kg, IP D1 to D5, repeat on D21
Ceritinib	50 mg/kg, oral gavage 28 days
RA-049	
Alectinib	60 mg/kg, PO once daily x 28d
Ceritinib	25 mg/kg, PO once daily x 28d
Brentuximab	1 mg/kg, IV twice a week for 6 weeks
RA-045	
Vincristine	0.15 mg/kg, IP once a week for 4 weeks
Dexamethasone	5 mg/kg, IP 5d/week for 4 weeks
L-asparaginase	1000 IU/kg, IP 5d/week for 4 weeks
Bortezomib	1mg/kg IP twice weekly x 6 weeks
Topotecan	0.6mg/kg, IP 5d/week for 2 wks, repeat on D21
Clofarabine	15 mg/kg, IP 5d/week for 4 weeks
Carfilzomib	1mg/kg, IV D1 and D2, repeat weekly for 4 wks
Temsirolimus	20mg/kg, IP 5d/week for 2 weeks
Nelarabine	150 mg/kg, IP x5 d
Venetoclax	100mg/kg PO once daily for 21 days
Palbociclib	5.4 mg/kg PO daily for 21 days
WE-001	
Carboplatin	30 mg/kg, IP on D1 and D4, repeat on D21
Irinotecan	5 mg/kg, PO once daily x 5d, repeat on D21
Temozolomide	50 mg/kg, PO once daily x 5d, repeat on D21
Pralatrexate	15 mg/kg, IP on D1 and D4, repeat on D21
Dinaciclib	5 mg/kg, IP twice/week for 2 wks, repeat on D21
WE-005	
Afursertib	75 mg/kg, PO once daily x 21d
Irinotecan	5 mg/kg, PO once daily x 5d, repeat on D21
Temozolomide	50 mg/kg, PO once daily x 5d, repeat on D21
Temsirolimus	20 mg/kg, IP once daily 5d/week for 2 weeks
WE-006	
Irinotecan	1.25 mg/kg, IP 5 d/week for 2 weeks
Temozolomide	8.25 mg/kg, oral gavage 5 days
Olaparib	50 mg/kg, oral gavage 5 d/week for 2 weeks
WE-012	
Irinotecan	5 mg/kg, PO once daily x 5d, repeat on D21
Temozolomide	9.9 mg/kg, PO once daily x 5d, repeat on D21
Gemcitabine	60 mg/kg, IV on D1 and D8, repeat on D21
Gefinitnib	150 mg/kg, PO once daily x 28d
Talazoparib	0.1 mg/kg, PO twice daily x 5d, repeat on D21

Appendix Table S8. Molecular aberrations which did not correlate with drug responses in HTS and PDX.

Patient ID	Diagnosis	Molecular target	Treatment without PDX responses	HTS hit
RA-001	EWS	TP53, STAG2 mut	Talazoparib	ND
RA-013	NBL	High BCL RNA	Venetoclax	ND
RA-017	OST	CCNE1 amplification	Dinaciclib<br>Dinaciclib + cisplatin	ND
RA-027	NBL	NF1 mutation with LOH	Trametinib<br>Trametinib + isotretinoin	No MEK inhibitors
		CDKN2A/B loss	ND	No CDK4/6 inhibitors
RA-028	HGG	CDKN2A/B loss	Palbociclib<br>Palbociclib + temsirolimus	No CDK4/6 inhibitors
RA-029	RMS	High FGFR4 RNA	Ponatinib	ND
RA-045	T-ALL	CDKN2A/B loss	Palbociclib	ND
RA-048	DMG	PIK3CA mutation	ND	No PI3K, AKT, mTOR inhibitors
RA-054	RMS	CDK4 amplification	Palbociclib<br>Palbociclib + temsirolimus	No CDK4/6 inhibitors
		High FGFR4 RNA	Ponatinib	ND
WE-012	EWS	TP53, STAG2 mut	Talazoparib	ND

Abbreviations: DMG, diffuse midline glioma; EWS, Ewings sarcoma; HGG, high grade glioma; LOH, loss of heterozygosity; ND, not done; NBL, neuroblastoma; OST, osteosarcoma; RMS, rhabdomyosarcoma; T-ALL, T-cell acute lymphoblastic leukemia.
