# Supplementary material for: In vitro and in vivo drug screens of tumor cells identify novel therapies for high‐risk child cancer
Source: EMBO Mol Med. 2021 Dec 20;14(4):e14608. doi: 10.15252/emmm.202114608 (PMC8988207; doi:10.15252/emmm.202114608)
Supplement: Supplementary file 2 — Expanded View Figures PDF [file EMMM-14-e14608-s007.pdf]

Expanded View Figures

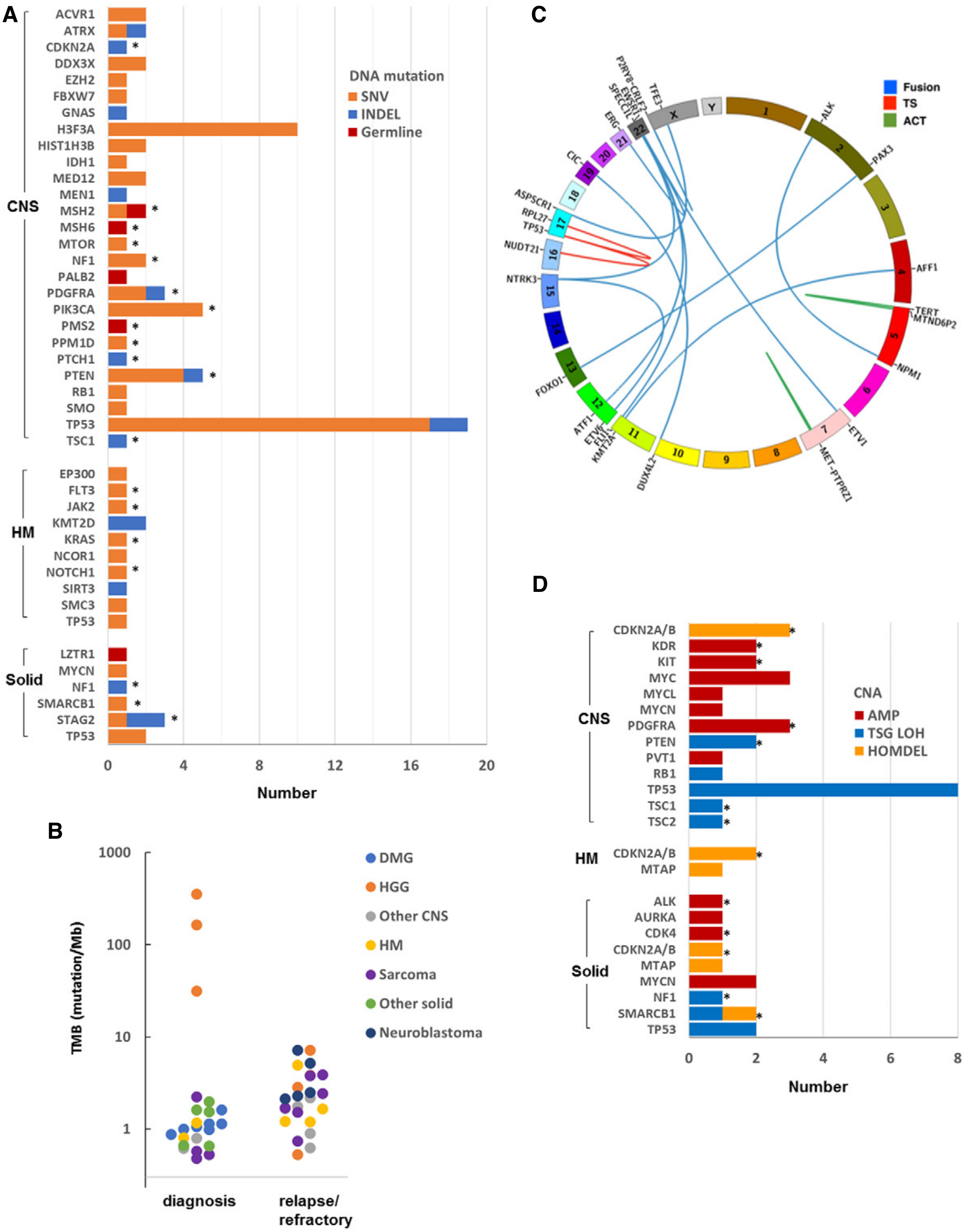

Figure EV1.

© 2021 The Authors

Figure EV1. Molecular aberrations in 55 pediatric high-risk cancers.

A Genes with somatic and germline DNA mutations (single-nucleotide variant (SNV) and indel) considered to be pathogenic or likely pathogenic by whole genome sequencing (WGS) and/or panel sequencing. Thirty of 55 samples were found to have 1 or more pathogenic or likely pathogenic mutations. The cohort consists of 27 central nervous system (CNS) tumors, 8 hematologic malignancies (HMs), and 20 non-CNS solid tumors. Targetable aberrations are indicated by asterisks.

B Tumor mutation burden (TMB) derived from WGS in 23 samples obtained at diagnosis and 24 samples at refractory/relapse.

C Structural variants (SVs) detected by WGS and/or RNA-seq in 55 samples. Seventeen reportable SVs included 13 fusions, 2 oncogenic activating (ACT) SVs, and 2 tumor suppressor (TS) loss-of-function SV.

D Reportable copy number variations (CNVs) included amplifications ($\geq$ 6 copies), loss of heterozygosity (LOH) associated with a loss-of-function mutation in a tumor suppressor gene (TSG LOH) and homozygous deletion (HOMDEL) of TSG. Twenty-four samples were found to have 1 or more reportable CNV. Targetable aberrations are indicated by asterisks.

© 2021 The Authors
