# Supplementary material for: In vitro and in vivo drug screens of tumor cells identify novel therapies for high‐risk child cancer
Source: EMBO Mol Med. 2021 Dec 20;14(4):e14608. doi: 10.15252/emmm.202114608 (PMC8988207; doi:10.15252/emmm.202114608)
Supplement: Supplementary file 3 — Table EV1 [file EMMM-14-e14608-s009.docx]

| **Table EV1. 111 compounds in HTS** | | | | | | | | | |
| --- | --- | --- | --- | --- | --- | --- | --- | --- | --- |
|  | **Cytotoxic** | **Target/mechanism** | **FDA** | **Pediatric dose** |  | **Targeted** | **Target/mechanism** | **FDA** | **Pediatric dose** |
| 1 | Azacytidine | anti-metabolite | y | y | 49 | Abiraterone | androgen | y | n |
| 2 | Bendamustine | alkylating mustard | y | y | 50 | Afuresertib | AKT | n | n |
| 3 | Bleomycin | DNA | y | y | 51 | Alectinib | ALK | y | n |
| 4 | Busulfan | alkylating | y | y | 52 | Alisertib | AURKA | n | y |
| 5 | Capecitabine | anti-metabolite | y | y | 53 | Alpelisib | PIK3CA | n | n |
| 6 | Carboplatin | alkylating | y | y | 54 | Axitinib | PDGFR, KIT, AURK, ABL, PLK4, CSF1R, FLT3 | y | y |
| 7 | Carmustine | alkylating mustard | y | y | 55 | Bortezomib | Proteosome | y | y |
| 8 | Chlorambucil | alkylating mustard | y | y | 56 | Buparlisib | PI3K-pan | n | n |
| 9 | Cisplatin | alkylating | y | y | 57 | Cabozantinib | MET, RET, VEGFR2 | y | y |
| 10 | Cladribine | anti-metabolite | y | y | 58 | Capmatinib | MET | n | n |
| 11 | Clofarabine | anti-metabolite | y | y | 59 | Carfilzomib | proteosome | y | n |
| 12 | Cytarabine | anti-metabolite | y | y | 60 | Ceritinib | ALK | y | y |
| 13 | Dacarbazine | alkylating | y | y | 61 | Cobimetinib | MEK | y | n |
| 14 | Dactinomycin | RNA | y | y | 62 | Crenolanib | FLT3, PDGFRA, PDGFRB | n | y |
| 15 | Daunorubicin | DNA anthracycline | y | y | 63 | Crizotinib | ALK, MET, ROS1 | y | y |
| 16 | Decitabine | anti-metabolite | y | y | 64 | Dabrafenib | BRAF | y | y |
| 17 | Docetaxel | microtubule | y | y | 65 | Dasatinib | ABL, PDGFR, SRC, EPHA, KIT, FYN, LCK, BLK, YES1, FRK | y | y |
| 18 | Doxorubicin | DNA anthracycline | y | y | 66 | Dinaciclib | CDK1/2/9 | n | n |
| 19 | Epirubicin | DNA anthracycline | y | y | 67 | Entrectinib | NTRK, ROS1, ALK | n | y |
| 20 | Etoposide | TOP2 | y | y | 68 | Erlotinib | EGFR | y | y |
| 21 | Fludarabine | anti-metabolite | y | y | 69 | Everolimus | mTOR | y | y |
| 22 | Fluorouracil | anti-metabolite | y | y | 70 | Fulvestrant | ESR1 | y | y |
| 23 | Gemcitabine | anti-metabolite | y | y | 71 | Gefitinib | EGFR | y | y |
| 24 | GENZ-644282 | TOP1 | n | n | 72 | Idasanutlin | MDM2 | n | n |
| 25 | Idarubicin | DNA anthracycline | y | y | 73 | Idelalisib | PIK3CD | y | n |
| 26 | Ifosfamide | alkylating | y | y | 74 | Imatinib | PDGFR, ABL, DDR1/2, KIT, CSF1R | y | y |
| 27 | Irinotecan | TOP1 | y | y | 75 | Ixazomib | Proteosome | y | n |
| 28 | Ixabepilone | microtubule | y | y | 76 | Lapatinib | ERBB2 | y | y |
| 29 | Lomustine | alkylating mustard | y | y | 77 | Letrozole | aromatase | y | y |
| 30 | Melphalan | alkylating | y | y | 78 | MK-2206 | AKT | n | y |
| 31 | Mercaptopurine | anti-metabolite | y | y | 79 | Nilotinib | ABL, DDR1/2, PDGFR, KIT | y | y |
| 32 | Methotrexate | anti-metabolite | y | y | 80 | Nintedanib | VEGFR, FGFR, PDGFR | y | n |
| 33 | Mitomycin | alkylating | y | y | 81 | Olaparib | PARP | y | n |
| 34 | Mitoxantrone | DNA | y | y | 82 | Omacetaxine | ribosome | y | y |
| 35 | Nelarabine | anti-metabolite | y | y | 83 | Palbociclib | CDK4/6 | Y | y |
| 36 | Oxaliplatin | alkylating | y | y | 84 | Panobinostat | HDAC | y | y |
| 37 | Paclitaxel | microtubule | y | y | 85 | Pazopanib | PDGFR, VEGFR1/2, KIT, CSF1R, DDR1/2 | y | y |
| 38 | Pemetrexed | anti-metabolite | y | y | 86 | Pentostatin | ADA | y | y |
| 39 | Pralatrexate | anti-metabolite | y | n | 87 | Pinometostat | DOT1L | n | y |
| 40 | Procarbazine | alkylating | y | y | 88 | Ponatinib | ABL, FLT3, RET, KIT, PDGFR, FGFR, VEGFR | y | n |
| 41 | Temozolomide | alkylating | y | y | 89 | PRI-724 | WNT | n | n |
| 42 | Teniposide | TOP2 | y | y | 90 | Quizartinib | FLT3 | n | y |
| 43 | Thioguanine | anti-metabolite | y | y | 91 | Ribociclib | CDK4/6 | Y | y |
| 44 | Thiotepa | alkylating | y | y | 92 | Rigosertib | PLK1 | n | n |
| 45 | Topotecan | TOP1 | y | y | 93 | Ruxolitinib | JAK1,2,3, TYK2 | y | y |
| 46 | Vinblastine | microtubule | y | y | 94 | Sirolimus | mTOR | y | y |
| 47 | Vincristine | microtubule | y | y | 95 | Sonidegib | SHH | y | y |
| 48 | Vinorelbine | microtubule | y | y | 96 | Sorafenib | VEGFR, PDGFR, Raf, KIT, RET, FGFR1, FLT3 | y | y |
|  |  |  |  |  | 97 | Sunitinib | PDGFR, KIT, FLT3, VEGFR, STK17A, CSF1R | y | y |
|  |  |  |  |  | 98 | Talazoparib | PARP | y | y |
|  |  |  |  |  | 99 | Tamoxifen | ESR1 | y | y |
|  |  |  |  |  | 100 | Tazemetostat | EZH2 | n | y |
|  |  |  |  |  | 101 | Temsirolimus | mTOR | y | y |
|  |  |  |  |  | 102 | Tofacitnib | JAK | y | y |
|  |  |  |  |  | 103 | Trametinib | MEK | y | y |
|  |  |  |  |  | 104 | Vandetanib | VEGFR, RET, EGFR | y | y |
|  |  |  |  |  | 105 | Veliparib | PARP | n | y |
|  |  |  |  |  | 106 | Vemurafenib | BRAF | y | y |
|  |  |  |  |  | 107 | Venetoclax | BCL2 | y | y |
|  |  |  |  |  | 108 | Vismodegib | SHH | y | y |
|  |  |  |  |  | 109 | Volasertib | PLK1 | n | n |
|  |  |  |  |  | 110 | Vorinostat | HDAC | y | y |
|  |  |  |  |  | 111 | Voxtalisib | PI3K/mTOR dual | n | n |
