# Supplementary material for: In vitro and in vivo drug screens of tumor cells identify novel therapies for high‐risk child cancer
Source: EMBO Mol Med. 2021 Dec 20;14(4):e14608. doi: 10.15252/emmm.202114608 (PMC8988207; doi:10.15252/emmm.202114608)
Supplement: Supplementary file 4 — Table EV2 [file EMMM-14-e14608-s005.docx]

| **Table EV2. Targetable aberrations, molecular therapeutic options, patient response and recommendations** | | | | | | | | | | | | | | |  | |
| --- | --- | --- | --- | --- | --- | --- | --- | --- | --- | --- | --- | --- | --- | --- | --- | --- |
| **Patient ID** | **Type** | **Diagnosis** | **Germ-line** | **Targetable SNV/indel** | **Targetable CNV** | **Targetable SV** | **Targeted agent/**  **inhibitor** | **Therapeutic options** | **Tier** | **Drug received** | **Response** | **No. of MOL. guided REC** | **No. of non-molecular REC guided by HTS/PDX** | **Total no. of REC** | |  |
| RA-049 | HM | ALCL | 0 | 0 | 0 | NPM1-ALK | ALK | ALKi | 1 | ceritinib | CR | 1 | 0 | 1 | |  |
| RA-031 | CNS | HGG | 0 | 0 | CNKN2A/B bi-loss | ETV6-NTRK3 | NTRK | NTRKi | 2 | larotrectinib | PR | 1 | Not done | 1 | |  |
| RA-057 | Solid | IFS | 0 | 0 | 0 | SPECC1L-NTRK3 | NTRK | NTRKi | 1 | larotrectinib | PR | 1 | Not done | 1 | |  |
| RA-024 | CNS | HGG | MSH6 | High TMB | 0 | 0 | ICI | ICI | 1 | nivolumab | SD | 1 | Not done | 1 | |  |
| RA-048 | CNS | DMG | 0 | PIK3CA | 0 | 0 | PI3K or mTOR | mTORi | 4 | sirolimus | PD | 1 | 0 | 1 | |  |
| RA-039 | Solid | NB | 0 | 0 | ALK 51 copies | 0 | ALK | ALKi | 3 | ceritinib | PD | 1 | 0 | 1 | |  |
| RA-002 | CNS | HGG | 0 | TSC1 | TSC1 LOH | 0 | mTOR | mTORi | 2 | sirolimus | PD | 1 | 1 | 2 | |  |
| WE-012 | Solid | EWS | 0 | STAG2, TP53 | 0 | 0 | PARP | PARPi+ TMZ or IRN | 3 | olaparib+IRN | PD | 1 | 1 | 2 | |  |
| RA-030 | CNS | HGG | MSH2 | High TMB | 0 | 0 | ICI | ICI | 1 | nivolumab | PD | 1 | Not done | 1 | |  |
| RA-007 | CNS | DMG | 0 | mTOR PPM1D | 0 | 0 | mTOR PARP | mTORi PARPi+TMZ | 4 4 | sirolimus | PD | 2 | Not done | 2 | |  |
| RA-033 | CNS | DMG | 0 | 0 | PDGFRA 28 copies | 0 | PDGFR | PDGFRi | 4 | dasatinib | PD | 1 | Not done | 1 | |  |
| RA-037 | CNS | DMG | 0 | PIK3CA | 0 | 0 | PI3K or mTOR | mTORi | 4 | sirolimus | PD | 1 | Not done | 1 | |  |
| RA-044 | CNS | DMG | 0 | PTEN | PTEN LOH PDGFRA 62 copies KIT 46 copies KDR 35 copies | 0 | mTOR PDGFR KIT VEGFR | mTORi+PDGFR/KITi | 4 4 | sirolimus+ dasatinib | PD | 2 | Not done | 2 | |  |
| WE-011 | CNS | DMG | 0 | PIK3CA | 0 | 0 | PI3K or mTOR | mTORi | 4 | sirolimus | PD | 1 | Not done | 1 | |  |
| RA-018 | HM | Pre-B ALL | 0 | JAK2 | CNKN2A/B bi-loss | P2RY8-CRLF2 | JAK2 CDK4/6 | JAK2i CDK4/6i | 3 4 | 0 | 0 | 2 | 1 | 3 | |  |
| RA-027 | Solid | NB | 0 | NF1 | NF1 LOH CNKN2A/B bi-loss | 0 | MEK CDK4/6 | MEKi CDK4/6i | 3 4 | 0 | 0 | 2 | 1 | 3 | |  |
| RA-028 | CNS | HGG | 0 | PDGFRA | CNKN2A/B bi-loss | 0 | PDGFR CDK4/6 | PDGFRi CDK4/6i | 4 4 | 0 | 0 | 2 | 1 | 3 | |  |
| RA-045 | HM | T-ALL | 0 | NOTCH1 | CNKN2A/B bi-loss | 0 | gamma secretase  CDK4/6 | 0 CDK4/6i | 3 | 0 | 0 | 1 | 1 | 2 | |  |
| RA-054 | Solid | RMS | 0 | 0 | CDK4 38 copies | 0 | CDK4/6 | CDK4/6i+mTORi | 2 | 0 | 0 | 1 | 1 | 2 | |  |
| RA-056 | CNS | HGG | PMS2 | hypermutated | 0 | 0 | ICI | ICI | 1 | 0 | 0 | 1 | 1 | 2 | |  |
| RA-001 | Solid | EWS | 0 | STAG2, TP53 | 0 | 0 | PARP | PARPi+ TMZ or IRN | 3 | 0 | 0 | 1 | 0 | 1 | |  |
| RA-055 | CNS | DMG | 0 | 0 | PDGFRA 55 copies KIT 40 copies KDR 27 copies | 0 | PDGFR KIT VEGFR | PDGFR/KITi | 4 | 0 | 0 | 1 | 0 | 1 | |  |
| RA-023 | HM | AUL | 0 | FLT3 | 0 | 0 | FLT3 | FLT3i | 2 | 0 | 0 | 1 | Not done | 1 | |  |
| RA-050 | HM | B-ALL | 0 | KRAS | 0 | 0 | MEK | MEKi | 3 | 0 | 0 | 1 | Not done | 1 | |  |
| RA-032 | CNS | DMG | 0 | PDGFRA | 0 | 0 | PDGFR | PDGFRi | 5 | 0 | 0 | 1 | Not done | 1 | |  |
| WE-010 | Solid | MRT | 0 | SMARCB1 | SMARCB1 CN-LOH | 0 | EZH2 | EZH2i | 3 | 0 | 0 | 1 | Not done | 1 | |  |
| RA-010 | CNS | DMG | 0 | PTEN | PTEN LOH | 0 | mTOR | mTORi | 4 | 0 | 0 | 1 | Not done | 1 | |  |
| RA-047 | Solid | MyoE | 0 | 0 | SMARCB1 bi-loss | 0 | EZH2 | EZH2i | 2 | 0 | 0 | 1 | Not done | 1 | |  |
| WE-008 | CNS | HGG | 0 | 0 | CNKN2A/B bi-loss | PTPRZ1-MET | MET | METi | 2 | 0 | 0 | 2 | Not done | 2 | |  |
| RA-025 | CNS | MB | 0 | PTCH1 | 0 | 0 | SMO | 0 | 0 | 0 | 0 | 0 | Not done | 0 | |  |
| RA-017 | Solid | OST | 0 | 0 | 0 | 0 | 0 | 0 | 0 | 0 | 0 | 0 | 2 | 2 | |  |
| WE-005 | Solid | OST | na | na | na | na | na | na | na | na | na | 0 | 2 | 2 | |  |
| RA-003 | Solid | NB | 0 | 0 | 0 | 0 | 0 | 0 | 0 | 0 | 0 | 0 | 1 | 1 | |  |
| RA-004 | HM | B-ALL | 0 | 0 | 0 | 0 | 0 | 0 | 0 | 0 | 0 | 0 | 1 | 1 | |  |
| RA-013 | Solid | NB | 0 | 0 | 0 | 0 | 0 | 0 | 0 | 0 | 0 | 0 | 1 | 1 | |  |
| RA-019 | Solid | EWS | 0 | 0 | 0 | 0 | 0 | 0 | 0 | 0 | 0 | 0 | 1 | 1 | |  |
| RA-021 | CNS | MB | 0 | 0 | 0 | 0 | 0 | 0 | 0 | 0 | 0 | 0 | 1 | 1 | |  |
| RA-034 | CNS | CPC | 0 | 0 | 0 | 0 | 0 | 0 | 0 | 0 | 0 | 0 | 1 | 1 | |  |
| WE-001 | Solid | Unsarc | 0 | 0 | 0 | 0 | 0 | 0 | 0 | 0 | 0 | 0 | 1 | 1 | |  |
| WE-006 | Solid | NB | 0 | 0 | 0 | 0 | 0 | 0 | 0 | 0 | 0 | 0 | 1 | 1 | |  |
| RA-029 | Solid | RMS | 0 | 0 | 0 | 0 | 0 | 0 | 0 | 0 | 0 | 0 | 0 | 0 | |  |
| RA-005 | HM | AML | 0 | 0 | 0 | 0 | 0 | 0 | 0 | 0 | 0 | 0 | Not done | 0 | |  |
| RA-006 | CNS | EPN | 0 | 0 | 0 | 0 | 0 | 0 | 0 | 0 | 0 | 0 | Not done | 0 | |  |
| RA-008 | CNS | MB | 0 | 0 | 0 | 0 | 0 | 0 | 0 | 0 | 0 | 0 | Not done | 0 | |  |
| RA-009 | Solid | HCC | 0 | 0 | 0 | 0 | 0 | 0 | 0 | 0 | 0 | 0 | Not done | 0 | |  |
| RA-011 | Solid | MNTI | 0 | 0 | 0 | 0 | 0 | 0 | 0 | 0 | 0 | 0 | Not done | 0 | |  |
| RA-016 | HM | PTCL | 0 | 0 | 0 | 0 | 0 | 0 | 0 | 0 | 0 | 0 | Not done | 0 | |  |
| RA-022 | CNS | MB | 0 | 0 | 0 | 0 | 0 | 0 | 0 | 0 | 0 | 0 | Not done | 0 | |  |
| RA-038 | CNS | DMG | PALB2 | 0 | 0 | 0 | 0 | 0 | 0 | 0 | 0 | 0 | Not done | 0 | |  |
| RA-040 | Solid | GNET | LZTR1 | 0 | 0 | 0 | 0 | 0 | 0 | 0 | 0 | 0 | Not done | 0 | |  |
| RA-042 | CNS | DMG | 0 | 0 | 0 | 0 | 0 | 0 | 0 | 0 | 0 | 0 | Not done | 0 | |  |
| RA-043 | CNS | MB | 0 | 0 | 0 | 0 | 0 | 0 | 0 | 0 | 0 | 0 | Not done | 0 | |  |
| RA-046 | Solid | ASPS | 0 | 0 | 0 | 0 | 0 | 0 | 0 | 0 | 0 | 0 | Not done | 0 | |  |
| RA-052 | CNS | DMG | 0 | 0 | 0 | 0 | 0 | 0 | 0 | 0 | 0 | 0 | Not done | 0 | |  |
| WE-002 | CNS | DMG | 0 | 0 | 0 | 0 | 0 | 0 | 0 | 0 | 0 | 0 | Not done | 0 | |  |
| WE-009 | Solid | MNTI | 0 | 0 | 0 | 0 | 0 | 0 | 0 | 0 | 0 | 0 | Not done | 0 | |  |

AML, Acute myeloid leukemia; AUL, Acute undifferentiated leukemia; ASPS, Alveolar soft part sarcoma; ALCL, Anaplastic large cell lymphoma; CPC, Choroid plexus carcinoma; DMG, Diffuse midline glioma; EPN, Ependymoma; EWS, Ewing's sarcoma; Gastrointestinal neuroectodermal tumor, GNET; HCC, Hepatocellular carcinoma; HGG, High grade glioma; IFS, Infantile fibrosarcoma; MB, Medulloblastoma; MNTI, Melanotic neuroectodermal tumour of infancy; MRT, Malignant rhabdoid tumour; MyoE, Myoepithelial tumour; NB, Neuroblastoma; OST, Osteosarcoma; PTCL, Peripheral T-cell lymphoma; REC, recommendation; RMS, Rhabdomyosarcoma; Unsarc, Undifferentiated sarcoma
