# Supplementary material for: In vitro and in vivo drug screens of tumor cells identify novel therapies for high‐risk child cancer
Source: EMBO Mol Med. 2021 Dec 20;14(4):e14608. doi: 10.15252/emmm.202114608 (PMC8988207; doi:10.15252/emmm.202114608)
Supplement: Supplementary file 5 — Table EV3 [file EMMM-14-e14608-s002.docx]

| **Table EV3. Reportable C4/C5 pathogenic variants** | | | | | |
| --- | --- | --- | --- | --- | --- |
| **Patient ID** | **Sample ID** | **Chr** | **Position** | **Genomic_Change** | **Consequence** |
| RA-001 | zcc210 | X | 123200076 | NM_001042749(STAG2):c.2152dupA (p.Thr718AsnfsTer10) | frameshift_variant |
| RA-001 | zcc210 | 17 | 7578479 | NM_000546(TP53):c.451C>A (p.Pro151Thr) | missense_variant |
| RA-001 | zcc210 | X | 123200080 | NM_006603(STAG2):c.2152dup (p.Thr718Asnfs*10) | frameshift_variant |
| RA-002 | zcc212 | 17 | 7577057 | NM_000546(TP53):c.880del (p.Glu294Serfs*51) | frameshift_variant |
| RA-002 | zcc212 | 9 | 135778078 | NM_001162426(TSC1):c.2304dupT (p.Asp769Ter) | frameshift_variant |
| RA-004 | zcc214 | 11 | 233377 | NM_012239(SIRT3):c.438delC (p.Gly147AlafsTer126) | frameshift_variant |
| RA-007 | zcc471 | 1 | 226252135 | ENST00000366813(H3F3A):c.83A>T (p.Lys28Met) | missense_variant |
| RA-007 | zcc471 | 1 | 11174395 | NM_004958(MTOR):c.7280T>G (p.Leu2427Arg) | missense_variant |
| RA-007 | zcc471 | 17 | 58740362 | NM_003620(PPM1D):c.1267G>T (p.Glu423Ter) | stop_gained |
| RA-010 | zcc472 | 17 | 7578406 | NM_001126112(TP53):c.524G>A (p.Arg175His) | missense_variant |
| RA-010 | zcc472 | 1 | 226252135 | ENST00000366813(H3F3A):c.83A>T (p.Lys28Met) | missense_variant |
| RA-010 | zcc472 | 10 | 89692847 | NM_000314(PTEN):c.331T>C (p.Trp111Arg) | missense_variant |
| RA-010 | zcc472 | 17 | 7576927 | NM_001126112(TP53):c.920-1G>A | splice_acceptor_variant |
| RA-010 | zcc472 | 9 | 21971125 | NM_000077(CDKN2A):c.232delC (p.Leu78SerfsTer68) | frameshift_variant |
| RA-010 | zcc472 | X | 76829806 | NM_000489(ATRX):c.6235C>T (p.Arg2079Ter) | stop_gained |
| RA-018 | zcc226 | 12 | 49415868 | NM_003482(KMT2D):c.16478_16479insCCTC (p.Lys5493Asnfs*20) | frameshift_variant |
| RA-018 | zcc226 | 12 | 49447000 | NM_003482(KMT2D):c.943dupT (p.Trp315LeufsTer27) | frameshift_variant |
| RA-018 | zcc226 | 9 | 5090497 | NM_004972(JAK2):c.2813G>A (p.Arg938Gln) | missense_variant |
| RA-022 | zcc229 | 20 | 57484253 | NM_001077489(GNAS):c.524_525delAT (p.Tyr175CysfsTer19) | frameshift_variant |
| RA-022 | zcc229 | 4 | 153247366 | NM_033632(FBXW7):c.1436G>A (p.Arg479Gln) | missense_variant |
| RA-022 | zcc229 | 7 | 148511172 | NM_004456(EZH2):c.1730C>T (p.Pro577Leu) | missense_variant |
| RA-022 | zcc229 | X | 41203603 | NM_001356(DDX3X):c.976C>T (p.Arg326Cys) | missense_variant |
| RA-023 | zcc230 | 17 | 16075230 | NM_001190440(NCOR1):c.322C>T (p.Arg108*) | stop_gained |
| RA-023 | zcc230 | 13 | 28592641 | NM_004119(FLT3):c.2504A>T (p.Asp835Val) | missense_variant |
| RA-023 | zcc230 | 22 | 41566521 | NM_001429(EP300):c.4398G>C (p.Trp1466Cys) | missense_variant |
| RA-023 | zcc230 | 10 | 112359416 | NM_005445(SMC3):c.2273G>A (p.Arg758His) | missense_variant |
| RA-024 | zcc231 | 13 | 48955538 | NM_000321(RB1):c.1654C>T (p.Arg552*) | stop_gained |
| RA-024 | zcc231 | 17 | 7578212 | NM_000546(TP53):c.637C>T (p.Arg213*) | stop_gained |
| RA-024 | zcc231 | 10 | 89685313 | NM_000314(PTEN):c.208C>T (p.Leu70Phe) | missense_variant |
| RA-024 | zcc231 | 10 | 89717727 | NM_000314(PTEN):c.752G>A (p.Gly251Asp) | missense_variant |
| RA-024 | zcc231 | 17 | 7577106 | NM_000546(TP53):c.832C>T (p.Pro278Ser) | missense_variant |
| RA-025 | zcc232 | 7 | 128846031 | NM_005631(SMO):c.961G>A (p.Val321Met) | missense_variant |
| RA-025 | zcc232 | 9 | 98218574 | NM_001083602(PTCH1):c.3086_3091dup (p.Phe1030_Thr1031insLysPhe) | inframe_insertion |
| RA-025 | zcc232 | X | 41205855 | NM_001356(DDX3X):c.1595C>T (p.Thr532Met) | missense_variant |
| RA-027 | zcc233 | 17 | 29662048 | NM_000267(NF1):c.5943del (p.Ile1982Leufs*9) | frameshift_variant |
| RA-027 | zcc233 | 2 | 16082317 | NM_005378(MYCN):c.131C>T (p.Pro44Leu) | missense_variant |
| RA-028 | zcc234 | 17 | 7578518 | NM_000546(TP53):c.412G>C (p.Ala138Pro) | missense_variant |
| RA-028 | zcc234 | 4 | 55140746 | NM_006206(PDGFRA):c.1607T>A (p.Val536Glu) | missense_variant |
| RA-028 | zcc234 | X | 70339230 | NM_005120(MED12):c.107T>G (p.Leu36Arg) | missense_variant |
| RA-030 | zcc236 | 11 | 64572092 | NM_130799(MEN1):c.1546delC (p.Arg516GlyfsTer43) | frameshift_variant |
| RA-030 | zcc236 | 17 | 7574003 | NM_000546(TP53):c.1024C>T (p.Arg342*) | stop_gained |
| RA-030 | zcc236 | 17 | 29679366 | NM_000267(NF1):c.7486C>T (p.Arg2496*) | stop_gained |
| RA-030 | zcc236 | 2 | 47656951 | NM_000251(MSH2):c.1147C>T (p.Arg383*) | stop_gained |
| RA-030 | zcc236 | 17 | 7577121 | NM_000546(TP53):c.817C>T (p.Arg273Cys) | missense_variant |
| RA-030 | zcc236 | 3 | 178916891 | NM_006218(PIK3CA):c.278G>A (p.Arg93Gln) | missense_variant |
| RA-030 | zcc236 | 3 | 178917478 | NM_006218(PIK3CA):c.353G>A (p.Gly118Asp) | missense_variant |
| RA-031 | zcc237 | 17 | 7578508 | NM_000546(TP53):c.422G>A (p.Cys141Tyr) | missense_variant |
| RA-032 | zcc238 | 1 | 226252135 | ENST00000366813(H3F3A):c.83A>T (p.Lys28Met) | missense_variant |
| RA-032 | zcc238 | 4 | 55140732 | NM_006206(PDGFRA):c.1594_1614dupCTGGTGCTGTTGGTGATTGTG (p.Leu532_Val538dup) | inframe_insertion |
| RA-033 | zcc239 | 1 | 226252135 | ENST00000366813(H3F3A):c.83A>T (p.Lys28Met) | missense_variant |
| RA-033 | zcc239 | 17 | 7578454 | NM_000546(TP53):c.470_475del (p.Val157_Arg158del) | inframe_deletion |
| RA-037 | zcc474 | 17 | 7578406 | NM_001126112(TP53):c.524G>A (p.Arg175His) | missense_variant |
| RA-037 | zcc474 | 1 | 226252135 | ENST00000366813(H3F3A):c.83A>T (p.Lys28Met) | missense_variant |
| RA-037 | zcc474 | 3 | 178936091 | NM_006218(PIK3CA):c.1633G>A (p.Glu545Lys) | missense_variant |
| RA-037 | zcc474 | 17 | 7577548 | NM_001126112(TP53):c.733G>A (p.Gly245Ser) | missense_variant |
| RA-037 | zcc474 | 17 | 29592291 | NM_000267(NF1):c.4706T>A (p.Leu1569Ter) | stop_gained |
| RA-038 | zcc241 | 2 | 158622516 | NM_001105(ACVR1):c.983G>T (p.Gly328Val) | missense_variant |
| RA-038 | zcc241 | 6 | 26032206 | NM_003537(HIST1H3B):c.83A>T (p.Lys28Met) | missense_variant |
| RA-044 | zcc245 | 1 | 226252135 | ENST00000366813(H3F3A):c.83A>T (p.Lys28Met) | missense_variant |
| RA-044 | zcc245 | 10 | 89717715 | NM_000314(PTEN):c.741dupA (p.Pro248ThrfsTer5) | frameshift_variant |
| RA-044 | zcc245 | 17 | 7577111 | NM_000546(TP53):c.827C>G (p.Ala276Gly) | missense_variant |
| RA-045 | zcc246 | 9 | 139399344 | NM_017617(NOTCH1):c.4799T>C (p.Leu1600Pro) | missense_variant |
| RA-048 | zcc249 | 1 | 226252135 | ENST00000366813(H3F3A):c.83A>T (p.Lys28Met) | missense_variant |
| RA-048 | zcc249 | 3 | 178936091 | NM_006218(PIK3CA):c.1633G>A (p.Glu545Lys) | missense_variant |
| RA-050 | zcc476 | 12 | 25398284 | NM_033360(KRAS):c.35G>A (p.Gly12Asp) | missense_variant |
| RA-050 | zcc476 | 17 | 7577538 | NM_001126112(TP53):c.743G>A (p.Arg248Gln) | missense_variant |
| RA-052 | zcc251 | 1 | 226252135 | ENST00000366813(H3F3A):c.83A>T (p.Lys28Met) | missense_variant |
| RA-052 | zcc251 | 17 | 7577548 | NM_001126112(TP53):c.733G>A (p.Gly245Ser) | missense_variant |
| RA-055 | zcc477 | 17 | 7578406 | NM_001126112(TP53):c.524G>A (p.Arg175His) | missense_variant |
| RA-055 | zcc477 | 1 | 226252135 | ENST00000366813(H3F3A):c.83A>T (p.Lys28Met) | missense_variant |
| RA-056 | zcc208 | 17 | 7577022 | NM_001126112(TP53):c.916C>T (p.Arg306Ter) | stop_gained |
| RA-056 | zcc208 | 17 | 7577539 | NM_001126112(TP53):c.742C>T (p.Arg248Trp) | missense_variant |
| RA-056 | zcc208 | 4 | 55144143 | NM_006206(PDGFRA):c.1972G>A (p.Val658Ile) | missense_variant |
| WE-002 | zcc468 | 1 | 226252135 | ENST00000366813(H3F3A):c.83A>T (p.Lys28Met) | missense_variant |
| WE-002 | zcc468 | 17 | 7574018 | NM_001126112(TP53):c.1009C>T (p.Arg337Cys) | missense_variant |
| WE-008 | zcc218 | 10 | 89717629 | NM_000314(PTEN):c.654C>A (p.Cys218*) | stop_gained |
| WE-008 | zcc218 | X | 76938079 | NM_000489(ATRX):c.2668delT (p.Ser890GlnfsTer15) | frameshift_variant |
| WE-008 | zcc218 | 17 | 7577141 | NM_000546(TP53):c.797G>A (p.Gly266Glu) | missense_variant |
| WE-008 | zcc218 | 2 | 209113113 | NM_005896(IDH1):c.394C>G (p.Arg132Gly) | missense_variant |
| WE-010 | zcc221 | 22 | 24135834 | NM_003073(SMARCB1):c.321C>A (p.Tyr107Ter) | stop_gained |
| WE-011 | zcc206 | 2 | 158622516 | NM_001105(ACVR1):c.983G>A (p.Gly328Glu) | missense_variant |
| WE-011 | zcc206 | 6 | 26032206 | NM_003537(HIST1H3B):c.83A>T (p.Lys28Met) | missense_variant |
| WE-011 | zcc206 | 3 | 178927980 | NM_006218(PIK3CA):c.1258T>C (p.Cys420Arg) | missense_variant |
| WE-011 | zcc206 | X | 70339253 | NM_005120(MED12):c.130G>A (p.Gly44Ser) | missense_variant |
| WE-012 | zcc207 | 17 | 7578272 | NM_000546(TP53):c.577C>T (p.His193Tyr) | missense_variant |
| WE-012 | zcc207 | X | 123224542 | NM_001042749(STAG2):c.3395T>G (p.Leu1132Ter) | stop_gained |
