# Supplementary material for: In vitro and in vivo drug screens of tumor cells identify novel therapies for high‐risk child cancer
Source: EMBO Mol Med. 2021 Dec 20;14(4):e14608. doi: 10.15252/emmm.202114608 (PMC8988207; doi:10.15252/emmm.202114608)
Supplement: Supplementary file 6 — Table EV4 [file EMMM-14-e14608-s001.docx]

| **Table EV4. Comprehensive cancer panel gene list** | | | | | | | | | | | | | | | | |  |
| --- | --- | --- | --- | --- | --- | --- | --- | --- | --- | --- | --- | --- | --- | --- | --- | --- | --- |
| **CCPv1** |  | |  | |  | |  | |  | |  | |  | |  | |  |
| ABL1 | CARD11 | | CYP3A5 | | EXT2 | | GSTM1 | | KRAS | | MLL2 | | PARK2 | | RAD21 | |  |
| ABL2 | CASP8 | | D10S170 | | EYS | | GSTP1 | | KTN1 | | MLL3 | | PARP10 | | RAD50 | |  |
| ACVR1B | CBFB | | DAXX | | EZH2 | | GTF3C4 | | LETM1 | | MLL4 | | PAX5 | | RAD51 | |  |
| ACVR2A | CBL | | DCUN1D1 | | FADD | | GUCY1A2 | | LETM2 | | MPHOSPH8 | | PAX8 | | RAD51C | |  |
| AGBL4 | CBLB | | DDB2 | | FAM123B | | H3F3A | | LGALS7 | | MPL | | PAX9 | | RAD51D | |  |
| AIP | CBLC | | DDR2 | | FAM190A | | H3F3C | | LIFR | | MRAS | | PBRM1 | | RAF1 | |  |
| AJUBA | CBX8 | | DDX11 | | FAM46C | | HDAC10 | | LINC00290 | | MRE11A | | PBX1 | | RARA | |  |
| AKT1 | CCDC6 | | DDX3X | | FANCA | | HDAC2 | | LINC00356 | | MRPL4 | | PCBP1 | | RASSF1 | |  |
| AKT2 | CCND1 | | DICER1 | | FANCB | | HGF | | LMO1 | | MRPS28 | | PCM1 | | RB1 | |  |
| AKT3 | CCND2 | | DIS3L2 | | FANCC | | HIF1A | | LRP1B | | MSH2 | | PDE4D | | RBFOX1 | |  |
| ALK | CCND3 | | DKK1 | | FANCD2 | | HIF2A | | LRRK2 | | MSH6 | | PDGFRA | | RECQL4 | |  |
| ANKS1B | CCNE1 | | DMD | | FANCE | | HIST1H1C | | LTK | | MST1 | | PDGFRB | | REG4 | |  |
| APC | CD79A | | DMP1 | | FANCF | | HIST1H2BD | | MACROD2 | | MTDH | | PHF12 | | REL | |  |
| AR | CD79B | | DNM2 | | FANCG | | HIST1H3B | | MADH4 | | MTOR | | PHF3 | | RET | |  |
| ARAF | CDC6 | | DNMT1 | | FANCI | | HMGA2 | | MAFA | | MUTYH | | PHF6 | | RHBDF2 | |  |
| ARFRP1 | CDC73 | | DNMT3A | | FANCL | | HNF1A | | MALAT1 | | MYB | | PHGDH | | RICTOR | |  |
| ARHGAP35 | CDH1 | | DPP6 | | FANCM | | HOOK3 | | MAP2K1 | | MYC | | PHLPP2 | | RNF43 | |  |
| ARID1A | CDH2 | | DYPD | | FAS | | HOXA3 | | MAP2K2 | | MYCL1 | | PHOX2B | | ROBO1 | |  |
| ARID1B | CDH20 | | DYRK1B | | FAT1 | | HPSE2 | | MAP2K4 | | MYCN | | PHRF1 | | ROBO2 | |  |
| ARID2 | CDH5 | | E2F3 | | FBXO11 | | HRAS | | MAP2K5 | | MYD88 | | PIK3C3 | | ROS1 | |  |
| ARID5B | CDH6 | | ECT2L | | FBXW7 | | HRPT2 | | MAP2K6 | | MYO3A | | PIK3CA | | RPL22 | |  |
| ASXL1 | CDK12 | | EEF1A2 | | FGF23 | | HSP90AA1 | | MAP2K7 | | MYO5B | | PIK3CG | | RPL5 | |  |
| ATM | CDK4 | | EGFR | | FGFR1 | | HSP90AB1 | | MAP3K1 | | MYOC | | PIK3R1 | | RPS14 | |  |
| ATR | CDK6 | | EGR3 | | FGFR2 | | ID1 | | MAP3K11 | | NAV3 | | PIK3R2 | | RPS6KB1 | |  |
| ATRX | CDK8 | | EHF | | FGFR3 | | IDH1 | | MAP3K12 | | NBN | | PIK3R3 | | RPTOR | |  |
| AURKA | CDKN1A | | EIF4A2 | | FGFR4 | | IDH2 | | MAP3K13 | | NBS1 | | PLCG1 | | RQCD1 | |  |
| AURKB | CDKN1B | | EIF5A2 | | FH | | IGF1R | | MAP3K14 | | NCOA2 | | PML | | RRM2B | |  |
| AXIN1 | CDKN1C | | ELF3 | | FHIT | | IGF2R | | MAP3K2 | | NCOA3 | | PMS1 | | RSPO2 | |  |
| AXIN2 | CDKN2A | | ELK3 | | FLCN | | IKBKB | | MAP3K3 | | NCOA4 | | PMS2 | | RSPO3 | |  |
| B2M | CDKN2B | | ELKS | | FLT1 | | IKBKE | | MAP3K4 | | NCOR1 | | POLQ | | RUNX1 | |  |
| B4GALT3 | CDKN2C | | EML4 | | FLT3 | | IKZF1 | | MAP3K5 | | NEDD9 | | POU1F1 | | SBDS | |  |
| BAG4 | CEBPA | | EP300 | | FLT4 | | IKZF2 | | MAP3K5 | | NEGR1 | | POU5F1 | | SDH5 | |  |
| BAP1 | CEP57 | | EP400 | | FOXA1 | | IL6ST | | MAP3K6 | | NF1 | | PPM1D | | SDHA | |  |
| BARD1 | CHD1 | | EPCAM | | FOXA2 | | IL7R | | MAP3K7 | | NF2 | | PPP2R1A | | SDHAF2 | |  |
| BCL11A | CHD1L | | EPHA3 | | FOXK2 | | IMMP2L | | MAP3K8 | | NFE2L2 | | PPP2R2A | | SDHB | |  |
| BCL2 | CHD8 | | EPHA5 | | FOXL2 | | ING1 | | MAP3K9 | | NFE2L3 | | PRDM1 | | SDHC | |  |
| BCL2A1 | CHEK1 | | EPHA6 | | FOXO1 | | ING5 | | MAP4K3 | | NGFR | | PRDM9 | | SDHD | |  |
| BCL2L1 | CHEK2 | | EPHA7 | | FOXP4 | | INHBA | | MAP4K4 | | NKX2-1 | | PRELID1 | | SETBP1 | |  |
| BCL2L2 | CIC | | EPHB1 | | FUBP1 | | INPP4B | | MAPK1 | | NOTCH1 | | PREX2 | | SETD2 | |  |
| BCL6 | CKS1B | | EPHB4 | | G6PD | | INSR | | MAPK10 | | NOTCH2 | | PRF1 | | SETD3 | |  |
| BCOR | CNTN4 | | EPHB6 | | GAB2 | | INTS4 | | MAPK7 | | NOTCH3 | | PRG4 | | SF3B1 | |  |
| BCORL1 | COL22A1 | | EPPK1 | | GABRG1 | | IRS2 | | MAPK8 | | NOTCH4 | | PRKAR1A | | SFTPA1 | |  |
| BDH1 | COPEB | | ERAL1 | | GATA1 | | JAK1 | | MAPK8IP1 | | NOV | | PRKCG | | SH2B3 | |  |
| BHD | COX18 | | ERBB2 | | GATA2 | | JAK2 | | MAPK9 | | NPM1 | | PRKCI | | SHC1 | |  |
| BIRC2 | CREBBP | | ERBB3 | | GATA3 | | JAK3 | | MAX | | NR4A3 | | PRKG1 | | SIN3A | |  |
| BIRC7 | CRIPAK | | ERBB4 | | GATA6 | | JUN | | MC1R | | NRAS | | PRMT5 | | SIRT3 | |  |
| BLM | CRKL | | ERCC1 | | GNA11 | | KAT6A | | MCL1 | | NSD1 | | PRX | | SKP2 | |  |
| BMPR1A | CRLF2 | | ERCC2 | | GNAQ | | KAT6B | | MDM2 | | NTRK1 | | PTCH1 | | SLIT2 | |  |
| BPTF | CSF1R | | ERCC3 | | GNAS | | KDM2A | | MDM4 | | NTRK2 | | PTCH2 | | SLX4 | |  |
| BRAF | CSMD1 | | ERCC4 | | GOLGA5 | | KDM4C | | MECOM | | NTRK3 | | PTEN | | SMAD2 | |  |
| BRCA1 | CTCF | | ERCC5 | | GOPC | | KDM5A | | MED12 | | ODZ2 | | PTK6 | | SMAD3 | |  |
| BRCA2 | CTNNB1 | | ERG | | GPC3 | | KDM5C | | MED12L | | OR5L1 | | PTP4A1 | | SMAD4 | |  |
| BRD1 | CUL2 | | ESR1 | | GPC5 | | KDM6A | | MED13 | | PAF1 | | PTP4A3 | | SMARCA2 | |  |
| BRD3 | CYC1 | | ESR2 | | GPR124 | | KDR | | MED29 | | PAK1 | | PTPN11 | | SMARCA4 | |  |
| BRD4 | CYLD | | ETS1 | | GRAF | | KEAP1 | | MEN1 | | PAK2 | | PTPRD | | SMARCB1 | |  |
| BRIP1 | CYP1B1 | | ETV1 | | GRB2 | | KIAA1549 | | MET | | PAK3 | | PTPRN2 | | SMC1A | |  |
| BUB1B | CYP2C19 | | ETV5 | | GRB7 | | KIAA2016 | | MIR142 | | PAK4 | | PTTG1IP | | SMC3 | |  |
| C11orf30 | CYP2C8 | | ETV6 | | GRID1 | | KIF5B | | MITF | | PAK6 | | RAB23 | | SMNDC1 | |  |
| C2orf44 | CYP2D6 | | EWSR1 | | GRIN2A | | KIT | | MLH1 | | PALB2 | | RAB25 | | SMO | |  |
| CADM2 | CYP3A4 | | EXT1 | | GRM3 | | KLF4 | | MLL | | PALB2 | | RAC1 | | SMURF1 | |  |
|  |  | |  | |  | |  | |  | |  | |  | |  | |  |
|  | | | | | |  | |  | |  | |  | |  | |  | |
| **CCPv1** | |  | | **CCPv2** | |  | |  | |  | |  | |  | |  | |
| SMYD3 | | VEGFA | | ABL1 | | CDC73 | | FANCD2 | | IGF1 | | MYB | | RAB35 | | TERC | |
| SOCS1 | | VEGFR1 | | ABL2 | | CDH1 | | FANCE | | IGF1R | | MYC | | RAC1 | | TERT | |
| SOX10 | | VEGFR2 | | ACVR1B | | CDK12 | | FANCF | | IGF2 | | MYCL1 | | RAD50 | | TET2 | |
| SOX17 | | VEZF1 | | ADA | | CDK4 | | FANCG | | IKBKE | | MYCN | | RAD51 | | TGFBR2 | |
| SOX2 | | VHL | | AKT1 | | CDK6 | | FANCL | | IKZF1 | | MYD88 | | RAF1 | | TK1 | |
| SOX9 | | WHSC1L1 | | AKT2 | | CDK8 | | FAS | | IL2RA | | NF1 | | RANBP2 | | TLE3 | |
| SPOP | | WNT10A | | AKT3 | | CDKN1A | | FAT1 | | IL7R | | NF2 | | RARA | | TMPRSS2 | |
| SRC | | WRN | | ALK | | CDKN1B | | FBXW7 | | INHBA | | NFE2L2 | | RASA2 | | TNFRSF10A | |
| SRSF2 | | WSB1 | | APC | | CDKN2A | | FGF10 | | INPP4B | | NFKB1 | | RB1 | | TNFRSF10B | |
| STAG2 | | WT1 | | AR | | CDKN2B | | FGF14 | | IRF2 | | NFKBIA | | RBM10 | | TNFRSF14 | |
| STAT3 | | WTX | | ARAF | | CDKN2C | | FGF19 | | IRF4 | | NKX2-1 | | RET | | TOP1 | |
| STAT5 | | WWOX | | AREG | | CEBPA | | FGF23 | | IRF7 | | NOTCH1 | | RHEB | | TOP2A | |
| STK11 | | XPA | | ARFRP1 | | CHD2 | | FGF3 | | IRS2 | | NOTCH2 | | RICTOR | | TP53 | |
| STK19 | | XPC | | ARID1A | | CHD4 | | FGF4 | | JAK1 | | NOTCH3 | | RNF169 | | TSC1 | |
| SUFU | | XPO1 | | ARID1B | | CHEK1 | | FGF6 | | JAK2 | | NPM1 | | RNF43 | | TSC2 | |
| SUV420H2 | | XRCC1 | | ARID2 | | CHEK2 | | FGFR1 | | JAK3 | | NRAS | | ROS1 | | TSHR | |
| TAF1 | | YAP1 | | ASXL1 | | CIC | | FGFR2 | | JUN | | NSD1 | | RPS27 | | TUBB3 | |
| TBL1XR1 | | YWHAB | | ATM | | CRABP2 | | FGFR3 | | KAT6A | | NTRK1 | | RPTOR | | TYMS | |
| TBX22 | | YWHAQ | | ATR | | CREBBP | | FGFR4 | | KDM5A | | NTRK2 | | RQCD1 | | U2AF1 | |
| TBX3 | | YWHAZ | | ATRX | | CRKL | | FH | | KDM5C | | NTRK3 | | RRM1 | | VEGFA | |
| TCF1 | | ZMYND11 | | AURKA | | CRLF2 | | FLCN | | KDM6A | | NUP93 | | RUNX1 | | VHL | |
| TDRD9 | | ZNF132 | | AURKB | | CSF1R | | FLT1 | | KDR | | OGFR | | RUNX1T1 | | WISP3 | |
| TERC | | ZNF217 | | AXIN1 | | CTCF | | FLT3 | | KEAP1 | | PAK3 | | RXRB | | WNT1 | |
| TERT | | ZNF278 | | AXL | | CTNNA1 | | FLT4 | | KEL | | PALB2 | | SDHA | | WT1 | |
| TET1 | | ZRSR2 | | BAP1 | | CTNNB1 | | FOLR2 | | KIT | | PARK2 | | SDHB | | WTX | |
| TET2 | |  | | BARD1 | | CUL3 | | FOXL2 | | KLHL6 | | PARP1 | | SDHC | | XPO1 | |
| TFG | |  | | BCL2 | | CYLD | | FOXM1 | | KNSTRN | | PAX5 | | SDHD | | ZBTB2 | |
| TGFBR2 | |  | | BCL2L1 | | DAXX | | FOXO1 | | KRAS | | PBRM1 | | SERPINB3 | | ZNF217 | |
| TIF1 | |  | | BCL2L2 | | DCK | | FOXP1 | | LRP1B | | PCDHGA1 | | SETD2 | | ZNF703 | |
| TLR4 | |  | | BCL6 | | DDR2 | | FRS2 | | LYN | | PDCD1LG2 | | SF3B1 | | ZNRF3 | |
| TMEM127 | |  | | BCOR | | DDX3X | | FUBP1 | | LZTR1 | | PDGFRA | | SHH | |  | |
| TMPRSS2 | |  | | BCORL1 | | DICER1 | | GABRA6 | | MAGI2 | | PDGFRB | | SLIT2 | |  | |
| TNFAIP3 | |  | | BCR | | DNMT3A | | GART | | MAP2K1 | | PDK1 | | SMAD2 | |  | |
| TNFRSF14 | |  | | BDNF | | DOT1L | | GATA1 | | MAP2K2 | | PGP | | SMAD3 | |  | |
| TNFRSF6 | |  | | BIRC5 | | EGFR | | GATA2 | | MAP2K4 | | PGR | | SMAD4 | |  | |
| TOP1 | |  | | BLM | | EIF1AX | | GATA3 | | MAP3K1 | | PIK3C2B | | SMARCA4 | |  | |
| TOP2A | |  | | BRAF | | EP300 | | GATA4 | | MAPK1 | | PIK3CA | | SMARCB1 | |  | |
| TP53 | |  | | BRCA1 | | EPHA3 | | GATA6 | | MAPK13 | | PIK3CB | | SMO | |  | |
| TP63 | |  | | BRCA2 | | EPHA5 | | GD2 | | MAPK2 | | PIK3CG | | SNCAIP | |  | |
| TP73 | |  | | BRD4 | | EPHA7 | | GID4 (C17orf39) | | MAPK3 | | PIK3R1 | | SNX31 | |  | |
| TPM3 | |  | | BRIP1 | | EPHB1 | | GLI1 | | MCL1 | | PIK3R2 | | SOCS1 | |  | |
| TPMT | |  | | BTG1 | | ERBB2 | | GNA11 | | MDM2 | | PLCG2 | | SOX10 | |  | |
| TRAF2 | |  | | BTK | | ERBB3 | | GNA13 | | MDM4 | | PLK1 | | SOX2 | |  | |
| TRAF7 | |  | | C11orf30 | | ERBB4 | | GNAQ | | MED12 | | PMS2 | | SOX9 | |  | |
| TRIM27 | |  | | C15ORF55 | | ERCC1 | | GNAS | | MEF2B | | PNP | | SPARC | |  | |
| TRIM28 | |  | | CARD11 | | ERCC2 | | GPR124 | | MEN1 | | POLA | | SPEN | |  | |
| TRIM33 | |  | | CASP8 | | EREG | | GRIN2A | | MET | | POLD1 | | SPOP | |  | |
| TRRAP | |  | | CBFB | | ERG | | GRM3 | | MGMT | | POLE | | SPTA1 | |  | |
| TSC1 | |  | | CBL | | ERRFI1 | | GRM5 | | MITF | | PPP2R1A | | SRC | |  | |
| TSC2 | |  | | CCND1 | | ESR1 | | GSK3B | | MLH1 | | PPP6C | | SSTR | |  | |
| TSHR | |  | | CCND2 | | ETV1 | | GSTP1 | | MLL | | PREX2 | | STAG2 | |  | |
| TSHZ2 | |  | | CCND3 | | ETV4 | | H3F3A | | MLL2 | | PRKAR1A | | STAT3 | |  | |
| TSHZ3 | |  | | CCNE1 | | ETV5 | | HGF | | MLL3 | | PRKCI | | STAT4 | |  | |
| TUBD1 | |  | | CD19 | | ETV6 | | HIF1A | | MPL | | PRKDC | | STK11 | |  | |
| U2AF1 | |  | | CD20 | | EWSR1 | | HNF1A | | MRE11A | | PRSS8 | | STK19 | |  | |
| UBE3A | |  | | CD274 | | EZH2 | | HRAS | | MRPS31 | | PTCH1 | | SUFU | |  | |
| UGT1A1 | |  | | CD33 | | FABP5 | | HSD3B1 | | MSH2 | | PTEN | | SYK | |  | |
| UHFR2 | |  | | CD52 | | FAM46C | | HSP90AA1 | | MSH6 | | PTGS2 | | TACC1 | |  | |
| USP9X | |  | | CD79A | | FANCA | | IDH1 | | MTOR | | PTPN11 | | TAF1 | |  | |
| VDAC2 | |  | | CD79B | | FANCC | | IDH2 | | MUTYH | | QKI | | TBX3 | |  | |
