# Supplementary material for: In vitro and in vivo drug screens of tumor cells identify novel therapies for high‐risk child cancer
Source: EMBO Mol Med. 2021 Dec 20;14(4):e14608. doi: 10.15252/emmm.202114608 (PMC8988207; doi:10.15252/emmm.202114608)
Supplement: Supplementary file 9 [file EMMM-14-e14608-s006.pdf]

EMBO Molecular Medicine

In vitro and in vivo drug screens of tumor cells identify novel therapies for high-risk child cancer

Loretta Lau, Chelsea Mayoh, Jinhan Xie, Paulette Barahona, Karen MacKenzie, Marie Wong, Alvin Kamili, Maria Tsoli, Tim Failes, Amit Kumar, Emily Mould, Andrew Gifford, Shu-Oi Chow, Mark Pinese, Jamie Fletcher, Greg Arndt, Dong-Anh Khuong-Quang, Carol Wadham, Georgina Eden, Peter Trebilcock, Swapna Joshi, Stephanie Alfred, Anjana Gopalakrishnan, Aaminah Khan, Dylan Grebert Wade, Patrick Strong, Elodie Manouvrier, Lisa Morgan, Roxanne Cadiz, Caitlin Ung, David Thomas, Katherine Tucker, Meera Warby, Geoffery McCowage, Luciano Dalla-Pozza, Jennifer Byrne, Federica Saletta, Andrew Fellowes, Stephen Fox, Murray Norris, Vanessa Tyrrell, Toby N. Trahair, Richard Lock, Mark Cowley, Paul Ekert, Michelle Haber, David Ziegler, and Glenn Marshall
DOI: 10.15252/emmm.202114608

Corresponding author: Glenn Marshall (g.marshall@unsw.edu.au)

Review Timeline:

Submission Date:	28th May 21
Editorial Decision:	29th Jun 21
Revision Received:	2nd Oct 21
Editorial Decision:	21st Oct 21
Revision Received:	25th Nov 21
Accepted:	1st Dec 21

Editor: Lise Roth

Transaction Report:

(Note: With the exception of the correction of typographical or spelling errors that could be a source of ambiguity, letters and reports are not edited. Depending on transfer agreements, referee reports obtained elsewhere may or may not be included in this compilation. Referee reports are anonymous unless the Referee chooses to sign their reports.)

29th Jun 2021

Dear Prof. Marshall,

Thank you for the submission of your manuscript to EMBO Molecular Medicine. We have now received feedback from the three reviewers who agreed to evaluate your manuscript. As you will see from the reports below, the referees acknowledge the interest of the study and are overall supporting publication of your work pending appropriate revisions.

Addressing the reviewers' concerns in full will be necessary for further considering the manuscript in our journal, and acceptance of the manuscript will entail a second round of review. EMBO Molecular Medicine encourages a single round of revision only and therefore, acceptance or rejection of the manuscript will depend on the completeness of your responses included in the next, final version of the manuscript. For this reason, and to save you from any frustrations in the end, I would strongly advise against returning an incomplete revision.

Revised manuscripts should be submitted within three months of a request for revision; they will otherwise be treated as new submissions, except under exceptional circumstances in which a short extension is obtained from the editor.

When submitting your revised manuscript, please carefully review the instructions that follow below. We perform an initial quality control of all revised manuscripts before re-review; failure to include requested items will delay the evaluation of your revision.

We require:

1) A .docx formatted version of the manuscript text (including legends for main figures, EV figures and tables). Please make sure that the changes are highlighted to be clearly visible.

2) Individual production quality figure files as .eps, .tif, .jpg (one file per figure). For guidance, download the 'Figure Guide PDF' (https://www.embopress.org/page/journal/17574684/authorguide#figureformat).

3) A .docx formatted letter INCLUDING the reviewers' reports and your detailed point-by-point responses to their comments. As part of the EMBO Press transparent editorial process, the point-by-point response is part of the Review Process File (RPF), which will be published alongside your paper.

4) A complete author checklist, which you can download from our author guidelines (https://www.embopress.org/page/journal/17574684/authorguide#submissionofrevisions). Please insert information in the checklist that is also reflected in the manuscript. The completed author checklist will also be part of the RPF.

5) Please note that all corresponding authors are required to supply an ORCID ID for their name upon submission of a revised manuscript.

6) It is mandatory to include a 'Data Availability' section after the Materials and Methods. Before submitting your revision, primary datasets produced in this study need to be deposited in an appropriate public database, and the accession numbers and database listed under 'Data Availability'. Please remember to provide a reviewer password if the datasets are not yet public (see https://www.embopress.org/page/journal/17574684/authorguide#dataavailability).

In case you have no data that requires deposition in a public database, please state so in this section. Note that the Data Availability Section is restricted to new primary data that are part of this study.

7) For data quantification: please specify the name of the statistical test used to generate error bars and P values, the number (n) of independent experiments (specify technical or biological replicates) underlying each data point and the test used to calculate p-values in each figure legend. The figure legends should contain a basic description of n, P and the test applied. Graphs must include a description of the bars and the error bars (s.d., s.e.m.).

8) We would also encourage you to include the source data for figure panels that show essential data. Numerical data should be provided as individual .xls or .csv files (including a tab describing the data). For blots or microscopy, uncropped images should be submitted (using a zip archive if multiple images need to be supplied for one panel). Additional information on source data and instruction on how to label the files are available at
.

9) Our journal encourages inclusion of *data citations in the reference list* to directly cite datasets that were re-used and obtained from public databases. Data citations in the article text are distinct from normal bibliographical citations and should directly link to the database records from which the data can be accessed. In the main text, data citations are formatted as

follows: "Data ref: Smith et al, 2001" or "Data ref: NCBI Sequence Read Archive PRJNA342805, 2017". In the Reference list, data citations must be labeled with "[DATASET]". A data reference must provide the database name, accession number/identifiers and a resolvable link to the landing page from which the data can be accessed at the end of the reference. Further instructions are available at .

10) We replaced Supplementary Information with Expanded View (EV) Figures and Tables that are collapsible/expandable online. A maximum of 5 EV Figures can be typeset. EV Figures should be cited as 'Figure EV1, Figure EV2" etc... in the text and their respective legends should be included in the main text after the legends of regular figures.
- For the figures that you do NOT wish to display as Expanded View figures, they should be bundled together with their legends in a single PDF file called *Appendix*, which should start with a short Table of Content. Appendix figures should be referred to in the main text as: "Appendix Figure S1, Appendix Figure S2" etc.
- Additional Tables/Datasets should be labeled and referred to as Table EV1, Dataset EV1, etc. Legends have to be provided in a separate tab in case of .xls files. Alternatively, the legend can be supplied as a separate text file (README) and zipped together with the Table/Dataset file.

See detailed instructions here:
.

11) The paper explained: EMBO Molecular Medicine articles are accompanied by a summary of the articles to emphasize the major findings in the paper and their medical implications for the non-specialist reader. Please provide a draft summary of your article highlighting
- the medical issue you are addressing,
- the results obtained and
- their clinical impact.

This may be edited to ensure that readers understand the significance and context of the research. Please refer to any of our published articles for an example.

12) For more information: There is space at the end of each article to list relevant web links for further consultation by our readers. Could you identify some relevant ones and provide such information as well? Some examples are patient associations, relevant databases, OMIM/proteins/genes links, author's websites, etc...

13) Author contributions: the contribution of every author must be detailed in a separate section (before the acknowledgments).

14) A Conflict of Interest statement should be provided in the main text.

15) Every published paper now includes a 'Synopsis' to further enhance discoverability. Synopses are displayed on the journal webpage and are freely accessible to all readers. They include a short stand first (maximum of 300 characters, including space) as well as 2-5 one-sentences bullet points that summarizes the paper. Please write the bullet points to summarize the key NEW findings. They should be designed to be complementary to the abstract - i.e. not repeat the same text. We encourage inclusion of key acronyms and quantitative information (maximum of 30 words / bullet point). Please use the passive voice. Please attach these in a separate file or send them by email, we will incorporate them accordingly.

Please also suggest a striking image or visual abstract to illustrate your article as a PNG file 550 px wide x 300-600 px high.

16) As part of the EMBO Publications transparent editorial process initiative (see our Editorial at http://embomolmed.embopress.org/content/2/9/329), EMBO Molecular Medicine will publish online a Review Process File (RPF) to accompany accepted manuscripts.
In the event of acceptance, this file will be published in conjunction with your paper and will include the anonymous referee reports, your point-by-point response and all pertinent correspondence relating to the manuscript. Let us know whether you agree with the publication of the RPF and as here, if you want to remove or not any figures from it prior to publication.
Please note that the Authors checklist will be published at the end of the RPF.

EMBO Molecular Medicine has a "scooping protection" policy, whereby similar findings that are published by others during review or revision are not a criterion for rejection. Should you decide to submit a revised version, I do ask that you get in touch after three months if you have not completed it, to update us on the status.

I look forward to receiving your revised manuscript.

Yours sincerely,

Lise Roth

Lise Roth, PhD
Editor
EMBO Molecular Medicine

***** Reviewer's comments *****

Referee #1 (Comments on Novelty/Model System for Author):

The technical presentation of the results can be improved as outlined in the remarks to the author
The comprehensiveness of the study has a high novelty value
As only the molecular profiling was used to guide the treatment of the patients and the in vitro and PDX drug response testing was not performed real-time, the medical impact is medium, but the study still hints at how future studies could be performed that could have major medical impact.
The manuscript is all about novel model systems and as such, the model systems are highly adequate.

Referee #1 (Remarks for Author):

In this manuscript, Lau and coworkers describe the studies of 56 pediatric high-risk cancer cases where the authors attempted to generate molecular as well as in vitro drug response profiling and PDX drug response data from cancer samples. They show that the combined profiling can generate information supporting specific treatments in a greater number of patients than molecular profiling alone. As a systematic approach, this is an important study and it highlights both the promises and challenges with a more holistic profiling approach of individual cancer patients to identify personalized optimized treatments. While the in vitro drug screening may hold the greatest promise in generating reasonably timely treatment predictions in combination with sequencing, the study also shows the challenges in applying this approach since only a relatively small percentage of samples are successfully expanded for the drug testing. More refined culture conditions for each individual type of cancer explored are presumably needed for higher success rates in the in vitro drug testing. Nevertheless, this study should be of great interest to the research community and could be suitable for publication in EMBO Molecular Medicine if a few points are addressed:

1. Many numbers and fractions of numbers are bounced around in the article, but it often becomes difficult to follow them. The manuscript would benefit greatly from being more explicit throughout spelling out exactly what numbers any fraction that is mentioned is based on. Some of the schematic figures are not easy to follow. Figure 1A is relatively clear, but a summary table of all samples highlighting which ones were sequenced, in vitro drug tested and PDX drug tested would be helpful. Figure 4A is very difficult to follow. It is at least not clear to this reviewer which of the patients were treated based on the sequencing information (and then whether in vitro and PDX data agreed with the molecular profiling-based drug selection). How was the tier system applied for the in vitro and PDX drug response? Perhaps two different tables are needed, one as the current 4A, which describes where potential treatments were identified and one where the applied treatments are considered, their clinical out come and whether the molecular and drug testing information were in agreement or not with the outcome of the applied treatment.
2. It is not clear to which extent the in vitro or PDX drug profiling agreed with the molecular profiling predictions (and the clinical outcome). A clearer summary of this information would give better understanding of whether the in vitro and PDX testing could both expand the number of cases where a potential suitable treatment could be found AND whether these approaches may give more accurate predictions of efficacious treatment than the molecular profiling (or not).
3. In the in vitro studies, it would have been interesting also to study the treatments that significantly affected the AUC without necessarily affecting the IC50 (same target as in other samples and therefore similar IC50, but deeper response). There seems to have been quite a few of these types of treatments (in the lower right quadrant of the plots in figure 2).
4. For RA-013, that exhibited sensitivity to ceritinib without having alterations in the ALK gene, what did the molecular profiling tell about known off-targets of ceritinib? See for example Kuenzi et al. Nat Chem Biol 13, 1222-1231 for detailed analyses of the pharmacological profile of ceritinib.

Referee #2 (Remarks for Author):

EMBO Molecular Medicine

Manuscript: In vitro and in vivo drug screens of tumor cells identify novel therapies for child cancer patients (ID# EMM-2021-14608)

This manuscript by Lau et al aims to more effectively match cancer-related biomarkers with clinical treatments. Here, the authors developed a rapid high-throughput drug screening platform pitted against human primary tumor tissue utilizing primarily (but not

exclusively) patient derived xenografts (PDXs). Of the 56 patients enrolled in the study, using these approaches the authors effectively identified new therapeutic strategies for a cohort of patients (10) for whom no targetable molecular lesions were uncovered during conventional molecular genomic analyses. Additionally, the authors report that treatment options were identified across the entire platform for 70% of the enrolled patients, with a subset implemented, with a notable level of benefit (29%). Also, importantly, the authors showed that for cases where targets were isolated by conventional molecular genetics (CMG), a subset of those cases showed no benefit in their tumor tissue platforms when tested against rational agents suggest by CMG, showing that their overall approach can argue both for and against treatment options. The authors conclude that their data indicate that in vitro and in vivo drug-screening of primary patient cells can increase therapeutic options and improve clinical outcomes for high-risk pediatric cancer patients.

Overall, this manuscript is very well communicated and reporst intriguing and compelling data consistent with their conclusions. Similar studies have been done in medical (adult) oncology, but not in pediatric oncology - important since children suffer from a remarkably different array of malignancies, many of which are unique to the pediatric phase of life. I strongly believe that this manuscript is topically suitable for publication in EMM, with only a few suggestions that might enhance the manuscript for the authors to consider (I will not label them as "major" or "minor", as these suggestions feel somewhere in between major or minor).

Suggestions:
1) Histopathology - I suggest that one opportunity that was missed in the manuscript was the lack of any histopathology of the treated PDX tumors. Clinical remission of neoplasms, including pediatric neoplasms, can be induced by cytotoxicity (which is generally more systemically harmful) or forced differentiation of the malignant precursor cells (for example, APML, Neuroblastoma) (aka, differentiation therapy, which is typically much less toxic overall). It would be VERY interesting to see if the new treatments options suggested by the new tissue platforms drive necrosis, apoptosis, or differentiation as the underlying biologic mechanism.

2) Those the authors identified and classified the genomic changes in the primary tumors by CMG approaches, where activating or inactivating encoding mutations where identified, the authors did not specifically list the precise encoded mutation. It would be helpful to have these data be available, which will likely be of some interest to the reading audience.

3) For Figures 3B-F, it is easy to visualize differences between the different treatment options profiled. Lacking, however, is any calculated significance (e.g., t-test/P-Values) between the individual experimental arms compared to controls. The authors should be able to provide these data.

Referee #3 (Remarks for Author):

Lau and collaborators describe a precision medicine platform for 56 highly aggressive (>30% cure rate) pediatric cancers that integrates genomics and transcriptomics with in vitro high throughput screens (HTS) of a library of 111 compounds (FDA-approved and currently in clinical trials) and in vivo pre-clinical trial in a subset of these tumors. Primary fresh tumor tissues included hematologic malignancies (14%), non-CNS solid tumors (38%) and CNS tumors (48%) that they subjected to either HTS or developed PDXs. From 46 fresh tumor samples, they developed primary cultures for CNS tumors and PDXs from Non-CNS tumors for 44 samples.
14 tumors were subjected to HTS including 7/31 primary samples expanded in vitro and 7/22 tumors expanded as PDXs with in order of success lymphomas > leukemia> CNS tumors. All 14 patients of the 29 with therapeutic options received treatment based on the results of the screen.
Most patients with DIPG, and HGG had progressive disease, one anaplastic leukemia had a complete response and one HGG had a partial response.
While I agree that this platform represents the first pediatric study integrating in vitro and in vivo drug screening in a clinical study, unfortunately most of the patients treated had their disease progress which raises the true potential for expanding therapeutic options and improve outcome.

Specific criticisms:

The authors comment on the challenge in developing PDXs from CNS tumors. They should refer to several manuscripts describing a better success rate in the development of PDOXs from primary patients CNS tumor samples including Smith et al, Acta Neuropathologica, 2020 and Brabetz et al., Nature Medicine, 2018. See also Rokita et al, Cell Reports, 2019.

Figure 3 shows results of the pre-clinical trials for several PDXs. Data for three tumors led to the treatment of patients harboring this tumor. RA-049 ALCL with ALK-NPM1 fusion for which the patient with Ceritinib had a complete response which did not correspond to the pre-clinical trial data. Two preclinical trials with WE-012 a Ewing Sarcoma and RA-002 a HGG showed that the tumors recur and indeed the patient's tumor progressed on treatment. It would be good in the Discussion to talk about the challenges in translated the in vitro and pre-clinical data in the clinic.

Figure 4: While complete, the 14 tumors for which patients received the drug(s) treatment should be provided separately from all

the other tumors for which patients did not receive treatment. It would be good to list the tumor type corresponding to the patient ID. Having to go to the Tables to know the tumor type is awkward and difficult to assess. One could also provide the tumor type corresponding to the patient ID in the legend.

One medulloblastoma RA-021 is listed in Table 6. What subgroup of tumor this corresponded to? If this tumor is sensitive to gemcitabine, it might be a Group3. The authors should provide the subgroup.

Supplementary Table 3 list 110 compounds in the title but 111 are listed as described in the text.

***** Reviewer's comments *****

Referee #1 (Comments on Novelty/Model System for Author):

The technical presentation of the results can be improved as outlined in the remarks to the author. The comprehensiveness of the study has a high novelty value. As only the molecular profiling was used to guide the treatment of the patients and the in vitro and PDX drug response testing was not performed real-time, the medical impact is medium, but the study still hints at how future studies could be performed that could have major medical impact.
The manuscript is all about novel model systems and as such, the model systems are highly adequate.

Referee #1 (Remarks for Author):

In this manuscript, Lau and coworkers describe the studies of 56 pediatric high-risk cancer cases where the authors attempted to generate molecular as well as in vitro drug response profiling and PDX drug response data from cancer samples. They show that the combined profiling can generate information supporting specific treatments in a greater number of patients than molecular profiling alone. As a systematic approach, this is an important study and it highlights both the promises and challenges with a more holistic profiling approach of individual cancer patients to identify personalized optimized treatments. While the in vitro drug screening may hold the greatest promise in generating reasonably timely treatment predictions in combination with sequencing, the study also shows the challenges in applying this approach since only a relatively small percentage of samples are successfully expanded for the drug testing. More refined culture conditions for each individual type of cancer explored are presumably needed for higher success rates in the in vitro drug testing. Nevertheless, this study should be of great interest to the research community and could be suitable for publication in EMBO Molecular Medicine if a few points are addressed:

1. Many numbers and fractions of numbers are bounced around in the article, but it often becomes difficult to follow them. The manuscript would benefit greatly from being more explicit throughout spelling out exactly what numbers any fraction that is mentioned is based on.

Response: We agree with the Reviewer and have throughout tried to follow a convention of giving a summary patient number for the subgroup in each results section, then removed percentages and referred to mostly patient numbers, rather than percentages.
This is now clarified throughout the manuscript and highlighted in yellow. In addition, the pie charts in Fig. 1B are now showing both numbers and percentages.

1. Page 5, line 14: Of the 56 samples, 46 were received fresh and triaged by the amount of available tissue.
2. Page 5, line 24: Of the 46 fresh samples, adequate cell numbers allowing upfront HTS were available in only three. We therefore proceeded to in vitro expansion of primary tumor cells in 31 fresh samples, …
3. Page 6, line 10: We attempted PDX murine models in 42 of the 46 fresh patient samples, with successful engraftment of 22 samples
4. Page 6, line 14: PDX engraftment was most successful in non-CNS solid tumors/lymphoma (15/18 attempts) followed by leukemia (3/4 attempts) and CNS tumors (4/20 attempts).
5. Page 6, line 21: In summary, we were able to conduct HTS and/or PDX testing (7 PDX only, 5 HTS only, and 12 PDX and HTS) in 24 of the 46 patients who provided a fresh sample
6. Page 7, line 9: Ninety of these 111 agents had existing pediatric dose and safety data.
7. Page 8, line 17: These data from HTS provided orthogonal confirmation of a targetable molecular abnormality (mutation, copy number, expression) in 17 of the 32 targeted hits

(Table 1). Importantly, the remaining 15 hits represented drug responses without prior molecular hallmarks of sensitivity to that drug.

8. Page 10, line 4: Of the 22 treatments suggested by prior molecular testing, 8 led to an objective response (Table 2).
9. Page 10, line 23: We then evaluated whether our four-part testing platform with molecular (DNA and RNA), HTS and PDX would increase treatment options for the overall group of 56 high-risk pediatric cancer patients, compared to molecular alone.
10. Page 11, line 14: Fourteen of 55 patients received the personalized treatment, with a clinical benefit rate in 4 (1 complete response, 2 partial responses, 1 stable disease)
11. Page 12, line 11: Overall, 39 of the 56 patients would have received at least one therapy option and most options were derived from one testing platform. Forty two of the 56 patients would have received new clinical information (32 therapeutic only; 4 therapeutic & diagnosis; 3 therapeutic & germline; 1 diagnosis & germline; 1 germline; 1 cease therapy) from the four-component diagnostic platform which could have changed their clinical management (Fig 4A).

Some of the schematic figures are not easy to follow. Figure 1A is relatively clear, but a summary table of all samples highlighting which ones were sequenced, in vitro drug tested and PDX drug tested would be helpful.

Response:
To improve clarity we have added a summary table of all samples, highlighting which samples were sequenced, in vitro drug tested and PDX drug tested as **Appendix Table S3** (page 6, line 23).

Figure 4A is very difficult to follow. It is at least not clear to this reviewer which of the patients were treated based on the sequencing information (and then whether in vitro and PDX data agreed with the molecular profiling-based drug selection). How was the tier system applied for the in vitro and PDX drug response? Perhaps two different tables are needed, one as the current 4A, which describes where potential treatments were identified and one where the applied treatments are considered, their clinical outcome and whether the molecular and drug testing information were in agreement or not with the outcome of the applied treatment.

Response:
To simplify this complex figure we have added a new table correlating molecular target, targeted therapy received, and clinical response with HTS and PDX results in the 14 patients who received molecular-guided treatment as **Table 3** (page 11; line 17)

Table 3 also includes 4 patients who received empiric therapy based on known effective therapies in their disease, correlated with HTS and/or PDX responses to those drugs. The following has been added to describe the correlation of clinical response with molecular, HTS and PDX testing (page 11; line 16 - 20):

> "When we correlated the clinical response with the prediction of response by either molecular, HTS or PDX (Table 3), we found 4/14 molecular, 4/5 HTS and 4/8 PDX predictions correctly forecast a response in the patient receiving that specific drug. This included prediction of response or non-response, and strongly supports the clinical relevance of HTS and PDX testing."

We used the same tier system as for molecular. For example, irinotecan was considered a Tier 1 hit in HTS for RA-019 (Ewing's sarcoma) given there is clinical trial evidence of efficacy of irinotecan in Ewing's sarcoma. Clofarabine was considered a Tier 1 hit in PDX for RA-004 (pre-B ALL) with clinical trial evidence of efficacy of clofarabine in pre-B ALL.

We have clarified this in the manuscript (page 11, line 4): "We used 5 tiers of therapy evidence as described in the iCAT study (Harris *et al*, 2016) for molecular, HTS or PDX drug sensitivity."

2. It is not clear to which extent the in vitro or PDX drug profiling agreed with the molecular profiling predictions (and the clinical outcome). A clearer summary of this information would give better understanding of whether the in vitro and PDX testing could both expand the number of cases where a potential suitable treatment could be found AND whether these approaches may give more accurate predictions of efficacious treatment than the molecular profiling (or not).

Response:
We have added a new table showing details of correlation between molecular aberrations and the 32 molecular drug hits has now been now added as **Table 1** (page 8; line 17), with the following modification to text:

> "These data from HTS provided orthogonal confirmation of a targetable molecular abnormality (mutation, copy number, expression) in 17 of the 32 targeted hits (Table 1)."

A new table showing correlation between drug sensitivity predicted by molecular testing and PDX responses has now been added as **Table 2** (page 9, line 13), with the following modification to text:

> Page 9, line 13 - "A total of 75 treatments were tested in these 19 PDXs and 22 of these treatments were based on prior molecular findings (Table 2)."
>
> Page 10, line 4 - "Of the 22 treatments suggested by prior molecular testing, 8 led to an objective response (Table 2)."

As described in response above, a new table (**Table 3**) (page 11; line 16) has been added to summarise the clinical predictive value of molecular, HTS and PDX.

The following has been added (Page 12, line 3) with **Appendix Table S8** as a new table:

> "HTS and PDX could also provide informative negative results. Despite a molecular result suggesting a treatment, accompanying HTS and PDX on the same patient would have correctly not supported the use of the drugs in 10 of the 24 patients (Appendix Table S8)."

3. In the in vitro studies, it would have been interesting also to study the treatments that significantly affected the AUC without necessarily affecting the IC50 (same target as in other samples and therefore similar IC50, but deeper response). There seems to have been quite a few of these types of treatments (in the lower right quadrant of the plots in figure 2).

Response:
We have examined the HTS data for instances where the z score for AUC is less than -2 while the z score for IC50 is greater than -2, representing data points in the lower right quadrant of the plots in Figure 2B. The drug response curves revealed flattening of the curve at higher doses, i.e., the highest doses did not correspond to increasing cell death. This particular finding did not correlate with drug category (chemotherapy vs. targeted agents), specific drug targets or specific drugs. It appears this scenario occurred slightly more frequently in brain tumor samples.

No change has been made to the text.

4. For RA-013, that exhibited sensitivity to ceritinib without having alterations in the ALK gene, what did the molecular profiling tell about known off-targets of ceritinib? See for example

Kuenzi et al. Nat Chem Biol 13, 1222-1231 for detailed analyses of the pharmacological profile of ceritinib.

Response:
We have further explored the off-targets of ceritinib as reported by Kuenzi et al. We did not identify mutations, fusion or copy number aberrations in any of the 4 off-target molecular lesions (IGF1R, FAK1, RSK1 and RSK2). However, in addition to increased ALK expression (as reported in the manuscript), this sample also had increased RNA expression of IGF1R. We have added this finding to page 8, line 13 in the text:

> "Of interest, the sample also had increased expression of IGF1R, a known off-target response to ceritinib (Kuenzi *et al*, 2017)."

Referee #2 (Remarks for Author):

EMBO Molecular Medicine

Manuscript: In vitro and in vivo drug screens of tumor cells identify novel therapies for child cancer patients (ID# EMM-2021-14608)

This manuscript by Lau et al aims to more effectively match cancer-related biomarkers with clinical treatments. Here, the authors developed a rapid high-throughput drug screening platform pitted against human primary tumor tissue utilizing primarily (but not exclusively) patient derived xenografts (PDXs). Of the 56 patients enrolled in the study, using these approaches the authors effectively identified new therapeutic strategies for a cohort of patients (10) for whom no targetable molecular lesions were uncovered during conventional molecular genomic analyses. Additionally, the authors report that treatment options were identified across the entire platform for 70% of the enrolled patients, with a subset implemented, with a notable level of benefit (29%). Also, importantly, the authors showed that for cases where targets were isolated by conventional molecular genetics (CMG), a subset of those cases showed no benefit in their tumor tissue platforms when tested against rational agents suggest by CMG, showing that their overall approach can argue both for and against treatment options. The authors conclude that their data indicate that in vitro and in vivo drug-screening of primary patient cells can increase therapeutic options and improve clinical outcomes for high-risk pediatric cancer patients.

Overall, this manuscript is very well communicated and reports intriguing and compelling data consistent with their conclusions. Similar studies have been done in medical (adult) oncology, but not in pediatric oncology - important since children suffer from a remarkably different array of malignancies, many of which are unique to the pediatric phase of life. I strongly believe that this manuscript is topically suitable for publication in EMM, with only a few suggestions that might enhance the manuscript for the authors to consider (I will not label them as "major" or "minor", as these suggestions feel somewhere in between major or minor).

Suggestions:
1) Histopathology - I suggest that one opportunity that was missed in the manuscript was the lack of any histopathology of the treated PDX tumors. Clinical remission of neoplasms, including pediatric neoplasms, can be induced by cytotoxicity (which is generally more systemically harmful) or forced differentiation of the malignant precursor cells (for example, APML, Neuroblastoma) (aka, differentiation therapy, which is typically much less toxic overall). It would be VERY interesting to see if the new treatments options suggested by the new tissue platforms drive necrosis, apoptosis, or differentiation as the underlying biologic mechanism.

Response:
We agree with the Reviewer that histologic evaluation of responding PDX tumors would have been valuable in understanding the biologic mechanism underlying treatment response. We felt that the PDX experiments would have needed to be specifically designed to address this question and allow harvesting of tumors at several timepoints during the course of PDX treatment for evaluation of treatment effect across time. It would have been particularly important to capture tissues at the critical time point when tumors were showing signs of regression or slowing of growth. However, for the purpose of this study, we were focussed on major signs of efficacy by monitoring tumor size only.

No change has been made to the text.

2) Those the authors identified and classified the genomic changes in the primary tumors by CMG approaches, where activating or inactivating encoding mutations where identified, the authors did not specifically list the precise encoded mutation. It would be helpful to have these data be available, which will likely be of some interest to the reading audience.

Response:
This detail has now been provided in **Expanded View Table EV3** (Page 11, line 10)

3) For Figures 3B-F, it is easy to visualize differences between the different treatment options profiled. Lacking, however, is any calculated significance (e.g., t-test/P-Values) between the individual experimental arms compared to controls. The authors should be able to provide these data.

Response:
P values for EFS has now been added to Figures 3B-F.

Referee #3 (Remarks for Author):

Lau and collaborators describe a precision medicine platform for 56 highly aggressive (>30% cure rate) pediatric cancers that integrates genomics and transcriptomics with in vitro high throughput screens (HTS) of a library of 111 compounds (FDA-approved and currently in clinical trials) and in vivo pre-clinical trial in a subset of these tumors. Primary fresh tumor tissues included hematologic malignancies (14%), non-CNS solid tumors (38%) and CNS tumors (48%) that they subjected to either HTS or developed PDXs. From 46 fresh tumor samples, they developed primary cultures for CNS tumors and PDXs from Non-CNS tumors for 44 samples.
14 tumors were subjected to HTS including 7/31 primary samples expanded in vitro and 7/22 tumors expanded as PDXs with in order of success lymphomas > leukemia> CNS tumors. All 14 patients of the 29 with therapeutic options received treatment based on the results of the screen. Most patients with DIPG, and HGG had progressive disease, one anaplastic leukemia had a complete response and one HGG had a partial response.
While I agree that this platform represents the first pediatric study integrating in vitro and in vivo drug screening in a clinical study, unfortunately most of the patients treated had their disease progress which raises the true potential for expanding therapeutic options and improve outcome.

Specific criticisms:

The authors comment on the challenge in developing PDXs from CNS tumors. They should refer

to several manuscripts describing a better success rate in the development of PDOXs from primary patients CNS tumor samples including Smith et al, Acta Neuropathologica, 2020 and Brabetz et al., Nature Medicine, 2018. See also Rokita et al, Cell Reports, 2019.

Response:
We thank the Reviewer for drawing attention to these papers reporting a success rate of CNS orthotopic PDX models between 30% and 56%, which is higher than that of ours (20%; 4/20). This greater success with engraftment could be related to direct implantation of tumor cells in these studies versus implantation of in vitro expanded primary cells adopted by our study highlighted in this paper on DIPG (Tsoli et al, J Neurooncol 2019). We utilised the in vitro expansion approach so that in vitro expanded primary cells from small brain tumour biopsies could be utilised for both HTS and PDX.

The following has now been added to discussion (page 14, line 6 - 12):

> "However, our CNS orthotopic PDX engraftment rate of 20% is lower than that described in the literature, with some studies reporting a success from 30 to 56% (Brabetz *et al*, 2018; He *et al*, 2021; Smith *et al*, 2020). This is likely related to inoculating in vitro expanded tumor cells in our study versus direct implantation of tumor cells in other studies, and such difference in success has been described in diffuse midline glioma (Tsoli *et al*, 2019). We adopted the former approach to allow use of in vitro expanded primary cells from small brain tumor biopsies for both HTS and PDX."

Figure 3 shows results of the pre-clinical trials for several PDXs. Data for three tumors led to the treatment of patients harboring this tumor. RA-049 ALCL with ALK-NPM1 fusion for which the patient with Ceritinib had a complete response which did not correspond to the pre-clinical trial data. Two preclinical trials with WE-012 a Ewing Sarcoma and RA-002 a HGG showed that the tumors recur and indeed the patient's tumor progressed on treatment. It would be good in the Discussion to talk about the challenges in translated the in vitro and pre-clinical data in the clinic.

Response:
RA-049 ALCL with ALK-NPM1 fusion:
We used PPTP criteria (Houghton et al, 2007) for in vivo objective response assessment. CR is defined as disappearance of measurable tumour mass for at least one time point. Objective response for ceritinib in this model was therefore categorised as CR due to complete tumor regression by day 7 of daily treatment (depicted by tumor volume graph in Figure 3) and correlated with patient's response. Importantly, ceritinib was only administered for 28 days in the PDX model while the patient received 3 months of treatment. Furthermore, while all ceritinib-treated animals subsequently relapsed off treatment, 28 days of treatment extended median EFS from 8 to 43 days. Details of PDX responses in each of 75 treatments in the 19 PDX models are provided in Appendix Table S6, and correlation between clinical outcome and HTS/PDX provided in the newly added **Table 2 and 3.**

As suggested by the Reviewer, challenges in translating the in vitro and pre-clinical data in the clinic are now added in Discussion (page 14, line 20):

> "However, significant challenges remain for translating preclinical drug testing results into the clinic, such as the correlations between clinical responses, animal and human pharmacokinetics, and in vitro and in vivo sensitivity signals."

Figure 4: While complete, the 14 tumors for which patients received the drug(s) treatment should be provided separately from all the other tumors for which patients did not receive treatment. It would be good to list the tumor type corresponding to the patient ID. Having to go to the Tables to know the tumor type is awkward and difficult to assess. One could also provide the tumor type corresponding to the patient ID in the legend.

Response:
A new table correlating molecular, HTS and PDX results with clinical responses in the patient receiving the drug has now been added to the new **Table 3** (page 11; line 17). The following has been added to describe the correlation of clinical response with molecular, HTS and PDX testing (page 11; line 16 - 20):

> "When we correlated the clinical response with the prediction of response by either molecular, HTS or PDX (Table 3), we found 4/14 molecular, 4/5 HTS and 4/8 PDX predictions correctly forecast a response in the patient receiving that specific drug. This included prediction of response or non-response, and strongly supports the clinical relevance of HTS and PDX testing."

Tumor type has also been added to Figure 4A.

One medulloblastoma RA-021 is listed in Table 6. What subgroup of tumor this corresponded to? If this tumor is sensitive to gemcitabine, it might be a Group3. The authors should provide the subgroup.

Response:
RA-021 is a group 3 medulloblastoma with MYC amplification and medulloblastoma subgroup is now included in Fig 2B, Fig 4A and tables.

However, this sample was not sensitive to gemcitabine in PDX (as shown in Appendix Table S6 and the graph below, which was not included in Fig 3). It also did not have differential sensitivity to gemcitabine in HTS by z score criteria (z score AUC = minus 1) (Figure 2B). To clarify, the HTS sensitivity in Figure 4A referred to cladribine, decitabine and etoposide (as shown in Fig 2B and Appendix Table S5) and was given a Tier 3I based on preclinical evidence previously reported in medulloblastoma (Morfouace et al, Cancer Cell 2015).
No change has been made to the text.

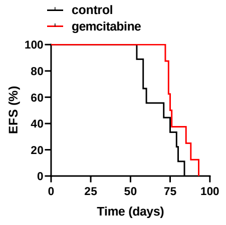

Supplementary Table 3 list 110 compounds in the title but 111 are listed as described in the text.

Response:
Supplementary Table 3 now becomes Table EV1 and title has been corrected to "111 compounds".

21st Oct 2021

Dear Prof. Marshall,

Thank you for the submission of your revised manuscript to EMBO Molecular Medicine. We have now received the enclosed reports from the three referees who re-reviewed your manuscript. As you will see, they are now supportive of publication, and I am therefore pleased to inform you that we will be able to accept your manuscript, once the following minor points will be addressed:

1/ Please discuss/address referee #3's remaining minor comment.

2/ Main manuscript text
- Please remove the yellow highlights in the text and only keep in track changes mode any new modification.
- Material and methods:
o Patients and samples: Please include a statement that the experiments conformed to the principles set out in the WMA Declaration of Helsinki and the Department of Health and Human Services Belmont Report.
o Cell culture: please indicate whether the cells were tested for mycoplasma contamination.
o Animals: please indicate the origin, age, gender of the mice, as well as their housing and husbandry conditions.
o Antibodies: please indicate the dilutions/concentrations used.

- Data Availability Section: Please only list here datasets that have been generated in this study. The link you provided refers to already published datasets that should be properly cited in the manuscript (https://actaneurocomms.biomedcentral.com/articles/10.1186/s40478-021-01248-w). Please clarify. Moreover, I note that you included tables with patients ID numbers. A permission is needed to access EGAS00001004572 to protect patients' personal data. Do you confirm that you have consent to share this information?
The section related to data availability in the checklist should be updated accordingly.

- Thank you for providing a "Conflict of interest" statement. Please remove "The authors declare that they have no conflict of interest" from the acknowledgements.

3/ Figures and Appendix:
- Statistics: Please indicate in the figures or in the legends the exact p= values, not a range, along with the statistical test used (including for ns, non-significant values). You may provide these values as a supplemental table in the Appendix file.
- Please upload Tables 1-3 as one file per table.
- Please make sure all figures and figure panels are referenced in the main manuscript file (callouts are currently missing for the panels of Figures 2, 3 and EV1).

4/ We would also encourage you to include the source data for figure panels that show essential data. Numerical data should be provided as individual .xls or .csv files (including a tab describing the data). For blots or microscopy, uncropped images should be submitted (using a zip archive if multiple images need to be supplied for one panel). Additional information on source data and instruction on how to label the files are available at
.

5/ Thank you for providing a nice synopsis picture. Please resize it (550 px wide/ 300-600px high) and adjust the font to stay legible.
I slightly shortened the text of your synopsis to fit our format, please let me know if you agree with the following:
"A precision diagnostic platform integrating genomics and transcriptomics with drug testing of patient's primary tumor cells in high throughput drug screening (HTS) and patient-derived xenograft (PDX) was established to improve identification of therapies in high-risk pediatric cancer patients.
• Treatment options could be identified for 70% of patients across the four-part platform.
• HTS provided orthogonal proof of drug efficacy suggested by molecular analyses and identified many new drug responses without prior molecular hallmarks.
• Effective treatments were observed in more than half of PDX models.
• There was a strong correlation between HTS and PDX results, and the clinical responses in patients.

6/ As part of the EMBO Publications transparent editorial process initiative (see our Editorial at http://embomolmed.embopress.org/content/2/9/329), EMBO Molecular Medicine will publish online a Review Process File (RPF) to accompany accepted manuscripts.
This file will be published in conjunction with your paper and will include the anonymous referee reports, your point-by-point response and all pertinent correspondence relating to the manuscript. Let us know whether you agree with the publication of the RPF and as here, IF YOU WANT TO REMOVE OR NOT any figures from it prior to publication.

Please note that the Authors checklist will be published at the end of the RPF.

I look forward to receiving your revised manuscript.

Yours sincerely,

Lise Roth

Lise Roth, PhD
Editor
EMBO Molecular Medicine

To submit your manuscript, please follow this link:

Link Not Available

The system will prompt you to fill in your funding and payment information. This will allow Wiley to send you a quote for the article processing charge (APC) in case of acceptance. This quote takes into account any reduction or fee waivers that you may be eligible for. Authors do not need to pay any fees before their manuscript is accepted and transferred to our publisher.

***** Reviewer's comments *****

Referee #1 (Comments on Novelty/Model System for Author):

The technical quality of the study has after the revision been considerably improved. The comprehensiveness of the study has a high novelty value. However, as only the molecular profiling was used to guide the treatment of the patients and the in vitro and PDX drug response testing was not performed real-time, the medical impact is medium. Nevertheless, the study still hints at how future studies can be performed that could have major medical impact. The manuscript is all about novel model systems and as such, the model systems are highly adequate.

Referee #1 (Remarks for Author):

The concerns raised by this referee in the first round of reviews have been adequately addressed. As a result, the manuscript is considerable improved and in my mind suitable for publication in EMBO Molecular Medicine.

Referee #2 (Comments on Novelty/Model System for Author):

The authors have improved this manuscript to where is ready for publication.

Referee #2 (Remarks for Author):

Though the authors have not been able to address all my comments regarding improvements to the manuscript, I appreciate and respect their thoughtful comments. And, they addressed my other comments that they could not address. In my opinion, the manuscript is suitable for publication in EMBO Molecular Medicine.

Referee #3 (Remarks for Author):

The authors have answers all my criticisms appropriately.
One note concerning gemcitabine. Gemcitabine does not work efficiently on its own but synergizes with other drugs including pemetrexed, a folate pathway inhibitor (Morfouace et al, Cancer Cell 2014) and prexasertib, a Chk1/Chk2 inhibitor (Endersby et al, Science Translational Medicine, 2021). Single agents have been shown to lead to recurrence and to be more effective in combination.

The authors performed the requested editorial changes.

1st Dec 2021

Dear Prof. Marshall,

Thank you for submitting your revised files. We are pleased to inform you that your manuscript is accepted for publication and is now being sent to our publisher to be included in the next available issue of EMBO Molecular Medicine!

Please read below for additional IMPORTANT information regarding your article, its publication and the production process.

Congratulations on your interesting work!

With kind regards,

Lise Roth

Lise Roth, Ph.D
Editor
EMBO Molecular Medicine

Follow us on Twitter @EmboMolMed
Sign up for eTOCs at embopress.org/alertsfeeds

EMBO PRESS

YOU MUST COMPLETE ALL CELLS WITH A PINK BACKGROUND ↓

PLEASE NOTE THAT THIS CHECKLIST WILL BE PUBLISHED ALONGSIDE YOUR PAPER

Corresponding Author Name: Glenn M Marshall
Journal Submitted to: EMBO Molecular Medicine
Manuscript Number: EMM-2021-14608-V3

USEFUL LINKS FOR COMPLETING THIS FORM

http://www.antibodypedia.com	Antibodypedia
http://1degreebio.org	1DegreeBio
http://www.equator-network.org/reporting-guidelines/improving-bioscience-research-repor	ARRIVE Guidelines
http://grants.nih.gov/grants/olaw/olaw.htm	NIH Guidelines in animal use
http://www.mrc.ac.uk/Ourresearch/Ethicsresearchguidance/Useofanimals/index.htm	MRC Guidelines on animal use
http://ClinicalTrials.gov	Clinical Trial registration
http://www.consort-statement.org	CONSORT Flow Diagram
http://www.consort-statement.org/checklists/view/32-consort/66-title	CONSORT Check List
http://www.equator-network.org/reporting-guidelines/reporting-recommendations-for-tumo	REMARK Reporting Guidelines (marker prognostic studies)
http://datadryad.org	Dryad
http://figshare.com	Figshare
http://www.ncbi.nlm.nih.gov/gap	dbGAP
http://www.ebi.ac.uk/ega	EGA
http://biomodels.net/	Biomodels Database
http://biomodels.net/miriam/	MIRIAM Guidelines
http://jjj.biochem.sun.ac.za	JWS Online
https://osp.od.nih.gov/biosafety-biosecurity-and-emerging-biotechnology/	Biosecurity Documents from NIH
http://www.selectagents.gov/	List of Select Agents

Reporting Checklist For Life Sciences Articles (Rev. June 2017)

This checklist is used to ensure good reporting standards and to improve the reproducibility of published results. These guidelines are consistent with the Principles and Guidelines for Reporting Preclinical Research issued by the NIH in 2014. Please follow the journal's authorship guidelines in preparing your manuscript.

A- Figures

1. Data

The data shown in figures should satisfy the following conditions:

- the data were obtained and processed according to the field's best practice and are presented to reflect the results of the experiments in an accurate and unbiased manner.
- figure panels include only data points, measurements or observations that can be compared to each other in a scientifically meaningful way.
- graphs include clearly labeled error bars for independent experiments and sample sizes. Unless justified, error bars should not be shown for technical replicates.
- if n< 5, the individual data points from each experiment should be plotted and any statistical test employed should be justified
- Source Data should be included to report the data underlying graphs. Please follow the guidelines set out in the author ship guidelines on Data Presentation.

2. Captions

Each figure caption should contain the following information, for each panel where they are relevant:

- a specification of the experimental system investigated (eg cell line, species name).
- the assay(s) and method(s) used to carry out the reported observations and measurements
- an explicit mention of the biological and chemical entity(ies) that are being measured.
- an explicit mention of the biological and chemical entity(ies) that are altered/varied/perturbed in a controlled manner.
- the exact sample size (n) for each experimental group/condition, given as a number, not a range;
- a description of the sample collection allowing the reader to understand whether the samples represent technical or biological replicates (including how many animals, litters, cultures, etc.).
- a statement of how many times the experiment shown was independently replicated in the laboratory.
- definitions of statistical methods and measures:
  - common tests, such as t-test (please specify whether paired vs. unpaired), simple χ2 tests, Wilcoxon and Mann-Whitney tests, can be unambiguously identified by name only, but more complex techniques should be described in the methods section;
  - are tests one-sided or two-sided?
  - are there adjustments for multiple comparisons?
  - exact statistical test results, e.g., P values = x but not P values < x;
  - definition of 'center values' as median or average;
  - definition of error bars as s.d. or s.e.m.

Any descriptions too long for the figure legend should be included in the methods section and/or with the source data.

In the pink boxes below, please ensure that the answers to the following questions are reported in the manuscript itself. Every question should be answered. If the question is not relevant to your research, please write NA (non applicable). We encourage you to include a specific subsection in the methods section for statistics, reagents, animal models and human subjects.

B- Statistics and general methods

Please fill out these boxes ↓ (Do not worry if you cannot see all your text once you press return)

1.a. How was the sample size chosen to ensure adequate power to detect a pre-specified effect size?	This pilot was designed to evaluate the feasibility of incorporating preclinical drug testing in a comprehensive precision oncology platform for children with high-risk cancer. We defined feasibility as 75% or more of the samples were available to attempt preclinical drug testing with priority for sequencing. A sample size of 55 would provide a 95% confidence interval of +/- 10% for a 75% preclinical testing attempt rate.
1.b. For animal studies, include a statement about sample size estimate even if no statistical methods were used.	At least 4 mice were included in each treatment arm for patient-derived xenograft drug testing to allow for detection of large differences in responses between control and treatment arm.
2. Describe inclusion/exclusion criteria if samples or animals were excluded from the analysis. Were the criteria pre-established?	Predefined inclusion criteria included informed consent from participants or their guardian; aged < 21 years with diagnosis of high-risk cancer (expected probability of survival < 30%) or age > 21 years with high-risk pediatric type cancers and prior approval from study chair; paired tumor and germline sample available. Predefined exclusion criteria for samples/patients included patients with non-high risk cancer diagnosis, absence of tumor tissue in submitted sample, and no tumor sample was available.
3. Were any steps taken to minimize the effects of subjective bias when allocating animals/samples to treatment (e.g. randomization procedure)? If yes, please describe.	This is an observational study testing the feasibility of implementing a precision oncology platform in a high-risk paediatric cancer population and therefore randomization of patients was not applicable. For individual patient-derived xenograft drug efficacy studies, no randomization were performed.
For animal studies, include a statement about randomization even if no randomization was used.	For patient-derived xenograft drug efficacy studies, allocation of treatment was not randomized.
4.a. Were any steps taken to minimize the effects of subjective bias during group allocation or/and when assessing results (e.g. blinding of the investigator)? If yes please describe.	For patient-derived xenograft drug efficacy studies, investigators were not blinded to treatment allocation.
4.b. For animal studies, include a statement about blinding even if no blinding was done	For patient-derived xenograft drug efficacy studies, investigators were not blinded to treatment allocation.
5. For every figure, are statistical tests justified as appropriate?	Yes. For Figure 2, Z scores were used to define diddferential sensitivity (drug hits) in high throughput drug screen using a 49 samples database. For Figure 3, P values for event-free survival (Kaplan and Meier) were shown in the figure with exact values listed in Appendix Table S6.
Do the data meet the assumptions of the tests (e.g., normal distribution)? Describe any methods used to assess it.	Yes, there is clear defintion of events and time to events when Kaplan and Meier method was used to estimate event-free survival in the animal studies.
Is there an estimate of variation within each group of data?	Not applicable. Analyses did not include estimate of mean or comparison of mean.
Is the variance similar between the groups that are being statistically compared?	Not applicable. Analyses did not include estimate of mean or comparison of mean.

C- Reagents

6. To show that antibodies were profiled for use in the system under study (assay and species), provide a citation, catalog number and/or clone number, supplementary information or reference to an antibody validation profile. e.g., Antibodypedia (see link list at top right), 1DegreeBio (see link list at top right).	PE mouse anti-human CD99 (Cat no. 130-104-315) (Miltenyi Biotec, Bergisch Gladbach, Germany), R-phycoerythrin (PE) mouse anti-human GD2 (Cat no. 562100); R-phycoerythrin (PE) mouse anti-human GD2 (Cat no. 562100) (Beckman Dickinson (BD) bioscience, New Jersey, USA); fluorescein isothiocyanate (FITC) mouse anti-human CD56 (Cat no. 562794) (Beckman Dickinson (BD) bioscience, New Jersey, USA); anti-allophycocyanin (APC) mouse anti-human CD45 (Cat no. 555485) (Beckman Dickinson (BD) bioscience, New Jersey, USA)<br>Manufacturer's data sheet information<br>Beckman Dickinson (BD) bioscience, New Jersey, USA<br>Anti-human GD2 (Cat no. 562100): Clone 14.G2a specifically reacts with human and mouse GD2 ganglioside. LAN-1 human neuroblastoma cells were used as immunogen. Clone 14.G2a is an isotype switch variant selected from the parental IgG3-producing hybridoma 14.18 and has identical reactivity as the parental antibody. Clone 14.G2a is routinely tested by flow cytometry using M21 human melanoma cells.<br>Anti-human CD56 (Cat no. 562794): Clone B19: immunogen – human NK cells; isotype: mouse IgG1, κ; reactivity: QC Testing - human; Workshop: V NK75; The B159 monoclonal antibody specifically binds to CD56. CD56 is a heavily glycosylated adhesion protein that is present on a subpopulation of peripheral blood large granular lymphocytes that demonstrate natural killer activity. CD56 is also expressed on a subset of T cells but is not expressed on myeloid cells, erythrocytes or B cells. This antigen is a pan-NK-cell marker.<br>Anti-human CD45 (Cat no. 555485): Clone HI30: immunogen - human peripheral blood leucocytes; isotype: Mouse IgG1, κ; reactivity: QC Testing – human; workshop: IV N816; The HI30 monoclonal antibody specifically binds to the 180, 190, 205, 220 kDa protein isoforms of CD45. CD45 is encoded by the PTPRC (Protein tyrosine phosphatase receptor type C) gene. CD45, also known as the leukocyte common antigen (LCA), is a member of the protein tyrosine phosphatase (PTP) family. It is present on all human leukocytes including lymphocytes, monocytes, granulocytes, eosinophils, and thymocytes. CD45 is absent from circulating erythrocytes, platelets, or mature erythroid cells of bone marrow and non-hemopoietic tissues.<br>Miltenyi Biotec, Bergisch Gladbach, Germany<br>Anti-human CD99 (Cat no. 130-104-315): Clone: 3B2/TA8: antigen: CD99; isotype: mouse IgG2aκ; isotype control: Mouse IgG2a – isotype control antibodies; distribution of antigen: T cells, thymocytes, plasma cells, other.
7. Identify the source of cell lines and report if they were recently authenticated (e.g., by STR profiling) and tested for mycoplasma contamination.	Not applicable. No cell lines were used.

* for all hyperlinks, please see the table at the top right of the document

D- Animal Models

8. Report species, strain, gender, age of animals and genetic modification status where applicable. Please detail housing and husbandry conditions and the source of animals.	Six- to eight-week-old, female, NOD/SCID/IL-2 receptor gamma-/- (NOD.Cg-Prkdcscid Il2rgtm1Wjl/SzJAusb; NSG) mice were purchased from Australian BioResources (Moss Vale, NSW, Australia) for non-CNS models and from Animal Resources Centre (Canning Vale, WA, Australia) for CNS models. Upon arrival, animals were housed in Translucent polycarbonate autoclavable cages (22 cm W x 15 cmH x 30 cm L Tecniplast, Italy) with air filters in positive pressure ventiracks. Bedding, enviro dry and igloos were provided for environmental enrichment. Irradiated rat and mouse breeder cubes and water were provided ad libitum.
9. For experiments involving live vertebrates, include a statement of compliance with ethical regulations and identify the committee(s) approving the experiments.	All animal studies complied with ethical regulations and were approved by the University of New South Wales Animal Ethics Committee.
10. We recommend consulting the ARRIVE guidelines (see link list at top right) (PLoS Biol. 8(6), e1000412, 2010) to ensure that other relevant aspects of animal studies are adequately reported. See author guidelines, under 'Reporting Guidelines'. See also: NIH (see link list at top right) and MRC (see link list at top right) recommendations. Please confirm compliance.	Compliance with ARRIVE guidelines confirmed.

E- Human Subjects

11. Identify the committee(s) approving the study protocol.	The study was approved by the Sydney Children's Hospitals Network Human Research Ethics Committee (LNR/14/SCH/497).
12. Include a statement confirming that informed consent was obtained from all subjects and that the experiments conformed to the principles set out in the WMA Declaration of Helsinki and the Department of Health and Human Services Belmont Report.	Informed consent was obtained from particpants aged over 18 and from parents for partipants aged under 18 years. The experiments conformed to the principles set out in the WMA Declaration of Helsinki and the Department of Health and Human Services Belmort Report.
13. For publication of patient photos, include a statement confirming that consent to publish was obtained.	Not applicable. No patient photos used for publication.
14. Report any restrictions on the availability (and/or on the use) of human data or samples.	Sequencing data has been deposited at the European Genome-phenome Archive (EGAD00001008358). Request for biologic material can be made by contacting zero@ccia.org.au and will be promptly reviewed by the Zero Childhood Cancer Research Management Committee. Any materials that can be shared will be released via a material transfer agreement.
15. Report the clinical trial registration number (at ClinicalTrials.gov or equivalent), where applicable.	Not applicable. This is not an interventional study.
16. For phase II and III randomized controlled trials, please refer to the CONSORT flow diagram (see link list at top right) and submit the CONSORT checklist (see link list at top right) with your submission. See author guidelines, under 'Reporting Guidelines'. Please confirm you have submitted this list.	Not applicable. This is not a Phase II/III clinical trial.
17. For tumor marker prognostic studies, we recommend that you follow the REMARK reporting guidelines (see link list at top right). See author guidelines, under 'Reporting Guidelines'. Please confirm you have followed these guidelines.	Not applicable. This is not a tumor marker prognostic study.

F- Data Accessibility

18: Provide a "Data Availability" section at the end of the Materials & Methods, listing the accession codes for data generated in this study and deposited in a public database (e.g. RNA-Seq data: Gene Expression Omnibus GSE39462, Proteomics data: PRIDE PXD000208 etc.) Please refer to our author guidelines for 'Data Deposition'.<br><br>Data deposition in a public repository is mandatory for:<br>a. Protein, DNA and RNA sequences<br>b. Macromolecular structures<br>c. Crystallographic data for small molecules<br>d. Functional genomics data<br>e. Proteomics and molecular interactions	A data availability section stating "Sequencing data are deposited at the European Genome-phenome Archive (EGAD00001008358; https://ega-archive.org/studies/EGAS00001004572)" can be found at the end of the Material and Methods.
19. Deposition is strongly recommended for any datasets that are central and integral to the study; please consider the journal's data policy. If no structured public repository exists for a given data type, we encourage the provision of datasets in the manuscript as a Supplementary Document (see author guidelines under 'Expanded View' or in unstructured repositories such as Dryad (see link list at top right) or Figshare (see link list at top right).	Sequencing data has been deposited at the European Genome-phenome Archive (EGAD00001008358).
20. Access to human clinical and genomic datasets should be provided with as few restrictions as possible while respecting ethical obligations to the patients and relevant medical and legal issues. If practically possible and compatible with the individual consent agreement used in the study, such data should be deposited in one of the major public access-controlled repositories such as dbGAP (see link list at top right) or EGA (see link list at top right).	Sequencing data has been deposited at the European Genome-phenome Archive (EGAD00001008358).
21. Computational models that are central and integral to a study should be shared without restrictions and provided in a machine-readable form. The relevant accession numbers or links should be provided. When possible, standardized format (SBML, CellML) should be used instead of scripts (e.g. MATLAB). Authors are strongly encouraged to follow the MIRIAM guidelines (see link list at top right) and deposit their model in a public database such as Biomodels (see link list at top right) or JWS Online (see link list at top right). If computer source code is provided with the paper, it should be deposited in a public repository or included in supplementary information.	Software and scripts related to this publication are available at https://github.com/CCICB/2020-hrPC-landscape.

G- Dual use research of concern

22. Could your study fall under dual use research restrictions? Please check biosecurity documents (see link list at top right) and list of select agents and toxins (APHIS/CDC) (see link list at top right). According to our biosecurity guidelines, provide a statement only if it could.	Not applicable.
